# Effect of Routine Cytochrome P450 2D6 and 2C19 Genotyping on Antipsychotic Drug Persistence in Patients With Schizophrenia: A Randomized Clinical Trial

**DOI:** 10.1001/jamanetworkopen.2020.27909

**Published:** 2020-12-07

**Authors:** Gesche Jürgens, Stig E. Andersen, Henrik B. Rasmussen, Thomas Werge, Heidi D. Jensen, Benjamin Skov Kaas-Hansen, Merete Nordentoft

**Affiliations:** 1 Clinical Pharmacological Unit, Zealand University Hospital, Roskilde, Denmark; 2 Department of Clinical Pharmacology, Bispebjerg and Frederiksberg University Hospitals, Copenhagen, Denmark; 3 Mental Health Centre Sct Hans, Roskilde, Denmark; 4 Copenhagen Research Center for Mental Health–CORE, Roskilde, Denmark

## Abstract

**Importance:**

Genetic polymorphism of genes encoding the drug metabolizing enzymes, cytochrome P450 2D6 and 2C19 (*CYP2D6* and *CYP2C19*), is associated with treatment failure of and adverse reactions to psychotropic drugs. The clinical utility of routine *CYP2D6* and *CYP2C19* genotyping (CYP testing) is unclear.

**Objective:**

To estimate whether routine CYP testing effects the persistence of antipsychotic drug treatment.

**Design, Setting, and Participants:**

This single-masked, 3-group randomized clinical trial included patients aged 18 years or older who had been diagnosed within the schizophrenic spectrum (*International Statistical Classification of Diseases and Related Health Problems, Tenth Revision *codes, F20-F29) and not previously genotyped. A total of 669 of 1406 potentially eligible patients from 12 psychiatric outpatient clinics in Denmark were approached between July 2008 and December 2009. Overall, 528 patients were genotyped and randomly allocated to 1 of 3 study groups or exclusion in a sequence of 1:1:1:3 using a predictive enrichment design, aiming to double the proportion of poor or ultrarapid metabolizers for CYP2D6 or CYP2C19. Outcome measurements were recorded at baseline and 1-year follow-up. Data analysis was performed in December 2012 and updated March 2019.

**Interventions:**

The trial included 2 intervention groups, where antipsychotic drug treatment was guided by either CYP test (CYP test–guided [CTG]) or structured clinical monitoring (SCM), in which adverse effects and factors influencing compliance were systematically recorded at least once quarterly, and 1 control group.

**Main Outcomes and Measures:**

Primary outcome was antipsychotic drug persistence, ie, days to first modification of the initial treatment. Secondary outcomes were number of drug and dose changes, adverse effects, and psychotic symptoms, ie, hallucinations and delusions.

**Results:**

A total of 528 participants were genotyped, and 311 (median [interquartile range {IQR} age, 41 [30-50] years; 139 [45%] women; median [IQR] duration of illness, 6 [3-13] years) were randomly allocated to 1 of 3 study groups. Overall, 61 participants (20%) were extreme metabolizers. There was no difference in antipsychotic drug persistence between the CTG group and the control group (hazard ratio [HR], 1.02; 95% CI, 0.71-1.45) or SCM and the control group (HR, 0.88; 95% CI, 0.61-1.26). Subanalyses among extreme metabolizers showed similar results (CTG: HR, 0.99; 95% CI, 0.48-2.03; SCM: HR, 0.93; 95% CI, 0.44-1.96).

**Conclusions and Relevance:**

The results of this randomized clinical trial do not support routine CYP testing in patients with schizophrenia.

**Trial Registration:**

ClinicalTrials.gov Identifier: NCT00707382

## Key Points

**Question:**

Does routine cytochrome P450 2D6 and 2C19 (*CYP2D6* and *CYP2C19*) genotyping affect the tolerability and effectiveness of antipsychotic drug treatment in patients with schizophrenia?

**Findings:**

In this randomized clinical trial with 311 participants, routine *CYP2D6* and *CYP2C10* genotyping had no beneficial effect on antipsychotic drug persistence, adverse drug reaction, or positive psychotic symptoms when compared with structured clinical monitoring and treatment as usual.

**Meaning:**

The results of this study do not support routine use of *CYP2D6* and *CYP2C19* genotyping in patients with schizophrenia.

## Teaser

This randomized clinical trial investigates whether routine *CYP2D6* and *CYP2C19* genotyping affects the persistence of antipsychotic drug treatment among adults with schizophrenia.

## Introduction

A significant proportion of psychiatric patients will be treated with psychotropic drugs metabolized by cytochrome P450 2D6 and 2C19 (CYP2D6 and CYP2C19) throughout their illness.^1^ Both enzymes are encoded by genetically polymorph genes, *CYP2D6* (OMIM 124030) and *CYP2C19 *(OMIM 124020). Their phenotypic expression is mostly determined by the number of functional alleles, and it divides populations into phenotype groups with different drug metabolizing capacities, ranging from poor metabolizers (PMs) to intermediate metabolizers (IMs), extensive metabolizers (EMs), and ultrarapid metabolizers (UMs). Genetic differences in drug metabolism of these enzymes is associated with both treatment failure and adverse drug reactions in psychiatric patients but also with distinctive prescription patterns and health care costs.^2,3,4^

Expectations of the beneficial effects of pharmacogenetic testing for *CYP2D6* and *CYP2C19* polymorphisms are largely based on theoretical considerations and extrapolations from studies focusing on the accuracy with which pharmacogenetic tests predict a clinical outcome. To our knowledge, the clinical utility of these tests has so far only been evaluated as part of a combined genetic approach in industry-sponsored studies in patients with major depression.^5,6^ Our aim was to assess whether routine genetic testing for *CYP2D6* and *CYP2C19* (CYP testing) improves antipsychotic drug treatment in patients with schizophrenia in terms of improved drug persistence, a surrogate for tolerability and effectiveness, compared with clinically guided treatment.

## Methods

This study is part of a Danish Health Technology Assessment that also includes a health economic analysis^4^ and an anthropological field study on patient-related and organizational factors affecting the use of the CYP test.^1^ The study protocol (Supplement 1) was reviewed and approved by an independent medical ethics committee and the Danish Data Protection Agency, and registered at ClinicalTrials.gov. Patients provided both written and verbal informed consent. Reporting followed the Consolidated Standards of Reporting Trials (CONSORT) reporting guideline for randomized clinical trials.

Patients or the public were not involved in design, conduct or reporting of the research. However, study results have been disseminated to participants.

### Study Design

This was a prospective, single-masked, 3-group, randomized clinical trial. We used a predictive enrichment strategy, aiming to double the proportion of extreme metabolizers (ExMs; individuals with reduced or fast metabolic capacity, ie, PMs or UMs). Individuals with intermediate and normal capacity, ie, IMs and EMs, will be referred to as normal metabolizers (NMs). The follow-up period was 1 year.

### Study Population and Sample Size

Patients were included consecutively from a pool of 1406 potentially eligible patients from 12 psychiatric outpatient clinics in the Capital Region, Denmark, until the desired sample size was achieved. Patients aged 18 years or older, diagnosed within the schizophrenic spectrum (*International Statistical Classification of Diseases and Related Health Problems, Tenth Revision *[*ICD-*10] codes, F20-F29), and not previously CYP tested were eligible for inclusion.

We based our sample size calculation on the need to make statistically valid comparisons of our primary outcome, antipsychotic drug persistence, between the subgroups of ExMs. We computed the needed number in each arm to be 20 (eAppendix 1 in Supplement 2).

### Intervention and Control Groups

There were 2 intervention groups, in which antipsychotic drug treatment was guided by either CYP testing (CYP test–guided [CTG]) or structured clinical monitoring (SCM), and 1 control group. In the CTG group, the CYP test results were revealed to the attending psychiatrists to guide pharmacological treatment. Their decision was supported by the CYP guideline that listed common antipsychotics and antidepressants and recommended gene-based dose adjustments. This guideline was published in 2006 when the CYP test became routinely available in the Capital Region, Denmark, and made available at the regional hospitals’ website (eAppendix 2 in Supplement 2).

In the SCM group, patients’ primary contact person (eg, nurses, psychologists, social workers) systemically recorded treatment effects, adverse effects, and attitudinal and behavioral factors influencing patient adherence. At least quarterly, a questionnaire containing validated questions from the Scale for the Assessment of Positive Symptoms (SAPS),^7^ UKU adverse effects score (Udvalg af Kliniske Undersøgelser),^8^ and Rating of Medical Influences^9^ was completed. Data served only as an intervention tool for the structured recording of information relevant for the medical treatment that could be passed on to the attending psychiatrists. In the control group, treatment followed usual clinical practice with no demands for assessment of symptoms at fixed terms. In both the SCM and control groups, the CYP test result remained concealed.

Prior to study start, project personnel repeatedly visited participating outpatient clinics, introducing the project, its interventions, and the CYP guideline. Before inclusion, both psychiatrist and primary contact person were notified in writing and asked to return a receipt of the notification, and patients in the CTG group received a statement regarding whether the CYP test result had an immediate consequence for the patient’s medical treatment. Receipt of the notification defined the patients’ inclusion date. Thus, the attending psychiatrists could adjust medicinal treatment at their own discretion prior to inclusion according to either CYP test result (CTG group) or best clinical judgment (SCM and control groups). This approach should ensure that relevant adaptation of pharmacological treatment upon receipt of the CYP test was not associated with reduced persistence. Drug concentration measurements as well as contacts to the regional Drug Information Service were permitted in all study groups.

### Inclusion, Randomization, and Masking

After informed consent, patients received CYP testing and were included in a stratified manner that ensured equal distribution of NMs and ExMs as well as individuals who used and did not use antipsychotic depot formulations between study groups. ExMs were randomly allocated to 1 of 3 study arms, whereas NMs were randomized to 1 of 3 study arms or to exclusion in a 1:1:1:3 sequence. Enrollment continued until 60 ExMs were included. Thus, each study group aimed to include 20 ExMs and 80 NMs, while 300 NMs were randomly excluded to elevate the proportion of ExMs from 10% to 20%.

Computer-generated lists with permutated blocks of 6 were used for randomization. Personnel involved in recruitment and examination of the study population were masked to patients’ allocation until the baseline examination was completed. Study personnel collecting analyzed baseline and follow-up data remained masked throughout.

### Genotyping

The genotype was analyzed in the Research Institute, Psychiatric Centre Sankt Hans Hospital. All samples were genotyped for the nonfunctional alleles CYP2D6*3, *4, *5 (gene deletion), and *6; *CYP2C19* *2 and *3; and *CYP2D6* gene duplications or multiplication according to previous published methods.^10,11,12^

In White participants, *CYP2D6* PM status is primarily attributed to the nonfunctional alleles *3, *4, *5, and *6,^13,14^ identifying 95% to 99% of all individuals with *CYP2D6* PM in European populations.^15^ Genotyping of the nonfunctional CYP2C19*2 and *3 alleles detects a high proportion of *CYP2C19* PM across different populations.^16^ PMs were defined by 0 functional alleles, IMs by 1 nonfunctional allele, EMs by 2 functional alleles, and UMs by duplicates of functional alleles. eAppendix 3 in Supplement 2 includes a sensitivity analysis analyzing the robustness of our outcome against phenotypical misclassification.

### Outcome Measures and Interrater Reliability

Outcome measures were registered at inclusion and after 12 months. Our primary outcome was antipsychotic treatment persistence, a surrogate measure integrating both possible therapeutic failure in UMs and dose-related adverse effects in PMs into a global measure of tolerability that is measurable in patients too ill to assist in a psychometric measurement, a subpopulation frequently omitted from trials. Persistence was measured as time in days to the first modification of the initial antipsychotic treatment, be it drug or dose change.

Our first secondary outcome was number of drug changes and number of drug and dose changes combined. Number of dose changes was estimated by 3 physicians (2 psychiatrists [M.N. and a second researcher] and 1 clinical pharmacologist [S.E.A.]) by visual inspection of temporal dose-adjustment graphs. Thus, sequential dose adjustment, assessed as planned dose titration, only counted as 1 dose change. Each case was discussed until agreement was reached.

The second secondary outcome was change in global rating of severity of hallucinations and delusions, using SAPS scores (range, 0-5).^7^ A positive difference between baseline and follow-up SAPS scores were coded as improvement; the resultant binary outcome variables for this outcome represented improvement vs nonimprovement.

Our final secondary outcome was change in severity of adverse effects, using the UKU scale,^8^ comprising 48 adverse effect items (range, 1-3 points) in the following 4 domains: psychiatric, neurological, autonomic, and others. Outcome variables were sums of differences between baseline and follow-up within each domain. We derived a combined score by summing the domain-specific differences. Positive values of the combined modeled score indicated improvement.

Interrater reliability was monitored for the assessment of SAPS and UKU.^17^ Six raters conducted interrater reliability sessions with a subsample of 20 participants, during which 1 rater interviewed the participants and the remaining raters completed the assessments.

### Explanatory Variables

The explanatory variables for the primary outcome were intervention groups vs control group. For changes in SAPS or UKU score, we used the following prespecified covariates with possible influence on the outcome: age, sex, special assertive early intervention, duration of illness, *CYP2D6*-dependent drug use, *CYP2C19*-dependent drug use, UKU score at baseline, hallucinations at baseline, delusions at baseline, PM status, and UM status.

### Statistical Analysis

Data analysis was performed in December 2012 and updated March 2019. R version 3.5.1 (R Project for Statistical Computing) was used for all analyses; an annotated analysis notebook appears in eAppendix 3 in Supplement 2. We used Cox regression to evaluate the effect of our intervention on antipsychotic drug persistence (primary outcome), (negative) binomial regression of the effect on the number of changes in drugs and doses (secondary outcome),^18^ logistic regression of the effect on severity of delusions and hallucinations (secondary outcome), and linear regression of the effect on adverse effects (secondary outcome). Patients who were randomized but failed to commence treatment were excluded from further analyses. For the Cox regression, we examined the proportional hazards assumption using Schoenfeld residuals. Significance levels were set to *P* < .05 in all analyses; all hypothesis tests were 2-sided.

SPSS statistical software version 5.5 for Windows (Microsoft Corp) was used to compute Cohen κ coefficients between all pairings of the 6 raters to gauge interrater reliability. Items with a post hoc kappa of greater than 0.61 advanced to additional rating sessions.^17^ Exact method was used to assess whether the observed genotype proportions deviated from those expected under conditions of Hardy-Weinberg equilibrium.^19^

## Results

### Study Population and Baseline Characteristics

Between July 2008 and December 2009, 669 eligible patients were asked to participate, and 528 (79%) consented and were genotyped. After randomization, 311 (59%) were included, of whom 61 (20%) were ExMs (41 [67%] *CYP2D6* PMs; 12 [20%] *CYP2D6* UMs; 8 [13%] *CYP2C19* PMs).

The median (interquartile range [IQR]) age was 41 (30-50) years; 139 (45%) were women; the most common diagnosis was paranoid schizophrenia (216 [70%]); and the median (IQR) duration of illness was 6 (3-13) years. A total of 21 patients (7%) never started treatment. This group was younger, included more men, and had shorter illness durations than the group that started treatment (eAppendix 3 in Supplement 2). Of the remaining 290 patients, 52 (18%) did not complete the follow-up examination. Table 1 contains descriptive demographic statistics of the participants and confirms randomization without substantial differences between the study groups.

**Table 1.** Characteristics and Descriptive Statistics of 311 Patients Retained in the Study After Inclusion

Characteristic	No. (%)		
	CTG group	SCM group	Control group
Patients	95 (33)	94 (32)	101 (35)
Persistence, median (IQR), d	170 (53 to 365)	253 (79 to 365)	206 (49 to 365)
Age, median (IQR), y	42 (32 to 51)	40 (30 to 48)	42 (31 to 53)
Women	43 (45)	43 (46)	46 (46)
Duration of illness, median (IQR), y	7 (3 to 15)	6 (3 to 16)	7 (3 to 13)
*CYP2D6* predicted phenotype			
Poor metabolizers	13 (14)	12 (13)	15 (15)
Intermediate metabolizers	30 (32)	32 (34)	33 (33)
Extensive metabolizers	47 (49)	46 (49)	50 (50)
Ultrarapid metabolizers	5 (5)	4 (4)	3 (3)
*CYP2C19* predicted phenotype			
Poor metabolizers	2 (2)	2 (2)	3 (3)
Intermediate metabolizers	25 (26)	24 (26)	20 (20)
Extensive metabolizers	68 (72)	58 (72)	78 (77)
Extreme metabolizers	20 (21)	18 (19)	21 (21)
Diagnoses			
F20, paranoid schizophrenia	68 (72)	73 (78)	66 (65)
F21, schizotypal disorder	19 (20)	13 (14)	20 (20)
F22, persistent delusional disorders	3 (3)	1 (1)	2 (2)
F23, acute and transient psychotic disorders	1 (1)	0	2 (2)
F25, schizoaffective disorders	2 (2)	4 (4)	8 (8)
F28, other nonorganic psychotic disorders	0	1 (1)	0 (0)
F29, unspecified nonorganic psychosis	0 (0)	1 (1)	0 (0)
Missing	2 (2)	1 (1)	3 (3)
Shifts in drugs and/or dose, No.			
0	34 (36)	39 (42)	37 (37)
1	14 (15)	20 (21)	17 (17)
2	17 (18)	12 (13)	16 (16)
3	7 (7)	5 (5)	8 (8)
≥4	23 (24)	18 (19)	23 (23)
Special assertive early intervention	15 (16)	16 (17)	15 (15)
*CYP2D6*-dependent drug use	77 (81)	82 (87)	82 (87)
*CYP2C19*-dependent drug use	18 (19)	23 (25)	25 (25)
Hallucinations improved	23 (24)	18 (19)	17 (17)
Delusions improved	30 (32)	29 (31)	17 (17)
Change in UKU score, median (IQR)^a^			
Autonomic	−1 (−2 to 1)	0 (−1 to 1)	0 (−2 to 1)
Neurological	0 (−1 to 1)	1 (−1 to 2)	1 (−1 to 2)
Psychiatric	1 (−2 to 3)	0 (−2 to 4)	0 (−2 to 3)
Other organ systems	0 (−2 to 1)	−1 (−2 to 2)	0 (−3 to 1)
All	2 (−4 to 5)	0 (−3 to 5)	0 (−5 to 5)

Abbreviations: CTG, cytochrome P450 test–guided; IQR, interquartile range; SCM, structured clinical monitoring; UKU, Udvalg af Kliniske Undersøgelser.

^a^ Negative changes indicate improvement, ie, lower adverse effect load at follow-up compared with baseline.

**Figure 1.**
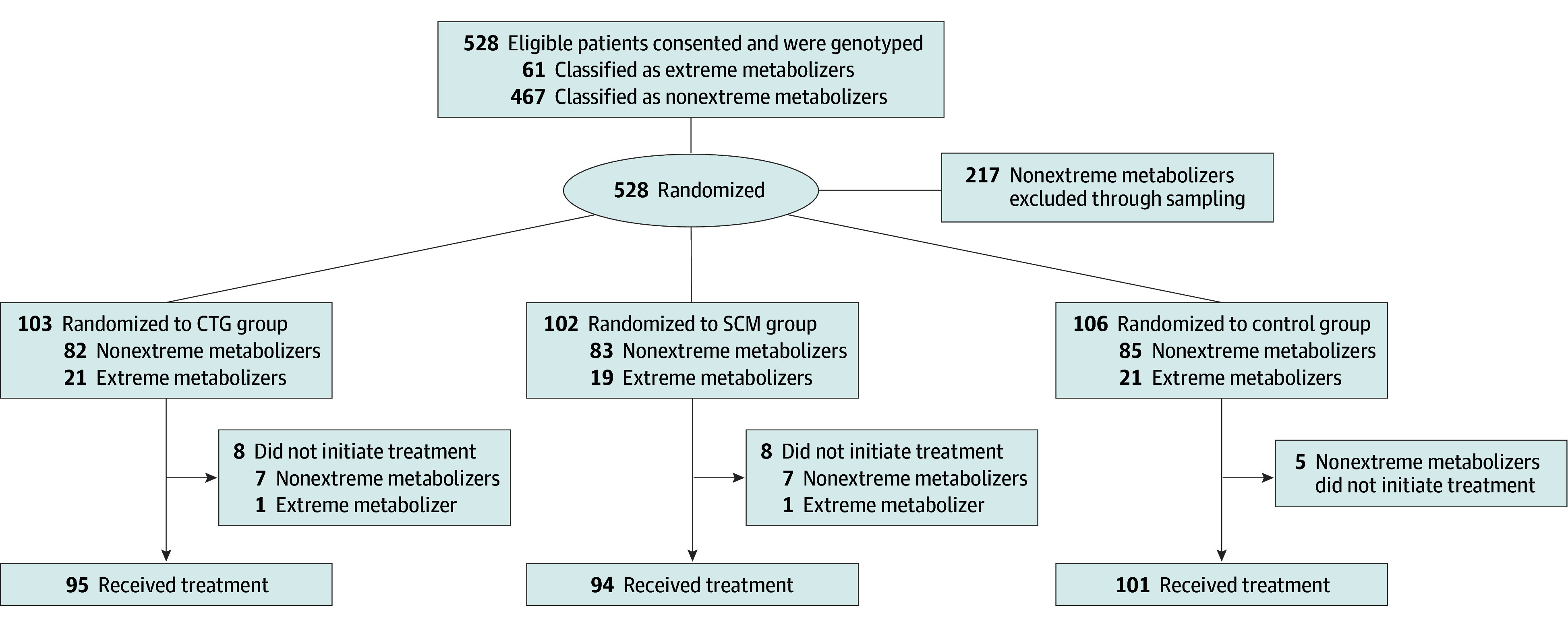
Flowchart of the Study Design CTG indicates cytochrome P450 test–guided treatment; SCM, structured clinical monitoring.

Figure 1 shows the flowchart of recruitment, enrollment, sampling, intervention, and follow-up. The primary outcome and the secondary outcomes of number of drug changes and number of drug and dose changes came from electronic patient records, yielding 0 patients lost to follow-up.

The genotype distributions did not deviate from those expected under Hardy-Weinberg conditions. However, a *P* = .05 was observed in the analysis of the distribution of *CYP2D6* genotypes with and without *4 (*4/*4, 22 participants [7%]; *4/non*4, 96 participants [31%]; non*4/non*4, 193 participants [62%]).

**Figure 2.**
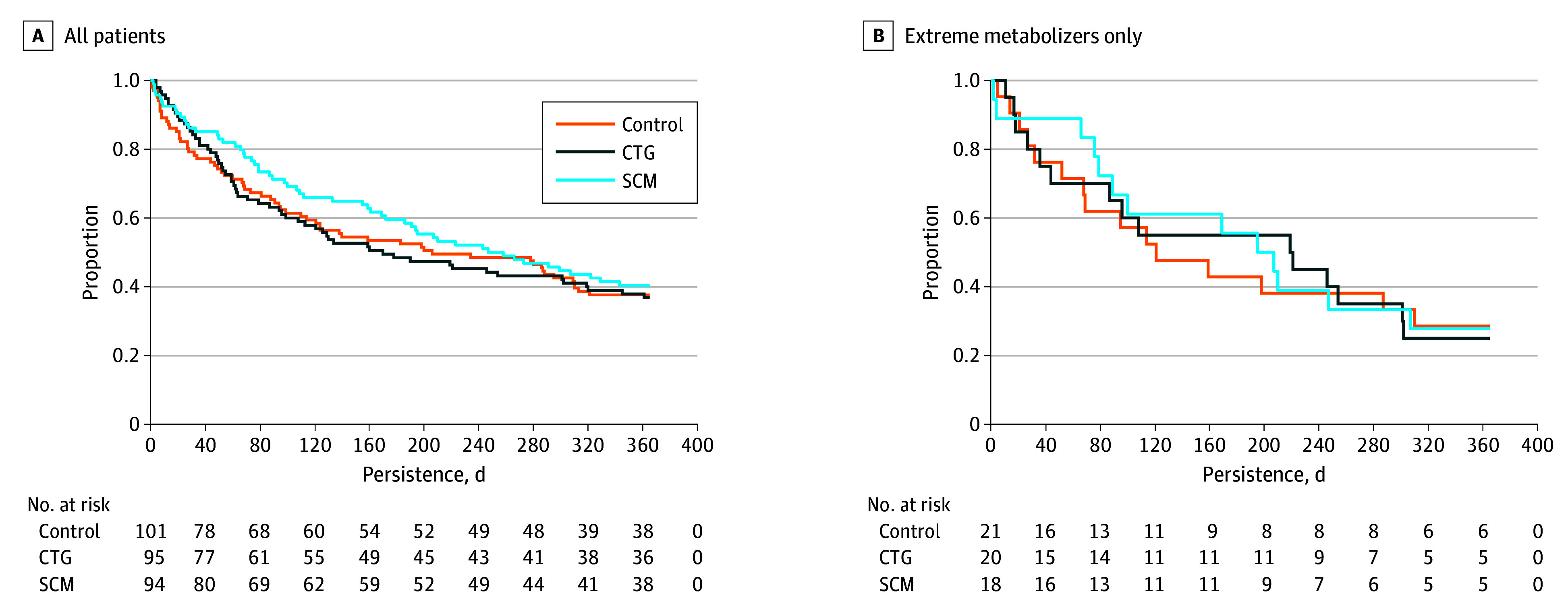
Persistence to Antipsychotic Drug Treatment CTG indicates cytochrome P450 test–guided treatment; SCM, structured clinical monitoring.

With the control group as the reference group, persistence in the CTG group (hazard ratio [HR], 1.02; 95% CI, 0.71-1.45) and SCM group (HR, 0.88; 95% CI, 0.61-1.26) were similar. ExMs in the CTG and SCM groups had similar HR estimates as well, albeit with wider confidence intervals (CTG: HR, 0.99; 95% CI, 0.48-2.03; SCM: HR, 0.93; 95% CI, 0.44-1.96) (eAppendix 3 in Supplement 2). Figure 2 illustrates that ExMs changed the original treatment earlier than NMs and that none of the interventions substantially affected the persistence after 1 year, rendering the HR estimates neither statistically significant nor clinically relevant. The Kaplan-Meier curves cross, but the plots of Schoenfeld residuals do not suggest that hazards be nonproportional (eAppendix 3 in Supplement 2).

The odds ratios (ORs) for at least 1 drug change in the CTG and SCM groups compared with the control group were similar (CTG: OR, 0.6; 95% CI, 0.4-17.6; SCM: OR, 0.5; 95% CI, 0.2-1.2). Few observations in ExMs impeded reliable parameter estimation for drug changes in this subset.

ExMs in the CTG and SCM group experienced fewer drug and dose changes combined than patients in control arm, with coefficients of −1.2 (95% CI, −4.1 to 1.2) and −2.3 (95% CI, −5.0 to −0.4), respectively. Coefficients arise from a negative binomial model and represent the estimated mean number of changes in each group compared with the control group. When including all patients, this effect diminished but remained statistically significant in the SCM group (−0.87; 95% CI, −1.85 to −0.01).

The results for psychotic symptoms are summarized in Table 2. There was no significant effect on hallucinations in the CTG group (OR, 2.20; 95% CI, 0.97-5.20) or the SCM group (OR, 1.38; 95% CI, 0.59-3.35). We found similar nonsignificant effects on delusions in the 2 intervention groups (CTG group: OR, 2.02; 95% CI, 0.94-4.49; SCM group: OR, 1.82; 95% CI, 0.84-4.05). The low pseudo-*R*^2^ values necessitate cautious interpretation of these results. We forewent planned analyses in ExMs due to data sparsity. We found no consistent effect on adverse effects; all confidence intervals contain 0, with quite large margins (Table 3).

**Table 2.** Improvement in Global Severity of Hallucinations and Delusions in All Patients, Using SAPS Scores Measured at Baseline and End of Follow-up, and Dichotomized Into Improvement vs Nonimprovement

Parameter	OR (95% CI)	
	Improvement in hallucinations	Improvement in delusions
Study group		
Control	1 [Reference]	1 [Reference]
CYP test–guided	2.20 (0.97 to 5.20)	2.02 (0.94 to 4.49)
Structured clinical monitoring	1.38 (0.58 to 3.36)	1.82 (0.84 to 4.05)
Age	1.01 (0.97 to 1.05)	0.97 (0.94 to 1.01)
Men	1.83 (0.92 to 3.73)	0.90 (0.48 to 1.69)
Illness duration	1.00 (0.95 to 1.05)	1.02 (0.97 to 1.07)
*CYP2D6*-dependent drug use	0.84 (0.39 to 1.85)	0.88 (0.43 to 1.83)
*CYP2C19*-dependent drug use	1.27 (0.56 to 2.79)	0.86 (0.39 to 1.83)
UKU score at baseline^a^	1.01 (0.97 to 1.05)	1.03 (1.00 to 1.07)
Poor metabolizer	0.67 (0.23 to 1.73)	2.08 (0.87 to 4.95)
Fast metabolizer	0.68 (0.03 to 5.41)	2.81 (0.43 to 22.6)
Nagelkerge pseudo-*R*^2^	0.06	0.09
Deviance	7.8^b^	13.2^b^
*P* value	.64	.21

Abbreviation: CYP, cytochrome P450; OR, odds ratio; SAPS, Scale for the Assessment of Positive Symptoms; UKU, Udvalg af Kliniske Undersøgelser.

^a^ Reflects the extent of adverse effects at beginning of follow-up; details appear in the Methods section.

^b^ Compared with intercept-only model without explanatory variables.

**Table 3.** Coefficients for Variables in Linear Models of Changes in Severity of Side Effects, in All Patients, Using Udvalg af Kliniske Undersøgelser Scores

Parameter	Coefficient estimate (95% CI)^a^				
	Autonomic	Neurologic	Psychiatric	Other	All
Study group					
Control	0 [Reference]	0 [Reference]	0 [Reference]	0 [Reference]	0 [Reference]
CYP test–guided	0.28 (−0.74 to 1.30)	−0.46 (−1.23 to 0.30)	0.40 (−1.07 to 1.88)	0.04 (−1.07 to 1.14)	0.35 (−2.48 to 3.17)
Structured clinical monitoring	0.58 (−0.46 to 1.63)	0.36 (−0.42 to 1.15)	0.64 (−0.88 to 2.15)	0.03 (−1.09 to 1.16)	1.61 (−1.29 to 4.50)
Age	−0.01 (−0.06 to 0.04)	0.01 (−0.02 to 0.05)	−0.04 (−0.11 to 0.03)	−0.01 (−0.07 to 0.04)	−0.05 (−0.18 to 0.08)
Men	0.55 (−0.39 to 1.49)	0.27 (−0.43 to 0.98)	0.31 (−1.05 to 1.68)	−0.30 (−1.31 to 0.72)	0.93 (−1.68 to 3.53)
Special assertive early intervention	0.30 (−1.13 to 1.72)	0.46 (−0.61 to 1.53)	−0.27 (−2.34 to 1.80)	−0.08 (−1.64 to 1.48)	0.34 (−3.66 to 4.34)
Duration of illness	0.03 (−0.03 to 0.09)	−0.01 (−0.05 to 0.04)	0.00 (−0.09 to 0.09)	0.05 (−0.02 to 0.12)	0.07 (−0.10 to 0.24)
*CYP2D6*-dependent drug use	0.77 (−0.33 to 1.86)	0.25 (−0.57 to 1.07)	0.03 (−1.56 to 1.62)	−1.09 (−2.28 to 0.09)	0.00 (−3.02 to 3.03)
*CYP2C19*-dependent drug use	−0.45 (−1.43 to 0.54)	−0.27 (−1.01 to 0.47)	1.58 (0.15 to 3.02)	0.62 (−0.45 to 1.68)	1.45 (−1.28 to 4.17)
Hallucinations at baseline^b^	−0.23 (−0.57 to 0.11)	0.26 (0.01 to 0.52)	0.13 (−0.36 to 0.62)	−0.07 (−0.43 to 0.30)	0.07 (−0.86 to 1.00)
Delusions at baseline^b^	0.14 (−0.19 to 0.46)	−0.18 (−0.43 to 0.06)	−0.27 (−0.74 to 0.20)	0.19 (−0.16 to 0.54)	−0.11 (−1.01 to 0.80)
Poor metabolizer	0.91 (−0.79 to 2.60)	0.19 (−1.09 to 1.46)	1.04 (−1.42 to 3.51)	1.02 (−0.81 to 2.84)	3.14 (−1.54 to 7.82)
Male poor metabolizer	1.18 (−2.46 to 4.83)	−1.59 (−4.32 to 1.15)	2.35 (−2.93 to 7.64)	−5.14 (−9.06 to −1.21)	−3.17 (−13.24 to 6.89)
Fast metabolizer	−2.64 (−4.91 to −0.38)	−0.39 (−2.09 to 1.31)	−0.27 (−3.56 to 3.02)	−1.60 (−4.04 to 0.84)	−4.97 (−11.23 to 1.29)
Male fast metabolizer	−4.72 (−9.56 to 0.13)	1.78 (−1.85 to 5.42)	−4.24 (−11.27 to 2.79)	3.07 (−2.15 to 8.29)	−4.22 (−17.60 to 9.16)
Stern *R*^2^ for goodness of fit	−0.06	−0.10	−0.11	−0.05	−0.12
F statistic^c^	1.4	0.81	0.70	1.6	0.62
*P* value	.16	.65	.77	.09	.85

^a^ Coefficients reflect the estimated change in Udvalg af Kliniske Undersøgelser score for a unit change in the corresponding covariate. Positive coefficients mean that a unit change in the corresponding covariates is associated with fewer side effects.

^b^ Used as numeric variable in this model for parsimony purposes.

^c^ Compared with intercept-only model without explanatory variables.

## Discussion

The goal of this investigator-initiated randomized clinical trial was to evaluate whether routine CYP testing can improve antipsychotic treatment in terms of tolerability and effectiveness in patients diagnosed with schizophrenia. We found no differences between study groups in antipsychotic drug persistence, neither in the full study population nor in the subgroup of ExMs.

Although not significant, there was a positive difference in measures of hallucination and delusion in the CTG groups. This finding is supported by an economic analysis^4^ that associated ExMs with higher costs and found that additional costs were avoided in the CTG group. Interestingly, we found a similar, albeit less pronounced, difference in the SCM group, challenging the notion of genotype guidance as the (sole) explanatory factor for improved antipsychotic effect. Indeed, these are secondary findings and must be interpreted with caution.

The clinical utility of *CYP2D6* and *CYP2C19* genotype–guided drug treatment has previously been addressed in a controlled manner as part of a combined genetic approach in 5 industry-sponsored studies focusing on the efficacy of antidepressants on major depressive disorder.^20,21,22,23,24^ Two pilot studies with an open-label design^20,21^ showed improved results on Hamilton Depression Rating Scale in the genotype-guided arm compared with the control group; however, these results were not reproduced in a masked confirmatory randomized clinical trial.^24^ Another randomized clinical trial^22^ showed a tendency toward a higher responder rate in participants whose treatment was genotype guided, but no differences in the primary outcome, sustained response. The fifth study,^23^ using a test solely focusing on genes that affect pharmacokinetics (*CYP2D6*, *CYP2C19*, *UGT1A1 *[OMIM 191740], and ABC transporters), showed a remission rate of 70% in the genotype-guided group compared with 28% in the control group when studied in a 12-week, double-masked randomized clinical trial with 148 participants. This is higher than what is commonly seen in efficacy studies of antidepressants, where remission rates are usually less than 50%^25^ but comparable with efficacy measures seen after more than 1 antidepressant trial.^26^ Differences in the number and selection of genes in different genetic test batteries and inconsistent findings makes it difficult to assess the true clinical value of these tests.^5,6^ None of these studies considered that their outcomes might be confounded by the fact that the gene test might have increased the clinicians’ attention toward a patient’s medical treatment and not as medical decision support. Furthermore, the clinical validity of several of these genotypes is not sufficiently substantiated to clearly support medical decision-making.^27^

Clearly, a combined approach allows for detecting more potential pharmacogenetic problems but also creates the possibility for more erroneous medical decisions and might, in theory, lead to unjustifiably withholding effective pharmacological treatment from the patient. It remains to be proven which (if any) approach should prevail.

From a decision-making perspective, the CYP test is not a 1-step solution that relates 1 test result to 1 therapeutic target dose. Phenotypic distinction of individuals with different CYP genotypes may be blurred because of overlap between pharmacokinetic parameters and possible phenotype conversion.^28^ Attempts to estimate dose adjustments for psychotropic drugs are based on average doses adjusted to mean kinetic parameters or to numbers of functional alleles.^29,30^ Guidelines for dose adjustment of *CYP2D6-* and CYP2C19-dependent drugs^31,32^ show inconsistencies.^33,34^ However, these estimates do not reflect the previously mentioned variability and should therefore only be considered as adjusted starting points for subsequent clinical titration. Thus, CYP test results must be interpreted in light of patients’ clinical presentation and the pharmacokinetic and dynamic properties of the chosen drug.

Small numbers of ExMs often hamper even large-scale studies assessing the clinical impact of *CYP2D6* and/or *CYP2C19* genotyping in psychiatric patients. To remedy this, we enriched our study population to double the prevalence of ExMs compared with the general population.

Presumably, ExMs benefit most from CTG treatment. However, no data substantiate that CYP tests truly influence the complex process of personalized prescription in psychiatric patients. We cannot exclude unintended harmful consequences, such as decreased clinical attention to adverse drug reactions or treatment failure in NMs. Thus, the evaluation of CTG treatment must include comparisons in both groups.

Switching to other drugs and changing dosage regimens can be regarded as an overall expression of unsatisfactory response to psychotropic drug treatment.^3,35^ Considering existing evidence, the nature of the CYP test, and the fact that patients with schizophrenia might be difficult to retain in studies, we judged that antipsychotic drug persistence was an appropriate outcome measure.

Both routine and SCM represent response-guided approaches. However, the use of a structured communicative tool in the SCM groups avoided favoring the CGT group by involuntarily increasing the attending physician’s attention to the patient's medication in this group. This confounder has significantly limited the interpretation of the results of the previously described utility studies.^20,21,22,23,24^

As a holistic approach, clinical monitoring can detect adverse effects and treatment failure, even when they are unrelated to the patient’s drug metabolism. In contrast, CYP genotyping mainly serves to identify patients at risk of relative underdosage or overdosage due to abnormal drug metabolism.

### Limitations

This study has limitations. Optimal utilization of CYP test information depends on prescribers’ knowledge and correct interpretation of the test. Different physicians might have handled test results differently, which may affect its optimal utilization. Furthermore, antipsychotic drug persistence is a surrogate measure that cannot elucidate the underlying cause of changes in the medication.

## Conclusions

In this randomized clinical trial, routine *CYP2D6 *and *CYP2C10 *genotyping had no effect on antipsychotic drug persistence. These results do not support routine use of genotyping for *CYP2D6* and *CYP2C19* polymorphisms in patients with schizophrenia.

## Notes

Supplement 1.
Trial Protocol

Supplement 2. **eAppendix 1. **Sample Size Calculation **eAppendix 2. **CYP Guideline **eAppendix 3. **Analysis Notebook

Supplement 3.
Data Sharing Statement
