# Supplementary material for: Effect of Routine Cytochrome P450 2D6 and 2C19 Genotyping on Antipsychotic Drug Persistence in Patients With Schizophrenia: A Randomized Clinical Trial
Source: JAMA Netw Open. 2020 Dec 7;3(12):e2027909. doi: 10.1001/jamanetworkopen.2020.27909 (PMC12520709; doi:10.1001/jamanetworkopen.2020.27909)
Supplement: Supplement 1. — Trial Protocol [file jamanetwopen-e2027909-s001.pdf]

Study protocol

Title

The effect of CYP2D6 and CYP2C19 genotyping and intensified clinical monitoring on antipsychotic drug treatment - a prospective randomized controlled trial

Background

Antipsychotic drug treatment in patients with schizophrenia is complicated by poor compliance (= adherence to drug prescription) and treatment persistence (= proportion of patients remaining in treatment within a defined time period). It is unclear whether this is caused by the frequent occurrence of adverse drug reactions, varying treatment effect, or due to poor disease insight and treatment motivation, but presumably it is a combination of different factors.

Compliance is important for both disease prognosis, relapse prevention and admission rate (1, 2). Like treatment persistence, it is used as an overall clinical outcome measure for the tolerability and effectiveness of antipsychotics in psychiatric research (3). In recent years, research has focused on both pharmacological and non-pharmacological (psychosocial) interventions that improve treatment persistence and compliance and thus the clinical treatment course in this patient population.

Psychotropic drugs are usually dosed individually according to the observed treatment effect and adverse drug reactions. A large proportion of antipsychotic drugs and drugs used as antipsychotic adjuvants are metabolized by Cytochrome P450 2D6 and 2C19 (CYP2D6 and CYP2C19). Both enzymes are coded by genes that exhibits genetic polymorphism. Genetic polymorphism results in significant variability of drug metabolism and drug concentration. 5 - 10% of the Caucasian population have a CYP2D6 genotype that is associated with reduced enzyme activity (poor metabolizer (PM)) compared to normal (extensive metabolizers (EM)), while approx. 1% have a significantly increased enzyme activity (ultra-rapid metabolizers (UM)). For CYP 2C19, the proportion of PMs is approx. 2-3% (4). It is well documented that PMs achieve significantly higher drug concentrations (5) and have an increased risk of developing adverse drug reactions (7-9), while UMs risk treatment failure (6). A health economic study in psychiatric patients shows that PM and UM status is associated with increased health care costs compared to normal metabolizers (10). This has led to considerations whether CYP2D6 and 2C19 genotyping (CYP test) could lead to an individualized and more tolerable psychotropic drug treatment and should be used routinely (5, 11, 12). However, drug concentrations within one genotype group may vary significantly. Clearly, the CYP genotype is not the only factor influencing drug concentration and tolerability (13). Furthermore, unintended negative consequence of the routine-use of the CYP test, such as decreased clinical observation of adverse drug reactions in patients with normal CYP genotype, cannot be ruled out.

The implementation of early intervention services has shown that nonpharmacological interventions, such as an intensified treatment alliance between patient and psychiatrist affects drug treatment positively. Patients treated by early intervention teams received on average 20% less antipsychotic drugs than those who received usual treatment in psychiatric outpatient clinics. At the same time, patients had fewer psychotic

symptoms and fewer admission days (14). Furthermore, The National Indicator Project has shown that the majority of patients with schizophrenia suffers from adverse drug reactions (15). At the same time, there are major differences in quality and comprehensiveness of adverse drug reaction registration. Thus, in only 23% of cases, sexual side effects were recorded. Therefore, we presume that increased focus on a regular and structured record of adverse drug reactions and effectiveness involving the patient's perception of drug treatment can lead to an improved treatment alliance and influence drug compliance and treatment persistence, drug tolerability and effectiveness.

There are currently no controlled clinical trials comparing genotype guided antipsychotic drug treatment with the clinically guided drug treatment.

Purpose

- To investigate whether CYP test guided treatment improves antipsychotic drug treatment in patients with schizophrenia compared to clinical dose titration.
- To investigate whether intensified clinical monitoring focusing on tolerability, effectiveness and patient's perception of medical treatment can improve antipsychotic drug treatment in patients with schizophrenia compared to control.
- To compare the effect of CYP test guided treatment and intensified clinical monitoring on antipsychotic drug treatment in patients with schizophrenia.

Design

A prospective blinded randomized trial designed as parallel study with three study arms; two intervention arms and one control arm. The study duration is 12 months. Outcome measures are recorded at baseline and 12 months follow up.

Study Population

300 patients diagnosed within the schizophrenic spectrum, defined as disorders within the F2 spectrum of ICD-10 (16); 60 extreme metabolizers (PM or UM for CYP2D6 or CYP2C19) and 240 normal metabolizers (EM or intermediate metabolizers (IM) for CYP2D6 or CYP2C19).

The sample size calculation is based on the need to make statistically sound comparisons between the smallest subgroup of the study population, i.e. extreme metabolizers. The calculation is based on the primary outcome "time to discontinuation of initial antipsychotic treatment". Based on the literature, we estimated the smallest relevant difference (d0) to be 45 days and the standard deviation of this parameter to be 39 3. With a type-1 error of 5% and a type 2 error of 10%, n is calculated from the formula: $n1 = n2 = n3=2 (t2\alpha + t2\beta)^2$ *

$SD^2 / d0^2 = 19.5$, i.e. 20 extreme metabolizers in each study arm. Assuming a prevalence of 10%, approximately 600 patients must be screened to identify 60 extreme metabolizers.

Inclusion criteria

• Age ≥ 18 years
• Capable of taking care of their own affairs
• Diagnosed with a psychiatric condition within the schizophrenic spectrum (F2, ICD-10) by a specialist in psychiatry
• Able to give informed consent
• Not previously CYP tested

Exclusion criteria

• Previously CYP-tested

Primary outcome measures:

a. Time to discontinue of initial antipsychotic drug treatment.
b. Persistence for treatment, i.e. percentage of patients remaining in treatment within a defined time period

Secondary effect parameters:

a. Compliance. (Participants are categorized as fully, partially or non-compliant based primarily on the patient's own report in a non-judgmental interview. In addition to that, information from serum concentration measurements are collected and patient's understanding of the importance of medical treatment is rated using Rating of Medication Influences (ROMI) scale (18).
b. Number of drug changes / dose changes
c. Positive symptoms in schizophrenia (Scale of Assessment of Positive Symptoms (SAPS)) score (17).
d. Side effect score (Udvalg af Kliniske Undersøgelser (UKU) score) (18), weight, metabolic functions (plasma cholesterol, solid blood sugar, plasma triglyceride, plasma prolactin (only when recorded routinely in the patient’s Personal Electronic patient Profile (PEM))
e. Number and length of admissions during the study period

Procedures

<u>Training of staff personnel</u>

Prior to the study start, the staff of the outpatient clinics in the Capital Region and other relevant project personnel is offered a training program. The training program includes registration of adverse drug reaction registration by means of an abbreviated version of the UKU (18), systematic questioning of psychotic symptoms using selected parts of SAPS (17) and the use of selected parts of an interview to elucidate the patient's understanding of the importance of medical treatment, Rating of Medication Influences (ROMI) (19). It also includes guidance on how the CYP test result can be used to direct psychopharmacological treatment (Appendix 1).

Recruitment, randomization

Study participants are recruited among patients who are affiliated with the outpatient clinics in the Capital Region. After informed consent, patients are genotyped for CYP2D6 and 2C19. Following primary stratification according to the genotype and use of antipsychotic depot preparations, patients are randomized to one out of three study arms or exclusion from the study. Inclusion continues until there are 20 extreme metabolizers are included in each study arm. Based on an expected frequency of 10% poor metabolizers, a total of 600 patients needs to be screened, of which 100 (20 extreme and 80 normal metabolizers) are included in each study arm and 300 are randomly excluded.

Blinding

In study arm 1 the result of the CYP test is openly revealed to psychiatrists in charge of treatment, while both patients and psychiatrists in study arm 2 and 3 are blinded to the CYP test result. Study personnel involved in the rating of patients at baseline and follow up will be blinded to the patients' study arm allocation and CYP test result. Study personnel performing statistical analyses will be blinded to the study arm allocation.

Intervention and treatment

In study arm 1 the genotype information is given to the physician in charge of treatment and can be used to direct the psychopharmacological treatment in accordance with the current guidelines from Sct. Hans hospital (Appendix 2 a and 2 b). In the guidelines, the genotype is translated to the clinical designation "normal", "slow" or "fast" metabolizer of CYP2D6 or "normal" or "slow" metabolizer of CYP2C19. Different treatment options for the different genotypes are described in the clinical guidelines.

In study arm 2 the genotype information is not revealed. The intervention consists of an intensified clinical monitoring of treatment effect, side effects and patient perspective. Staff personnel is trained in the use of a clinical manual that builds on a selection of validated questions from the Scale for the Assessment of Positive Symptoms (SAPS (17)), Side effect score (Udvalg af Kliniske Undersøgelser (UKU (18) and Rating of Medical Influences (ROMI (19)) (Appendix 3). The manual has to be used at least once in a quarter (every third month), which is monitored by the study personnel. Data registered by the patients' primary contact person are not used as outcome measures in the study but only as intervention tool for the optimization of the medical antipsychotic treatment.

In study arm 3, (Control arm) treatment followed usual local practice. The genotype information is not revealed.

To support medical decisions in all study arms the physician in charge of treatment and the primary contact person is allowed to contact the Drug Information Service at the Clinical Pharmacological Unit at Bispebjerg University Hospital, weekdays between 9 and 14 to obtain information on antipsychotics CYP2C19 / CYP2D6 dependency, need for dose adjustment and interpretation of drug concentration measurements, etc. Still, the final treatment decision is made by the physician in charge of treatment.

Data recording

Immediately after the inclusion, the following information is recorded:

- Demographic data (age, gender, weight and height)
- History of the disease
- Symptom score (SAPS score)
- Side effect score (Udvalg af kliniske undersøgelser (UKU) score)
- Medical history (current and historical)
- Compliance (ROMI)

The following information is registered 12 months after study conclusion:

- Symptom score (SAPS score)
- Side effect score (Udvalg af klinisk undersøgelser (UKU) score)
- Compliance (ROMI)
- Weight

- Metabolic functions (plasma cholesterol, fasting blood glucose, plasma triglyceride, plasma prolactin (only when available in PEM)

Handling of biological material

Blood samples for the analysis of metabolic functions and serum concentration measurement of antipsychotics are routine tests and part of the clinical follow-up of patients receiving antipsychotics. Blood samples are analyzed by the Clinical Biochemical Department at Bispebjerg Hospital or the laboratories the hospital usually collaborates with. Blood samples are destroyed after analysis.
Blood samples for genotype analysis are sent to the Research Institute at Sct. Hans Hospital in Roskilde. The blood samples will after data anonymization be included in the Research Institute's biobank.

Data processing

Data comparisons are made both between the two study arms, but also between the subgroups of extreme and normal metabolizers in each study arm. Due to the unpaired and presumably nonparametric character of our data, we plan to use Mann-Whitney's rank sum test, chi squared test and log rank test hypothesis testing. For continuous data, ANOVA is used. The level of significance is 5%. Since the selected power measurements are interdependent, it is not necessary to correct for the risk of mass significance.

Target group for the project

Professional, political, and organizational decision makers, i.e. specialists in psychiatry, politicians and hospital administrators.

Publication

The project is designed as a PhD project and will be published as PhD thesis. In addition, parts of the project will be included in the Medical Technology Assessment "Does dose individualization by genotyping improve the drug antipsychotic treatment?". Both positive and negative results of the experiment will be published. Decisions on authorship and co-authorship on all publications that originate from this project will be made in accordance with the Vancouver Recommendations.

Ethical considerations

Prior to the start-up of all project-related activities, approvals are obtained from the Scientific Ethics Committee. All patient-related information is recorded in an anonymized form and handled in accordance with

the Act on Processing of Personal Data and the Act on Patients' Legal Status. Relevant scientific ethical guidelines are followed. The trial is reported to the Danish Data Agency.

The CYP test is based on a single blood sample and without further disadvantages for the patient. The CYP test is fully implemented at Sct. Hans Hospital and the clinical guideline for use and interpretation of the CYP test results has been thoroughly tested in a highly specialized clinical setting. The CYP test is used only for dose guidance and has no influence on the patient's disease prognosis, illness or death. Therefore, the test is not expected to create significant ethical problems in relation to the patient. Since the clinical effect of genotyping compared to the clinical dose titration is unknown, the randomization to a study arm were CYP test results cannot be used for drug choice or dose management is not expected to involve ethical problems for the patient or the treating physician. Moreover, the trial intervention is not associated with risks or disadvantages for the patient.

Principle investigators

Gesche Jürgens, MD, Clinical Pharmacological Unit, Bispebjerg University Hospital.
Merete Nordentoft, Consultant, MD, PhD, Psychiatric Department, Bispebjerg University Hospital.

Collaborators

Stig Ejdrup Andersen, Consultant, MD, PhD, Clinical Pharmacological Unit, Bispebjerg University Hospital.
Thomas Werge, Head of Research, PhD, Research Institute, Sct. Hans Hospital.
Henrik Berg Rasmussen, Senior Researcher, PhD, Research Institute, Sct. Hans Hospital,
Søren Bredkjær, Medical Director, Sct. Hans Hospital.

Project organization and finances

The project was initiated by Gesche Jürgens, Stig Ejdrup Andersen and Merete Nordentoft and received financial support from The Danish National Board of Health and TRYG Fonden. There is no financial link between researchers and private companies, foundations, etc., with interests in the outcome of the research project in question.
All funding is administered by the Finance Department, Bispebjerg University Hospital and used for salaries and administrative expenses. No money is paid to patients participating in the trial.

The project is part of a Medical Technology Assessment (HTA), an interdisciplinary assessment of the clinical,

economic and socio-cultural effects of a routine use of the genetic test in psychiatry. The project is a collaboration between the Clinical Pharmacological Unit and Psychiatric Department E at the Bispebjerg University Hospital, Copenhagen, the Research Institute of Biological Psychiatry at the Sct. Hans Hospital in Roskilde and the Danish Health Institute (DSI) in Copenhagen.

Reference List

1. Thieda P, Beard S, Richter A, Kane J. An economic review of compliance with medication therapy in the treatment of schizophrenia
1. Psychiatr Serv 2003; 54(4):508-516.

2. Zygmunt A, Olfson M, Boyer CA, Mechanic D. Interventions to improve medication adherence in schizophrenia. Am J Psychiatry 2002; 159(10):1653-1664.

3. Lieberman JA, Stroup TS, McEvoy JP et al. Effectiveness of antipsychotic drugs in patients with chronic schizophrenia. N Engl J Med 2005; 353(12):1209-1223.

4. Brosen K. Drug-metabolizing enzymes and therapeutic drug monitoring in psychiatry. Ther Drug Monit 1996; 18(4):393-396.

5. Kirchheiner J, Brosen K, Dahl ML et al. CYP2D6 and CYP2C19 genotype-based dose recommendations for antidepressants: a first step towards subpopulation-specific dosages. Acta Psychiatr Scand 2001; 104(3):173-192.

6. Kirchheiner J, Henckel HB, Meineke I, Roots I, Brockmoller J. Impact of the CYP2D6 ultrarapid metabolizer genotype on mirtazapine pharmacokinetics and adverse events in healthy volunteers. J Clin Psychopharmacol 2004; 24(6):647-652.

7. Chen S, Chou WH, Blouin RA et al. The cytochrome P450 2D6 (CYP2D6) enzyme polymorphism: screening costs and influence on clinical outcomes in psychiatry. Clin Pharmacol Ther 1996; 60(5):522-534.

8. de LJ, Barnhill J, Rogers T, Boyle J, Chou WH, Wedlund PJ. Pilot study of the cytochrome P450-2D6 genotype in a psychiatric state hospital. Am J Psychiatry 1998; 155(9):1278-1280.

9. Vandel P, Haffen E, Vandel S et al. Drug extrapyramidal side effects. CYP2D6 genotypes and phenotypes. Eur J Clin Pharmacol 1999; 55(9):659-665.

10. Chou WH, Yan FX, de LJ et al. Extension of a pilot study: impact from the cytochrome P450 2D6 polymorphism on outcome and costs associated with severe mental illness. J Clin Psychopharmacol 2000; 20(2):246-251.

11. De Sundhedsfaglige Råd i Psykiatri i Københavns Amt og H:S. Cytochrom P450 genotypetest af psykiatriske patienter. 2006.
Ref Type: Report

12. Shastry B. Pharmacogenetics and the concept of individualized medicine. The Pharmacogenomics Journal 2006; 6:16-21.

13. Reis M, Prochazka J, Sitsen A, Ahlner J, Bengtsson F. Inter- and intraindividual pharmacokinetic variations of mirtazapine and its N-demethyl metabolite in patients treated for major depressive disorder: a 6-month therapeutic drug monitoring study. Ther Drug Monit 2005; 27(4):469-477.

14. Petersen L, Jeppesen P, Thorup A et al. A randomised multicentre trial of integrated versus standard treatment for patients with a first episode of psychotic illness
1. BMJ 2005; 331(7517):602.

15. Nordentoft M, Voldgaard I, Poulsen HD, Linneberg A, Mainz J. Det Nationale Indikatorprojekt: Skizofreni-Standarder, indikatorer og prognostiske faktorer. 2001.
Ref Type: Report

16. World Health Organization. The ICD-10 Classification of Mental and Behavioral Disorders. Diagnostic criteria for research. 1993.
Ref Type: Report

17. Andreasen NC. Methods for assessing positive and negative symptoms. Mod Probl Pharmacopsychiatry. 1990; 73-88.

18. Lingjaerde O, Ahlfors UG, Bech P, Dencker SJ, Elgen K. The UKU side effect rating scale. A new comprehensive rating scale for psychotropic drugs and a cross-sectional study of side effects in neuroleptic-treated patients. Acta Psychiatr Scand Suppl 1987; 334:1-100.

19. Weiden P, Rapkin B, Mott T et al. Rating of medication influences (ROMI) scale in schizophrenia. Schizophr Bull 1994; 20(2):297-310.

Appendix 1 Guidance on the use of the CYP test in the dosing of the drug treatment (11)

**Predicted phenotype**	**Clinical Guide**
Normal metabolizer (EM) of CYP2D6:	• Standard dose of CYP2D6 dependent preparations<br>• No difference between CYP2D6 dependent and independent preparations
Slow metabolizer (PM) of CYP2D6:	• Observation of adverse reactions of CYP2D6 dependent medicinal products<br>• Regular determination of serum concentration<br>• Lower dosage of CYP2D6 dependent drugs than the standard dose<br>• Switch to CYP2D6 independent preparations
Fast metabolizer (UM) of CYP2D6:	• Observation for the lack of efficacy of CYP2D6 dependent drugs<br>• Regular determination of serum concentration<br>• Higher dosage of CYP2D6 dependent drugs than the standard dose<br>• Switch to CYP2D6 independent preparations
Normal metabolizer (EM) of CYP2C19:	• Standard dose of CYP2C19 dependent drugs<br>• No difference between CYP2C19 dependent and independent preparations
Slow metabolizer (PM) of CYP2C19:	• Observation of adverse reactions of CYP2C19 dependent drugs<br>• Regular determination of serum concentration<br>• Lower dosage of CYP2C19 dependent drugs than the standard dose<br>• Switch to CYP2C19 independent preparations
