# Supplementary material for: Effect of Routine Cytochrome P450 2D6 and 2C19 Genotyping on Antipsychotic Drug Persistence in Patients With Schizophrenia: A Randomized Clinical Trial
Source: JAMA Netw Open. 2020 Dec 7;3(12):e2027909. doi: 10.1001/jamanetworkopen.2020.27909 (PMC12520709; doi:10.1001/jamanetworkopen.2020.27909)
Supplement: Supplement 2. — eAppendix 1. Sample Size Calculation eAppendix 2. CYP Guideline eAppendix 3. Analysis Notebook [file jamanetwopen-e2027909-s002.pdf]

Supplemental Online Content

Jürgens G, Andersen SE, Rasmussen HB, et al. Effect of routine cytochrome P450 2D6 and 2C19 genotyping on antipsychotic drug persistence in patients with schizophrenia: a randomized clinical trial. *JAMA Netw Open*. 2020;3(12):e2027909. doi:10.1001/jamanetworkopen.2020.27909

eAppendix 1. Sample Size Calculation

eAppendix 2. CYP Guideline

eAppendix 3. Analysis Notebook

This supplemental material has been provided by the authors to give readers additional information about their work.

© 2020 Jürgens G et al. *JAMA Network Open.*

eAppendix 1. Sample Size Calculation

We estimated the smallest relevant difference (d_0) to be 45 days with a standard deviation (σ) of 39 days (Lieberman JA et al. N Engl J Med. 2005;353(12):1209-1223). Seeking 5% and 10% risks of type I and II errors, and a balanced design, we computed the needed number of extreme metabolizers in each arm as

$$N_{\text{extreme,CTG}} = N_{\text{extreme,SCM}} = N_{\text{extreme,control}} = \frac{2\sigma^2(Z_\alpha + Z_\beta)^2}{d_0^2} = 19.5 \approx 20$$

Assuming a prevalence of 10%, approximately 600 patients had to be screened to identify 60 extreme metabolizers and 540 extensive or intermediate metabolizers for either CYP2D6 or CYP2C19.

© 2020 Jürgens G et al. *JAMA Network Open.*

eAppendix 2. CYP Guideline

Guideline
Genetic analysis of cytochrome P450 in clinical psychiatry

The Local Drug Committee

January 1, 2006

Table of Contents

Page 2:	**Introduction:**
	Which Patients?
	What is being analyzed?
	Who is using the analyzes?
Page 3:	**Test result:**
	The Genotype for each CYP enzyme.
	Clinical Prediction for each genotype.
	Clinical tutorial for each prediction.
Page 4:	**All genotypes:**
	CYP 2D6 Genotypes and Clinical Predictions.
	CYP 2C19 Genotypes and Clinical Predictions.
Page5:	**CYP 2D6 Tutorials:**
	Table 1. FAST (CYP 2D6)
	Table 2. NORMAL (CYP 2D6)
	Table 3. SLOW (CYP2D6)
Page 6:	**CYP2C19 Tutorials:**
	Table 4. NORMAL (CYP 2C19)
	Table 5. SLOW (CYP 2C19)

© 2020 Jürgens G et al. *JAMA Network Open.*

INTRODUCTION

Genetic tests of the cytochrome P450 system (CYP test) have been offered to patients admitted to Sct. Hans Hospital since August 2003.

Based on the experience gained from the pilot phase, the test is now offered to hospitals and general practitioners throughout the Capital Region of Denmark.

WHICH PATIENTS?

The Health Council for Psychiatry in the Capital Region of Denmark [H:S Sundhedsfaglig Råd for Psykiatri] concludes that there is not sufficient scientific evidence to recommend routine use of the CYP test in all psychiatric patients but recommends it´s use in the following situations:

1. In patients who have not previously been treated with drugs in order to avoid psychiatric drugs, that are metabolized slowly by the patient.

2. In patients suspected for lack of effect due to rapid metabolism.

3. In patients suspected for side effects or toxicity due to slow metabolism.

WHAT IS BEING ANALYZED?

The CYP test analyzes frequent functional variations in two genes with immediate relevance to the metabolism of antipsychotics and antidepressants, CYP2D6 and CYP2C19.

WHO IS USING THE ANALYZES?

The CYP test can be requested by physicians. Test results will be send to the treating physician.

© 2020 Jürgens G et al. *JAMA Network Open.*

TEST RESULTS:

The analytical results indicate both "genotype", "clinical preposition" and "guidance" for each of the 2 examined genes.

The specific genotype, e.g.

Genotype¤	Explanation	Number of normal§ genes
n/n	Two normal genes	Two normal genes
n/*3	One normal gene and one mutated gene (mutation No. 3)	One normal gene
*2/*2	Two mutated genes (mutation No. 2)	No normal genes
n/nx2	One normal and one duplicated gene	Three normal genes

¤ All possible genotypes are listed on page 4

§ "Normal gene" means that the mutations tested have not been found in the gene. However, the gene may contain rare mutations that are not tested for.

The corresponding clinical predicate

Clinical predicate	Number of normal genes
Normal	One or two normal genes
Slow	No normal genes
Fast	Three or more mutated genes

The corresponding clinical tutorial

Clinical predicate	CYP2D6	CYP2C19
Normal	See table 2	See table 4
Slow	See table 3	See table 5
Fast	See table 1	NA#

Mutations corresponding to fast metabolism have not been analyzed for CYP2C19

© 2020 Jürgens G et al. *JAMA Network Open.*

All GENOTYPES:

CYP2D6		
Genotype	Number of normal genes	Clinical predicate
nx2 / nx2<br>n / nx2	4+<br>3	**FAST metabolizer:**<br>CYP2D6 activity in individuals with 3 or more active 2D6 genes is likely to be significantly increased. Consequently, there will be an increased risk of suboptimal drug concentrations and lack of therapeutic effect.
n / n<br>nx2 / *3<br>nx2 / *4<br>nx2 / *5<br>nx2 / *6<br>nx2 / *4x2	2	**NORMAL metabolizer:**<br>CYP2D6 activity in individuals with two active 2D6 gene variants is expected to be normal.
n / *3<br>n / *4<br>n / *5<br>n / *6<br>n / *4x2	1	**NORMAL metabolizer:**<br>CYP2D6 activity in individuals with one active 2D6 gene variant is expected to be close to normal.
*3 / *3<br>*3 / *4<br>*3 / *5<br>*3 / *6<br>*4 / *4<br>*4 / *5<br>*4 / *6<br>*5 / *5<br>*5 / *6<br>*6 / *6<br>*3 / *4x2<br>*4 / *4x2<br>*5 / *4x2<br>*6 / *4x2<br>*4x2/ *4x2	0	**SLOW metabolizer:**<br>Individuals with no active 2D6 gene variants are not expected to have any CYP2D6 enzyme activity.<br>At standard dosage, there will be a risk of increased concentrations of 2D6-dependent drugs (see Table 1) and side effects.

© 2020 Jürgens G et al. *JAMA Network Open.*

CYP2C19		
Genotype	Number of normal genes	Clinical predicate
n / n	2	**NORMAL metabolizer:**<br>CYP2C19 activity in individuals with two active 2C19 gene variants is expected to be normal.
n / *2<br>n / *3	1<br>1	**NORMAL metabolizer:**<br>CYP2C19 activity in individuals with one active 2C19 gene variant is expected to be close to normal.
*2 / *2<br>*2 / *3<br>*3 / *3	0	**SLOW metabolizer:**<br>Individuals with no active 2C19 gene variants are not expected to have any CYP2C19 enzyme activity.<br>At standard dosage, there will be a risk of increased concentrations of 2C19-dependent drugs (see Table 1) and side effects.

CYP 2D6 Tutorials:

**Tabel 1. FAST (CYP2D6): Three or more normal genes**
INTERPRETATION: CYP2D6 activity is increased. Consequently, there is a risk of suboptimal drug concentrations and lack of therapeutic effect.<br>CONSIDERATIONS regarding use of antipsychotics and/or antidepressants:<br>1. Observation for lack of efficacy of CYP2D6-dependent drugs<br>2. Regular analysis of plasma drug concentration<br>3. Dose escalation of CYP2D6-dependent drugs above standard dose<br>4. Switch from CYP2D6-dependent to a non-independent drug

**Tabel 2. Normal (CYP2D6): One or two normal genes**
INTERPRETATION: Average CYP2D6-activity.<br>CONSIDERATIONS regarding use of antipsychotics and/or antidepressants:<br>1. Observation as usual<br>2. Use standard dose of CYP2D6-dependent drugs<br>3. No differences between CYP2D6-dependent and non-dependent drugs

**Tabel 3. SLOW (CYP2D6): No normal genes**
INTERPRETATION: No CYP2D6 activity. Consequently, there is a risk of elevated drug concentrations and drug related

© 2020 Jürgens G et al. *JAMA Network Open.*

side effects.

CONSIDERATIONS regarding use of antipsychotics and/or antidepressants:
1. Observation for side effects of CYP2D6-dependent drugs
2. Regular analysis of plasma drug concentration
3. Dose reduction of CYP2D6-dependent drugs below standard dose
4. Switch from CYP2D6-dependent to a non-independent drug

CYP 2C19 Tutorials:

Tabel 4. NORMAL (CYP2C19): One or two normal genes
INTERPRETATION: Average CYP2C19 activity.<br><br>CONSIDERATIONS regarding use of antidepressants<br>1. Observation as usual<br>2. Use standard dose of CYP2C19-dependent drugs<br>3. No differences between CYP2C19-dependent and non-dependent drugs

Tabel 5. Slow (CYP2C19): No normal genes.
INTERPRETATION: No CYP2C19 activity. Consequently, there is a risk of elevated drug concentrations and drug related side effects.
CONSIDERATIONS regarding use of antidepressants:<br>Observation for side effects of CYP2C19-dependent drugs<br>1. Regular analysis of plasma drug concentration<br>2. Dose reduction of CYP2C19-dependent drugs below standard dose<br>3. Switch from CYP2C19-dependent to a non-independent drug

© 2020 Jürgens G et al. *JAMA Network Open.*

Appendix to

Guideline
Genetic analysis of cytochrome P450 in clinical psychiatry

The Local Drug Committee

January 1, 2006

CYP2D6 ANALYSIS:

Tabel 1. CYP2D6 - dependent metabolism of selected drugs
Tabel 2. CYP2D6 - guided dosing with antidepressants
Tabel 3. CYP2D6 - interactions with selected drugs

CYP2C19 ANALYSIS:

Tabel 4. CYP2C19 - dependent metabolism of selected drugs
Tabel 5. CYP2C19 - guided dosing with antidepressants
Tabel 6. CYP2C19 - interactions with selected drugs

* The following document is an attempt to collect relevant pharmacological information that may be useful in translating the CYP test into a medical decision. The document does not replace official or binding guidelines.

Recommended sources:

- The Danish Medicines Agency's Summary of Product Characteristics, which for many drugs contains tutorials for the dosing of psychotropic drugs in patients with reduced CYP activity.
- The interaction database, which exists as a shortcut on all EPR computers, and on the Intranet at the top of the screen under LINKS.

If you have any questions, please contact the Drug information, Department of Clinical Pharmacology, Bispebjerg University Hospital, Copenhagen, telephone 3531 2332.

© 2020 Jürgens G et al. *JAMA Network Open.*

CYP 2D6 analysis

Tabel 1. CYP 2D6-dependent metabolism of selected drugs
Generic names in bold/blue. Trade names in italics. ATC code e.g.: N06

ANTIPSYCHOTICS N05A			ANTIDEPRESSANTS N06		
CYP 2D6 dependent	CYP 2D6 independent	CYP 2D6 unknown[a)]	CYP 2D6 dependent	CYP 2D6 independent	CYP 2D6 unknown[a)]
**aripiprazole**	**amisulprid**	**chlorprotixene**	**amitriptyline**	**bupropion**[d)]	**dosulepine**
Abilify	*Solian*	*Truxal*	*Amitriptylin*<br>*Saroten*	*Zyban*	*Prothiaden*
**fluphenazine**	**clozapine**	**flupentixol**	**clomipramine**	**citalopram**	
Siqualone	*Clozapin*<br>*Leponex*	*Fluanxol*	*Anafranil*<br>*Klomipramin*	*Akarin*<br>*Cipramil*<br>*Citadur*<br>*Citalopram*<br>*Citapram*	
**haloperidole**	**quetiapine**	**penfluridol**	**fluoxetine**	**escitalopram**	
Aloperidin<br>*Serenase*	*Seroquel*	*Semap*	*Afeksin*<br>*Fluoxetin*<br>*Flutin*<br>*Folizol*<br>*Fontex*	*Cipralex*	
**levomepromazine**	**ziprasidone**	**lithium**	**fluvoxamine**	**moclobemin**	
Nozinan	*Zeldox*	*Litarex*<br>*Litiumkarbonat*	*Fevarin*	*Aurorix*<br>*Moclomine*<br>*Moclastad*	
**melperon**			**imipramine**	**mirtazapine**	
Buronil			*Imipramin*	*Arintapin*<br>*Combar*<br>*Mirtazapin*<br>*Remeron*	
**olanzapine**[b)]			**mianserine**	**reboxetine**	
Zyprexa			*Mianserin*<br>*Tolmin*<br>*Tolvon*	*Edronax*	
**perphenazine**			**nortriptyline**	**sertraline**	
Trilafon			*Noritren*	*Sertralin*<br>*Zoloft*	
**pimozide**			**paroxetine**		
Orap			*Paroxetin*<br>*Seroxat*		
**risperidon**[c)]			**venlafaxine**		
Risperdal			*Dobupal*<br>*Efexor*<br>*Effexor*<br>*Vandral*		
**zuclopenthixol**					
Cisordinol					

[a)] "Unknown" means that it has not been possible to draw a final conclusion from the available data.
[b)] Olanzapine is primarily metabolized by CYP1A2 but depends to a lesser degree also on CYP2D6.
[c)] Risperidone is metabolized by CYP3A4 and CYP2D6. The latter metabolizes risperidone to an active metabolite (19-hydroxy-risperidone). CYP2D6 is expected to have a limited effect on the rate of metabolism of risperidone.
[d)] Bupropion is in DK only approved as "smoking cessation aid".

© 2020 Jürgens G et al. *JAMA Network Open.*

Tabel 2. CYP 2D6-adapted doses for antidepressants N06 a)
Generic names in bold/blue. Trade names in italics. ATC code e.g.: N06

**generisk navn** *Handelsnavne*	Starting dose (S) Maintenance dose (M)	Standard dose (mg) [b) e)]	Metabolic capacity [c)]	
			Normal	Slow
**amitriptyline** d) *Amitriptylin* *Saroten*	S<br>M	25-50<br>150-200	100%<br>100%	70%<br>50%
**clomipramine** d) *Anafranil* *Klomipramin*	S<br>M	25-50<br>75-150	100%<br>100%	60%<br>60%
**fluoxetine** *Afeksin* *Fluoxetin* *Flutin,* *Folizol* *Fontex*	S<br>M	20<br>---	100%<br>---	70%<br>---
**fluvoxamine** *Fevarin*	S<br>M	100-200<br>100-200	100%<br>100%	60%<br>90%
**imipramine** d) *Imipramin*	S<br>M	25-50<br>150-200	100%<br>100%	60%<br>30%
**mianserine** *Mianserin* *Tolmin* *Tolvon*	S<br>M	30<br>60-90	100%<br>100%	70%<br>70%
**nortriptyline** *Noritren*	S<br>M	25-50<br>100-150	100%<br>---	50%<br>50%
**paroxetine** *Paroxetin* *Seroxat*	S<br>M	20<br>20-50	100%<br>100%	20%<br>70%
**venlafaxine** *Dobupal* *Efexor* *Effexor* *Vandral*	S<br>M	---<br>150	---<br>100%	---<br>20%

a) Data based on Kircheiner et al. (Acta Psychiatr Scand 2001) and the Danish Pharmaceutical Catalog.
b) The term "standard dose" refers the normally recommended dose without regard to the CYP2D6 genotype. Note that the metabolism in the elderly is often decreased. Consequently, recommended starting and maintenance doses are lower.
c) There are No specific dose recommendations for FAST metabolism.
d) Approx. 1 out of 500 Europeans lack both functional CYP2D6 and CYP2C19 genes, both involved in the degradation of some antidepressants, including amitriptyline, clomipramine and imipramine. If these drugs are used in patients without CYP2D6 or CYP2C19 activity, the recommended dose reduction for both enzymes should be combined.
e) Values in brackets indicate the concentration range used for pharmacogenetic studies.

© 2020 Jürgens G et al. *JAMA Network Open.*

Tabel 3. CYP 2D6-interaktion for udvalgte præparater

Generic names in bold/blue. Trade names in *italics*. ATC code e.g.: N06

Inhibitors of the CYP-2D6 enzyme

Antipsychotics N05	Antidepressants N06	Abuse related drugs	Other drugs
**haloperidole**<br>*Aloperidin*<br>*Serenase*	**amitriptyline**<br>*Amitriptylin*<br>*Saroten*	**Kokain**	**amiodarone** C01<br>*Amiodaron*<br>*Cordarone*
**levomepromazine**<br>*Nozinan*	**bupropion** [d)]<br>*Zyban*	**methadon**	**buspiron** N05B<br>*Buspar*<br>*Buspiron*
**melperone**<br>*Buronil*	**citalopram**<br>*Akarin*<br>*Cipramil*<br>*Citadur*<br>*Citalopram*<br>*Citapram*	**Buprenorfine**<br>*Subutex*<br>*Temgesic*	**cimitidine** A02<br>*Acinil*<br>*Cimecodan*<br>*Cimetidin*<br>*Novamet*
**perphenazine**<br>*Trilafon*	**clomipramine**<br>*Anafranil*<br>*Klomipramin*		**propafenon** C01<br>*Rytmonorm*
**pimozide**<br>*Orap*	**escitalopram**<br>*Cipralex*		**ranitidine** A02<br>*Ranicodan*<br>*Ranitidin*<br>*Zantac*
	**fluoxetine**<br>*Afeksin*<br>*Fluoxetin*<br>*Flutin,*<br>*Folizol*<br>*Fontex*		**ritonavir** J05<br>*Kaletra*<br>*Norvir*
	**fluvoxamine**<br>*Fevarin*		**terbinafine** D01<br>*Lamicil*
	**moclobemid**<br>*Aurorix*<br>*Moclamine*<br>*Moclastad*		
	**nortriptyline**<br>*Noritren*		
	**paroxetine**<br>*Paroxetin*<br>*Seroxat*		
	**sertraline**<br>*Sertralin*<br>*Zoloft*		

d) Bupropion is in DK only approved as "smoking cessation aid".

© 2020 Jürgens G et al. *JAMA Network Open.*

CYP 2C19 analysis

Tabel 4. CYP 2C19-dependent metabolisme of selected drugs
Generic names in bold/blue. Trade names in *italics*. ATC code e.g.: N06

ANTIDEPRESSANTS N06			ANXIOLYTIKA N05B	Other drugs
CYP 2C19 dependent	CYP 2C19 independent	CYP 2C19 unknown (a)	CYP 2C19 dependent	CYP 2C19 dependent
**amitriptyline**<br>*Am*<br>*itriptylin*<br>*Saroten*	**fluoxetine**<br>*Afeksin*<br>*Fluoxetin*<br>*Flutin,*<br>*Folizol*<br>*Fontex*	**bupropion** (b)<br>*Zyban*	**diazepam**<br>*Apozepam*<br>*Diazepam*<br>*Hexalid*<br>*Stesolid*<br>*Valaxona*<br>*Valium*	**esomeprazole** A02<br>*Nexium*
**citalopram**<br>*Akarin*<br>*Cipramil*<br>*Citadur*<br>*Citalopram*<br>*Citapram*	**fluvoxamine**<br>*Fevarin*	**dosulepin**<br>Prothiaden	**flunitrazepam**<br>*Flunipam*<br>*Flunitrazepam*<br>*Ronal*	**omeprazole** A02<br>*Losec*<br>*Omeprazol*
**clomipramine**<br>*Anafranil*<br>*Klomipramin*	**mianserine**<br>*Mianserin*<br>*Tolmin*<br>*Tolvon*	**nortriptyline**<br>*Noritren*		**propranolol** C07<br>*Inderal*<br>*Propal*<br>*Propranolol*
**escitalopram**<br>*Cipralex*	**mirtazapine**<br>*Arintapin*<br>*Combar*<br>*Mirtazapin*<br>*Remeron*	**reboxetine**<br>*Edronax*		
**imipramine**<br>*Imipramin*	**paroxetine**<br>*Paroxetin*<br>*Seroxat*	**venlafaxine**<br>*Dobupal*<br>*Efexor*<br>*Effexor*<br>*Vandral*		
**moclobemide**<br>*Aurorix*<br>*Moclamine*<br>*Moclastad*	**sertraline**<br>*Sertralin*<br>*Zoloft*			

a) "Unknown" means that it has not been possible to draw a final conclusion from the available data.
b) Bupropion is in DK only approved as "smoking cessation aid".

© 2020 Jürgens G et al. *JAMA Network Open.*

Tabel 5. CYP 2C19-adaopted doses for antidepressants N06[a)] Generic names in bold/blue. Trade names in *italics*. ATC code e.g.: N06				
**Generic names** *Tradenames*	S = Starting dose<br>M = Maintenance dose	Standard dose (mg) [b) e)]	Metabolic capacity [c)]	
			Normal	Slow
**amitriptyline** [d)]<br>*Amitriptylin*<br>*Saroten*	S<br>M	---<br>150-200	---<br>110%	---<br>60%
**citalopram**<br>*Akarin*<br>*Cipramil*<br>*Citadur*<br>*Citalopram*<br>*Citapram*	S<br>M	---<br>20-40	---<br>100%	---<br>60%
**clomipramine** [d)]<br>*Anafranil*<br>*Klomipramin*	S<br>M	25-50<br>---	100%<br>---	70%<br>---
**imipramine** [d)]<br>*Imipramin*	S<br>M	25-50<br>150-200	100%<br>100%	70%<br>60%
**moclobemid**<br>*Aurorix*<br>*Moclamine*<br>*Moclastad*	S<br>M	300<br>300-600	110%<br>100%	40%<br>60%

a) Data based on Kircheiner et al. (Acta Psychiatr Scand 2001) and the Danish Pharmaceutical Catalog.
b) The term "standard dose" refers the normally recommended dose without regard to the CYP2D6 genotype. Note that the metabolism in the elderly is often decreased. Consequently, recommended starting and maintenance doses are lower.
c) There are No specific dose recommendations for FAST metabolism.
d) Approx. 1 out of 500 Europeans lack both functional CYP2D6 and CYP2C19 genes, both involved in the degradation of some antidepressants, including amitriptyline, clomipramine and imipramine. If these drugs are used in patients without CYP2D6 or CYP2C19 activity, the recommended dose reduction for both enzymes should be combined.
e) Values in brackets indicate the concentration range used for pharmacogenetic studies.

© 2020 Jürgens G et al. *JAMA Network Open.*

Tabel 6. CYP 2C19-interactions for selected drugs
Generic names in bold/blue. Trade names in *italics*. ATC code e.g.: N06

CYP2C19 enzyme inhibitor		CYP2C19 enzyme inducer
**Antidepressants N06**	**Other drugs**	
**fluoxetine**<br>*Afeksin*<br>*Fluoxetin*<br>*Flutin,*<br>*Folizol*<br>*Fontex*	**cimitidine** A02<br>*Acinil*<br>*Cimecodan*<br>*Cimetidin*<br>*Novamet*	**carbamazepine** N03<br>*Karbamazepin*<br>*Tegretol*<br>*Trimonil*
**fluvoxamine**<br>*Fevarin*	**esomeprazole** A02<br>*Nexium*	**phenytoine** N03<br>*Fenytoin*
**moclobemide**<br>*Aurorix*<br>*Moclamine*<br>*Moclastad*	**lansoprazole** A02<br>*Lanzo*	**prednisone** H02<br>*Prednison*
**paroxetine**<br>*Paroxetin*<br>*Seroxat*	**omeprazole** A02<br>*Losec*<br>*Omeprazol*	
**sertraline**<br>*Sertralin*<br>*Zoloft*	**Oral contraceptives**<br>*Miscellanouse*	
	**oxcarbazepine** N03<br>*Apydan*<br>*Trileptal*	
	**topiramate** N03<br>*Epitomax*<br>*Topamac*<br>*Topimax*	
	**acetylsalicylic acid** N02<br>*Asperin*<br>*Idotyl*<br>*Kodimagnyl*<br>*Magnyl*<br>*Treo*	

© 2020 Jürgens G et al. *JAMA Network Open.*

eAppendix 3. Analysis Notebook

Basics

Load the R packages we need for our analyses and set some useful defaults.

```
packages <- c("mice", "VIM", "car", "descr", "MASS", "survival", "survminer", "forcats",
              "tidyr", "dplyr", "gridExtra", "broom", "knitr", "tableone", "tibble")
for (p in packages) library(p, character.only = TRUE)

# For pretty outputs
pretty <- c(
  # UKU scores
  all = "All", aut = "Autonomic", neu = "Neurologic", oth = "Other organ systems",
  psy = "Psychiatric",
  # Groups
  rcm = "Control", ctl = "Control", ctg = "CYP test-guided (CTG)",
  scm = "Structured clinical monitoring (SCM)",
  # SAPS
  hallucinations = "Hallucinations", delusions = "Delusions",
  # Variables in models
  group = "Study arm", groupctg = "Group: CTG (vs. control)",
  groupscm = "Group: SCM (vs. control)", gender = "Gender", gendermale = "Male gender",
  age = "Age", bl_uku = "UKU score at baseline",
  "(Intercept)" = "(Intercept)", saeiyes = "Special Assertive Early Intervention",
  illness_duration = "Duration of illness",
  extr_metabolizeryes = "Extreme metabolizer",
  extr_metabolizer = "Extreme metabolizer", "X.Intercept." = "(Intercept)",
  cyp2d6_dep_drugTRUE = "CYP2D6-dependent drug use",
  cyp2d6_dep_drug = "CYP2D6-dependent drug use",
  cyp2c19_dep_drugTRUE = "CYP2C19-dependent drug use",
  cyp2c19_dep_drug = "CYP2C19-dependent drug use",
  bl_hallucinations_num = "Hallucinations at baseline, numeric",
  bl_delusions_num = "Delusions at baseline, numeric",
  poor_metabolizeryes = "Poor metabolizer",
  fast_metabolizeryes = "Fast metabolizer",
  "gendermale:poor_metabolizeryes" = "Poor metabolizer + male",
  "gendermale:fast_metabolizeryes" = "Fast metabolizer + male")

# Output options
knitr::opts_chunk$set(echo = TRUE, fig.align = "center", fig.width = 8, fig.height = 5,
                      warning = FALSE, messsage = FALSE)
options(width = 800, warning = FALSE) # avoid wrapped output, ignore irrelevant warnings
text_size <- 9 # for plots
geom_text_size <- text_size / ggplot2::.pt
  # see https://stackoverflow.com/questions/17311917/ggplot2-the-unit-of-size
default_theme <- theme_linedraw() + # set default ggplot theme for unified visualisations
    theme(panel.border = element_blank(),
          panel.background = element_blank(),
          panel.grid.major = element_line(colour = "grey92", size = 0.3),
          panel.grid.minor = element_blank(),
          axis.line = element_blank(),
          axis.ticks = element_blank(),
          axis.text.y = element_text(colour = "#000000", size = text_size),
          axis.text.x = element_text(colour = "#000000", size = text_size),
          strip.background = element_rect(fill = grey(0.9), size = 0),
          strip.text = element_text(colour = "black"),
          text = element_text(size = text_size),
```

© 2020 Jürgens G et al. *JAMA Network Open.*

```
          legend.title = element_blank(),
          plot.title = element_text(size = text_size * 1.2))
theme_set(default_theme)
```

Utility functions

We define some utility functions here for simpler subsequent code.

```
# Show cross-tabulations with harmonised format
ct <- function(v1, v2, dnn = c("", ""), prop.r = TRUE, prop.c = TRUE)
  CrossTable(v1, v2, prop.r = prop.r, prop.c = prop.c, prop.t = FALSE, dnn = dnn,
             prop.chisq = FALSE, missing.include = TRUE, format = "SPSS",
             digits = list(others = 3, percent = 0), cell.layout = FALSE)

# Make first letter upper-case
first_up <- function(x) { # x: string
  x <- unlist(strsplit(x, ""))
  paste0(toupper(x[1]), paste0(x[-1], collapse = ""))
}

# Compute pseudo-R^2 values for logistic regressions
logistic_pseudo_R2s <- function(mod) { # mod: glm model object
  # Adapted from Field, 2012: Discovering Statistics Using R [p. 334]
  dev <- mod$deviance
  null_dev <- mod$null.deviance
  n <- length(mod$fitted.values)
  R_l <- 1 - dev / null_dev
  R_cs <- 1 - exp(-(null_dev - dev) / n)
  R_n <-  R_cs / (1 - exp(-null_dev / n))
  cat("\n> Pseudo R^2 for logistic regression:\n")
  cat("Hosmer and Lemeshow  ", round(R_l, 3), "\n")
  cat("Cox and Snell        ", round(R_cs, 3), "\n")
  cat("Nagelkerke           ", round(R_n, 3), "\n")
}

# Standard ggplot objects with nicer colouring and filling palettes
ggplot1 <- function(...)
  ggplot(...) + scale_fill_brewer(palette = "Set1") + scale_color_brewer(palette = "Set1")

ggplot2 <- function(...)
  ggplot(...) + scale_fill_brewer(palette = "Set2") + scale_color_brewer(palette = "Set2")

# Simplify code when tweaking KM plots
plot_theme <- function(scale = 1, axes = TRUE) {
  text_size <- text_size * scale
  t <- default_theme +
    theme(axis.text.y = element_text(colour = "#000000", size = text_size),
        axis.text.x = element_text(colour = "#000000", size = text_size),
        plot.title = element_text(size = text_size * 1.2))
  if (isTRUE(axes)) {
    t + theme(axis.title.y = element_text(size = text_size),
              axis.title.x = element_text(size = text_size))
  } else {
    t + theme(axis.title = element_blank())
  }
}

# Gives tidy parameter estimates with 95% CIs
```

```
tidy_res <- function(m, est = "estimate", dec = 2, nice = TRUE) {
  trans <- switch(est, OR = exp, NULL)
  x <- bind_cols(select(tidy(m), !!est := estimate), confint_tidy(m))
  if (!is.null(trans)) x <- mutate_all(x, trans)
  x <- mutate_all(x, ~ ifelse(abs(.) > 1000, NA_integer_, .)) %>%
    mutate_all(~ sprintf("%.2f", .))
  o <- bind_cols(select(tidy(m), term), x)
  if (nice) mutate(o, term = pretty[term]) else o # return human-friendly param names?
}
```

Data import and processing

We've preprocessed the data into an operational format, and load them into memory here. The data are kept in the df object. To showcase the data structure, we simulate 4 patients from our data via permutation, so we don't share any data from specific patients. Note that, due to the simple permutation procedure, some of the combined or derived variables will not have meaningful values, but given the illustrative purpose we settled for this less involved procedure.

```
load("processed_data.RData")
t(apply(df[complete.cases(df), -1], 2, sample, size = 4, replace = TRUE)) %>%
  as.data.frame() %>%
  setNames(paste0("sim_", 1:4)) %>%
  kable(align = "c")
```

	sim_1	sim_2	sim_3	sim_4
group	ctl	ctl	scm	scm
persistence	112	365	365	365
event	1	1	0	1
age	50.24931	38.40548	48.15068	48.21096
gender	female	male	male	male
illness_duration	0.73	17.48	16.42	1.33
extr_metabolizer	no	no	no	no
poor_metabolizer	yes	no	no	no
fast_metabolizer	no	no	no	no
saei	no	yes	no	no
icd_major	F20	F20	F20	F21
cyp2c19_pheno	EM	IM	EM	IM
cyp2d6_pheno	IM	EM	IM	EM
cyp2d6_dep_drug	TRUE	TRUE	TRUE	FALSE
cyp2c19_dep_drug	FALSE	TRUE	TRUE	FALSE
cyp_dep_drug	FALSE	TRUE	TRUE	TRUE
bl_uku	20	37	28	31
fu_uku	14.0	14.0	7.0	18.5
uku_oth	-5	2	1	1
uku_neu	-2	1	0	1
uku_aut	-1	1	-2	-2
uku_psy	2	0	1	1

© 2020 Jürgens G et al. *JAMA Network Open.*

uku_all	-10	-8	6	6
bl_hallucinations	0	2	0	0
fu_hallucinations	3	3	1	2
hallucinations	0	-4	0	0
hallucinations_bin	improved	not improved	not improved	not improved
bl_delusions	0	2	4	2
fu_delusions	3	4	0	0
delusions	-1	3	0	0
delusions_bin	not improved	not improved	not improved	not improved
n_shifts_all	3	2	1	0
n_drug_shifts	1	0	0	0
bin_drug_shifts	0	1	0	0
retained	yes	yes	yes	yes
retained_group	yes_ctg	yes_scm	yes_scm	yes_scm

Missing data

Here, we look at the missing-data pattern. 1's mean observed value, 0's mean missing value. Column titles indicate the number of patients with the pattern in that column. The bottom row shows the total number of variables with missing values in that column. The right-most column shows the number of patients with missing values for each variable.

```
kable(t(md.pattern(filter(df, !is.na(event)), plot = FALSE)), align = "c")
```

	175	2	2	1	1	39	30	1	1	13	5	1	3	1	2	2	4	1	1	1	4	
id	1	1	1	1	1	1	1	1	1	1	1	1	1	1	1	1	1	1	1	1	1	0
group	1	1	1	1	1	1	1	1	1	1	1	1	1	1	1	1	1	1	1	1	1	0
persistence	1	1	1	1	1	1	1	1	1	1	1	1	1	1	1	1	1	1	1	1	1	0
event	1	1	1	1	1	1	1	1	1	1	1	1	1	1	1	1	1	1	1	1	1	0
age	1	1	1	1	1	1	1	1	1	1	1	1	1	1	1	1	1	1	1	1	1	0
gender	1	1	1	1	1	1	1	1	1	1	1	1	1	1	1	1	1	1	1	1	1	0
extr_metabolizer	1	1	1	1	1	1	1	1	1	1	1	1	1	1	1	1	1	1	1	1	1	0
poor_metabolizer	1	1	1	1	1	1	1	1	1	1	1	1	1	1	1	1	1	1	1	1	1	0
fast_metabolizer	1	1	1	1	1	1	1	1	1	1	1	1	1	1	1	1	1	1	1	1	1	0
saei	1	1	1	1	1	1	1	1	1	1	1	1	1	1	1	1	1	1	1	1	1	0
cyp2c19_pheno	1	1	1	1	1	1	1	1	1	1	1	1	1	1	1	1	1	1	1	1	1	0
cyp2d6_pheno	1	1	1	1	1	1	1	1	1	1	1	1	1	1	1	1	1	1	1	1	1	0
cyp2d6_dep_drug	1	1	1	1	1	1	1	1	1	1	1	1	1	1	1	1	1	1	1	1	1	0
cyp2c19_dep_drug	1	1	1	1	1	1	1	1	1	1	1	1	1	1	1	1	1	1	1	1	1	0

© 2020 Jürgens G et al. *JAMA Network Open.*

cyp_dep_drug	1	1	1	1	1	1	1	1	1	1	1	1	1	1	1	1	1	1	1	1	1	0
n_shifts_all	1	1	1	1	1	1	1	1	1	1	1	1	1	1	1	1	1	1	1	1	1	0
n_drug_shifts	1	1	1	1	1	1	1	1	1	1	1	1	1	1	1	1	1	1	1	1	1	0
bin_drug_shifts	1	1	1	1	1	1	1	1	1	1	1	1	1	1	1	1	1	1	1	1	1	0
retained	1	1	1	1	1	1	1	1	1	1	1	1	1	1	1	1	1	1	1	1	1	0
retained_group	1	1	1	1	1	1	1	1	1	1	1	1	1	1	1	1	1	1	1	1	1	0
icd_major	1	1	1	1	1	1	1	1	1	1	1	1	1	1	1	1	1	1	0	0	0	6
bl_delusions	1	1	1	1	1	1	1	1	1	1	1	1	1	1	0	0	0	0	1	0	0	14
bl_hallucinations	1	1	1	1	1	1	1	1	1	1	1	1	0	0	0	0	0	0	1	0	0	18
illness_duration	1	1	1	1	1	1	1	1	1	0	0	0	1	1	1	1	0	0	0	0	0	30
bl_uku	1	1	1	1	1	1	0	0	0	1	1	0	1	1	1	0	0	0	1	0	0	45
fu_hallucinations	1	1	1	1	1	0	1	1	0	1	0	1	1	1	1	0	1	0	1	1	0	52
fu_delusions	1	1	1	1	0	0	1	1	0	1	0	1	1	1	1	0	1	0	1	1	0	53
uku_neu	1	1	1	0	1	0	1	1	0	1	0	0	1	1	1	0	0	0	1	0	0	59
uku_aut	1	1	1	0	1	0	1	1	0	1	0	0	1	1	1	0	0	0	1	0	0	59
uku_psy	1	1	1	0	1	0	1	1	0	1	0	0	1	0	1	0	0	0	1	0	0	60
delusions	1	1	1	1	0	0	1	1	0	1	0	1	1	1	0	0	0	0	1	0	0	60
delusions_bin	1	1	1	1	0	0	1	1	0	1	0	1	1	1	0	0	0	0	1	0	0	60
fu_uku	1	0	0	0	1	0	1	0	0	1	0	0	1	0	1	0	1	0	1	0	0	61
uku_oth	1	1	0	0	1	0	1	1	0	1	0	0	1	1	1	0	0	0	1	0	0	61
uku_all	1	1	0	0	1	0	1	1	0	1	0	0	1	0	1	0	0	0	1	0	0	62
hallucinations	1	1	1	1	1	0	1	1	0	1	0	1	0	0	0	0	0	0	1	0	0	63
hallucinations_bin	1	1	1	1	1	0	1	1	0	1	0	1	0	0	0	0	0	0	1	0	0	63
	0	1	3	6	3	12	1	2	13	1	13	8	3	6	6	15	13	16	2	15	17	826

Patient with follow-up data for neither SAPS nor UKU

```
filter(df, retained == "yes") %>%
  transmute(group,
            x = is.na(fu_uku) & is.na(fu_hallucinations) & is.na(fu_delusions)) %>%
  group_by(group) %>%
  summarise(n = sum(x), prop = mean(x), .groups = "drop") %>%
  bind_rows(tibble(group = "overall", n = sum(.$n), prop = n / 290))

## # A tibble: 4 x 3
##   group       n  prop
##   <chr>   <int> <dbl>
## 1 ctl        25 0.248
## 2 ctg        13 0.137
## 3 scm        14 0.149
## 4 overall    52 0.179
```

© 2020 Jürgens G et al. *JAMA Network Open.*

eTable 1: Patient characteristics

Summary statistics for non-retained subjects are left out to avoid privacy issues due to data sparsity.

```
table1_df <- select(df, -id, -retained_group) %>%
  mutate(n_shifts_all_binned = ifelse(n_shifts_all < 4, n_shifts_all, "4+")) %>%
  select(-n_shifts_all)
cat_vars <- names(select(table1_df, -group, -persistence, -age, -illness_duration, -bl_uku,
                         -starts_with("uku_")))

CreateTableOne(strata = "group",
               factorVars = cat_vars,
               includeNA = TRUE,
               test = FALSE,
               data = filter(table1_df, retained == "yes")) %>%
  summary()

## 
##      ### Summary of continuous variables ###
## 
## group: ctl
##                    n miss p.miss  mean  sd median p25 p75   min max skew kurt
## persistence      101    0      0 204.5 150    206  49 365   1.0 365 -0.1 -1.8
## age              101    0      0  42.1  13     42  31  53  20.1  73  0.2 -0.8
## illness_duration 101    9      9   9.7   9      7   3  13   0.2  38  1.3  1.2
## bl_uku           101   14     14  18.3   9     18  13  24   0.0  46  0.4  0.6
## uku_oth          101   27     27  -0.6   3      0  -3   1  -8.0   6 -0.2 -0.4
## uku_neu          101   26     26   0.5   2      1  -1   2  -7.0   5 -0.4  0.9
## uku_aut          101   26     26  -0.5   3      0  -2   1 -11.0   8 -0.2  1.3
## uku_psy          101   26     26   0.2   4      0  -2   3 -13.0   9 -0.5  0.8
## uku_all          101   27     27  -0.4   9      0  -5   5 -26.0  22 -0.2  1.0
## -----------------------------------------------------------------------------------------------------
-----------------------------------------------------------------------------------------------------
-----------------------------------------------------------------------------------------------------
-----------------------------------------------------------------------------------------------------
-----------------------------------------------------------------------------------------------------
-----------------------------------------------------------------------------------------------------
---
## group: ctg
##                   n miss p.miss   mean  sd median p25 p75   min max  skew kurt
## persistence      95    0      0 200.22 148    170  52 365   4.0 365  0.02 -1.8
## age              95    0      0  41.57  13     42  32  51  19.0  68  0.05 -1.0
## illness_duration 95   10     11   9.76   9      7   3  15   0.2  50  1.64  3.8
## bl_uku           95   17     18  20.10   9     19  13  26   5.0  39  0.37 -0.8
## uku_oth          95   18     19  -0.38   3      0  -2   1 -13.0   9 -0.48  4.2
## uku_neu          95   17     18   0.03   2      0  -1   1  -6.0   6 -0.32  0.6
## uku_aut          95   17     18  -0.37   3     -1  -2   1  -6.0   9  0.74  0.8
## uku_psy          95   17     18   0.38   5      1  -2   3 -24.0   9 -1.48  6.4
## uku_all          95   18     19  -0.26   9      2  -4   5 -29.0  19 -0.87  1.8
## -----------------------------------------------------------------------------------------------------
-----------------------------------------------------------------------------------------------------
-----------------------------------------------------------------------------------------------------
-----------------------------------------------------------------------------------------------------
-----------------------------------------------------------------------------------------------------
-----------------------------------------------------------------------------------------------------
---
## group: scm
##                   n miss p.miss  mean  sd median p25 p75   min max skew kurt
```

© 2020 Jürgens G et al. *JAMA Network Open.*

```
## persistence      94    0      0 223.8 141  252.5  79 365   2.0 365 -0.3 -1.6
## age              94    0      0  40.5  12   40.2  30  48  18.7  71  0.3 -0.7
## illness_duration 94   11     12   9.6   9    6.2   3  16   0.2  40  1.3  0.8
## bl_uku           94   14     15  20.4  10   19.0  13  27   0.0  47  0.4 -0.2
## uku_oth          94   16     17  -0.7   4   -0.5  -2   2 -17.0   8 -0.9  3.2
## uku_neu          94   16     17   0.8   2    1.0  -1   2  -3.0   7  0.3  0.2
## uku_aut          94   16     17   0.1   3    0.0  -1   1  -8.0   9  0.2  1.6
## uku_psy          94   17     18   0.7   4    0.0  -2   4  -9.0  10  0.1 -0.2
## uku_all          94   17     18   0.9   8    0.0  -3   5 -15.0  24  0.5  0.3
##
## Standardize mean differences
##                     average     1 vs 2      1 vs 3     2 vs 3
## persistence      0.10814779 0.02879184 0.132510341 0.16314119
## age              0.08737603 0.04462711 0.131138840 0.08636213
## illness_duration 0.01118806 0.01007771 0.006857507 0.01662895
## bl_uku           0.14831095 0.20011251 0.218163635 0.02665671
## uku_oth          0.05605461 0.07145461 0.016915527 0.07979368
## uku_neu          0.23358541 0.21645415 0.133660798 0.35064127
## uku_aut          0.12890409 0.03423616 0.195227505 0.15724861
## uku_psy          0.07226314 0.03757728 0.114532676 0.06467947
## uku_all          0.10481860 0.01071592 0.157376881 0.14636300
##
## =====================================================================================
##
##      ### Summary of categorical variables ###
##
## group: ctl
##                 var   n miss p.miss        level freq percent cum.percent
##               group 101    0    0.0          ctl  101   100.0       100.0
##                                              ctg    0     0.0       100.0
##                                              scm    0     0.0       100.0
##
##               event 101    0    0.0            0   38    37.6        37.6
##                                                1   63    62.4       100.0
##
##              gender 101    0    0.0       female   46    45.5        45.5
##                                             male   55    54.5       100.0
##
##     extr_metabolizer 101    0    0.0           no   80    79.2        79.2
##                                              yes   21    20.8       100.0
##
##     poor_metabolizer 101    0    0.0           no   83    82.2        82.2
##                                              yes   18    17.8       100.0
##
##     fast_metabolizer 101    0    0.0           no   98    97.0        97.0
##                                              yes    3     3.0       100.0
##
##                saei 101    0    0.0           no   86    85.1        85.1
##                                              yes   15    14.9       100.0
##
##           icd_major 101    0    0.0          F20   66    65.3        65.3
##                                              F21   20    19.8        85.1
##                                              F22    2     2.0        87.1
##                                              F23    2     2.0        89.1
##                                              F25    8     7.9        97.0
##                                              F28    0     0.0        97.0
##                                              F29    0     0.0        97.0
##                                             <NA>    3     3.0       100.0
##
```

© 2020 Jürgens G et al. *JAMA Network Open.*

```
##        cyp2c19_pheno 101    0    0.0            EM   78    77.2         77.2
##                                                 IM   20    19.8         97.0
##                                                 PM    3     3.0        100.0
##
##         cyp2d6_pheno 101    0    0.0            EM   50    49.5         49.5
##                                                 IM   33    32.7         82.2
##                                                 PM   15    14.9         97.0
##                                                 UM    3     3.0        100.0
##
##      cyp2d6_dep_drug 101    0    0.0         FALSE   22    21.8         21.8
##                                               TRUE   79    78.2        100.0
##
##     cyp2c19_dep_drug 101    0    0.0         FALSE   76    75.2         75.2
##                                               TRUE   25    24.8        100.0
##
##         cyp_dep_drug 101    0    0.0         FALSE   18    17.8         17.8
##                                               TRUE   83    82.2        100.0
##
##               fu_uku 101    0    0.0             0    3     3.0          3.0
##                                                  1    0     0.0          3.0
##                                                  2    0     0.0          3.0
##                                                2.5    0     0.0          3.0
##                                                  3    0     0.0          3.0
##                                                  4    1     1.0          4.0
##                                                  5    1     1.0          5.0
##                                                  6    2     2.0          6.9
##                                                  7    2     2.0          8.9
##                                                  8    2     2.0         10.9
##                                                8.5    0     0.0         10.9
##                                                  9    1     1.0         11.9
##                                                 10    1     1.0         12.9
##                                                 11    2     2.0         14.9
##                                                 12    0     0.0         14.9
##                                                 13    5     5.0         19.8
##                                                 14    4     4.0         23.8
##                                                 15    4     4.0         27.7
##                                                 16    4     4.0         31.7
##                                                 17    2     2.0         33.7
##                                               17.5    1     1.0         34.7
##                                                 18    2     2.0         36.6
##                                               18.5    0     0.0         36.6
##                                                 19    4     4.0         40.6
##                                               19.5    1     1.0         41.6
##                                                 20    3     3.0         44.6
##                                                 21    2     2.0         46.5
##                                                 22    1     1.0         47.5
##                                                 23    1     1.0         48.5
##                                                 24    2     2.0         50.5
##                                                 25    2     2.0         52.5
##                                               25.5    0     0.0         52.5
##                                                 26    2     2.0         54.5
##                                               26.5    0     0.0         54.5
##                                                 27    0     0.0         54.5
##                                                 28    3     3.0         57.4
##                                                 29    1     1.0         58.4
##                                                 30    0     0.0         58.4
##                                                 31    3     3.0         61.4
##                                                 32    1     1.0         62.4
##                                                 33    0     0.0         62.4
```

© 2020 Jürgens G et al. *JAMA Network Open.*

```
##                                               34   0     0.0        62.4
##                                               35   4     4.0        66.3
##                                               36   1     1.0        67.3
##                                               37   0     0.0        67.3
##                                               38   0     0.0        67.3
##                                               39   0     0.0        67.3
##                                               40   0     0.0        67.3
##                                               41   2     2.0        69.3
##                                               42   0     0.0        69.3
##                                               44   1     1.0        70.3
##                                               45   0     0.0        70.3
##                                               49   0     0.0        70.3
##                                             49.5   0     0.0        70.3
##                                               50   0     0.0        70.3
##                                               51   0     0.0        70.3
##                                               53   1     1.0        71.3
##                                               56   1     1.0        72.3
##                                             <NA>  28    27.7       100.0
##
##    bl_hallucinations 101    0    0.0            0  57    56.4        56.4
##                                                1   6     5.9        62.4
##                                                2  12    11.9        74.3
##                                                3  11    10.9        85.1
##                                                4  11    10.9        96.0
##                                             <NA>   4     4.0       100.0
##
##    fu_hallucinations 101    0    0.0            0  46    45.5        45.5
##                                                1   0     0.0        45.5
##                                                2   8     7.9        53.5
##                                                3  10     9.9        63.4
##                                                4  12    11.9        75.2
##                                             <NA>  25    24.8       100.0
##
##       hallucinations 101    0    0.0           -4   4     4.0         4.0
##                                               -3   6     5.9         9.9
##                                               -2   5     5.0        14.9
##                                               -1   5     5.0        19.8
##                                                0  38    37.6        57.4
##                                                1   7     6.9        64.4
##                                                2   6     5.9        70.3
##                                                3   3     3.0        73.3
##                                                4   1     1.0        74.3
##                                             <NA>  26    25.7       100.0
##
##   hallucinations_bin 101    0    0.0 not improved  58    57.4        57.4
##                                         improved  17    16.8        74.3
##                                             <NA>  26    25.7       100.0
##
##         bl_delusions 101    0    0.0            0  37    36.6        36.6
##                                                1  11    10.9        47.5
##                                                2  17    16.8        64.4
##                                                3  14    13.9        78.2
##                                                4  18    17.8        96.0
##                                             <NA>   4     4.0       100.0
##
##         fu_delusions 101    0    0.0            0  30    29.7        29.7
##                                                1   8     7.9        37.6
##                                                2  17    16.8        54.5
##                                                3   7     6.9        61.4
```

© 2020 Jürgens G et al. *JAMA Network Open.*

```
##                                                   4   14    13.9        75.2
##                                                <NA>   25    24.8       100.0
##
##            delusions 101    0    0.0             -4    1     1.0         1.0
##                                                  -3    3     3.0         4.0
##                                                  -2    5     5.0         8.9
##                                                  -1   11    10.9        19.8
##                                                   0   38    37.6        57.4
##                                                   1    6     5.9        63.4
##                                                   2    8     7.9        71.3
##                                                   3    2     2.0        73.3
##                                                   4    1     1.0        74.3
##                                                <NA>   26    25.7       100.0
##
##        delusions_bin 101    0    0.0 not improved   58    57.4        57.4
##                                            improved   17    16.8        74.3
##                                                <NA>   26    25.7       100.0
##
##        n_drug_shifts 101    0    0.0              0   87    86.1        86.1
##                                                   1   11    10.9        97.0
##                                                   2    3     3.0       100.0
##                                                   3    0     0.0       100.0
##
##      bin_drug_shifts 101    0    0.0              0   87    86.1        86.1
##                                                   1   14    13.9       100.0
##
##             retained 101    0    0.0            yes  101   100.0       100.0
##
##  n_shifts_all_binned 101    0    0.0              0   37    36.6        36.6
##                                                   1   17    16.8        53.5
##                                                   2   16    15.8        69.3
##                                                   3    8     7.9        77.2
##                                                  4+   23    22.8       100.0
##
## ---------------------------------------------------------------------------------------------
---------------------------------------------------------------------------------------------
---------------------------------------------------------------------------------------------
---------------------------------------------------------------------------------------------
---------------------------------------------------------------------------------------------
---------------------------------------------------------------------------------------------
---
## group: ctg
##                  var  n miss p.miss        level freq percent cum.percent
##                group 95    0    0.0          ctl    0     0.0         0.0
##                                              ctg   95   100.0       100.0
##                                              scm    0     0.0       100.0
##
##                event 95    0    0.0            0   35    36.8        36.8
##                                                1   60    63.2       100.0
##
##               gender 95    0    0.0       female   43    45.3        45.3
##                                             male   52    54.7       100.0
##
##     extr_metabolizer 95    0    0.0           no   75    78.9        78.9
##                                              yes   20    21.1       100.0
##
##     poor_metabolizer 95    0    0.0           no   80    84.2        84.2
##                                              yes   15    15.8       100.0
##
```

© 2020 Jürgens G et al. *JAMA Network Open.*

```
##     fast_metabolizer 95    0    0.0            no   90    94.7        94.7
##                                               yes    5     5.3       100.0
##
##                 saei 95    0    0.0            no   80    84.2        84.2
##                                               yes   15    15.8       100.0
##
##            icd_major 95    0    0.0           F20   68    71.6        71.6
##                                               F21   19    20.0        91.6
##                                               F22    3     3.2        94.7
##                                               F23    1     1.1        95.8
##                                               F25    2     2.1        97.9
##                                               F28    0     0.0        97.9
##                                               F29    0     0.0        97.9
##                                              <NA>    2     2.1       100.0
##
##        cyp2c19_pheno 95    0    0.0            EM   68    71.6        71.6
##                                                IM   25    26.3        97.9
##                                                PM    2     2.1       100.0
##
##         cyp2d6_pheno 95    0    0.0            EM   47    49.5        49.5
##                                                IM   30    31.6        81.1
##                                                PM   13    13.7        94.7
##                                                UM    5     5.3       100.0
##
##      cyp2d6_dep_drug 95    0    0.0         FALSE   18    18.9        18.9
##                                              TRUE   77    81.1       100.0
##
##     cyp2c19_dep_drug 95    0    0.0         FALSE   77    81.1        81.1
##                                              TRUE   18    18.9       100.0
##
##         cyp_dep_drug 95    0    0.0         FALSE   15    15.8        15.8
##                                              TRUE   80    84.2       100.0
##
##               fu_uku 95    0    0.0             0    1     1.1         1.1
##                                                 1    1     1.1         2.1
##                                                 2    0     0.0         2.1
##                                               2.5    1     1.1         3.2
##                                                 3    2     2.1         5.3
##                                                 4    2     2.1         7.4
##                                                 5    2     2.1         9.5
##                                                 6    1     1.1        10.5
##                                                 7    2     2.1        12.6
##                                                 8    0     0.0        12.6
##                                               8.5    0     0.0        12.6
##                                                 9    0     0.0        12.6
##                                                10    3     3.2        15.8
##                                                11    2     2.1        17.9
##                                                12    2     2.1        20.0
##                                                13    6     6.3        26.3
##                                                14    3     3.2        29.5
##                                                15    1     1.1        30.5
##                                                16    2     2.1        32.6
##                                                17    3     3.2        35.8
##                                              17.5    0     0.0        35.8
##                                                18    3     3.2        38.9
##                                              18.5    1     1.1        40.0
##                                                19    1     1.1        41.1
##                                              19.5    0     0.0        41.1
##                                                20    2     2.1        43.2
```

© 2020 Jürgens G et al. *JAMA Network Open.*

```
##                                          21    4     4.2        47.4
##                                          22    3     3.2        50.5
##                                          23    1     1.1        51.6
##                                          24    1     1.1        52.6
##                                          25    2     2.1        54.7
##                                        25.5    1     1.1        55.8
##                                          26    0     0.0        55.8
##                                        26.5    0     0.0        55.8
##                                          27    2     2.1        57.9
##                                          28    5     5.3        63.2
##                                          29    1     1.1        64.2
##                                          30    2     2.1        66.3
##                                          31    3     3.2        69.5
##                                          32    1     1.1        70.5
##                                          33    1     1.1        71.6
##                                          34    0     0.0        71.6
##                                          35    0     0.0        71.6
##                                          36    1     1.1        72.6
##                                          37    1     1.1        73.7
##                                          38    2     2.1        75.8
##                                          39    0     0.0        75.8
##                                          40    0     0.0        75.8
##                                          41    0     0.0        75.8
##                                          42    0     0.0        75.8
##                                          44    1     1.1        76.8
##                                          45    1     1.1        77.9
##                                          49    2     2.1        80.0
##                                        49.5    1     1.1        81.1
##                                          50    0     0.0        81.1
##                                          51    1     1.1        82.1
##                                          53    0     0.0        82.1
##                                          56    0     0.0        82.1
##                                        <NA>   17    17.9       100.0
##
##    bl_hallucinations 95    0    0.0           0   46    48.4        48.4
##                                           1    8     8.4        56.8
##                                           2   16    16.8        73.7
##                                           3   10    10.5        84.2
##                                           4   10    10.5        94.7
##                                        <NA>    5     5.3       100.0
##
##    fu_hallucinations 95    0    0.0           0   47    49.5        49.5
##                                           1    5     5.3        54.7
##                                           2    7     7.4        62.1
##                                           3   11    11.6        73.7
##                                           4   12    12.6        86.3
##                                        <NA>   13    13.7       100.0
##
##       hallucinations 95    0    0.0          -4    2     2.1         2.1
##                                          -3    2     2.1         4.2
##                                          -2    2     2.1         6.3
##                                          -1    8     8.4        14.7
##                                           0   41    43.2        57.9
##                                           1   12    12.6        70.5
##                                           2    8     8.4        78.9
##                                           3    2     2.1        81.1
##                                           4    1     1.1        82.1
##                                        <NA>   17    17.9       100.0
##
```

```
##   hallucinations_bin 95    0    0.0 not improved   55    57.9        57.9
##                                         improved   23    24.2        82.1
##                                             <NA>   17    17.9       100.0
##
##         bl_delusions 95    0    0.0            0   27    28.4        28.4
##                                                1   10    10.5        38.9
##                                                2   23    24.2        63.2
##                                                3   12    12.6        75.8
##                                                4   19    20.0        95.8
##                                             <NA>    4     4.2       100.0
##
##         fu_delusions 95    0    0.0            0   30    31.6        31.6
##                                                1    8     8.4        40.0
##                                                2   11    11.6        51.6
##                                                3   16    16.8        68.4
##                                                4   17    17.9        86.3
##                                             <NA>   13    13.7       100.0
##
##            delusions 95    0    0.0           -4    3     3.2         3.2
##                                               -3    2     2.1         5.3
##                                               -2    8     8.4        13.7
##                                               -1    6     6.3        20.0
##                                                0   30    31.6        51.6
##                                                1   17    17.9        69.5
##                                                2   11    11.6        81.1
##                                                3    1     1.1        82.1
##                                                4    1     1.1        83.2
##                                             <NA>   16    16.8       100.0
##
##        delusions_bin 95    0    0.0 not improved   49    51.6        51.6
##                                         improved   30    31.6        83.2
##                                             <NA>   16    16.8       100.0
##
##        n_drug_shifts 95    0    0.0            0   86    90.5        90.5
##                                                1    6     6.3        96.8
##                                                2    2     2.1        98.9
##                                                3    1     1.1       100.0
##
##      bin_drug_shifts 95    0    0.0            0   86    90.5        90.5
##                                                1    9     9.5       100.0
##
##             retained 95    0    0.0          yes   95   100.0       100.0
##
##  n_shifts_all_binned 95    0    0.0            0   34    35.8        35.8
##                                                1   14    14.7        50.5
##                                                2   17    17.9        68.4
##                                                3    7     7.4        75.8
##                                               4+   23    24.2       100.0
##
## ---------------------------------------------------------------------------------------------------
----------------------------------------------------------------------------------------------------
----------------------------------------------------------------------------------------------------
----------------------------------------------------------------------------------------------------
----------------------------------------------------------------------------------------------------
----------------------------------------------------------------------------------------------------
---
## group: scm
##                  var  n miss p.miss        level freq percent cum.percent
##                group 94    0    0.0          ctl    0     0.0         0.0
```

© 2020 Jürgens G et al. *JAMA Network Open.*

```
##                                                ctg    0     0.0         0.0
##                                                scm   94   100.0       100.0
##
##                 event 94    0    0.0             0   38    40.4        40.4
##                                                   1   56    59.6       100.0
##
##                gender 94    0    0.0        female   43    45.7        45.7
##                                                male   51    54.3       100.0
##
##     extr_metabolizer 94    0    0.0            no   76    80.9        80.9
##                                                yes   18    19.1       100.0
##
##     poor_metabolizer 94    0    0.0            no   80    85.1        85.1
##                                                yes   14    14.9       100.0
##
##     fast_metabolizer 94    0    0.0            no   90    95.7        95.7
##                                                yes    4     4.3       100.0
##
##                  saei 94    0    0.0            no   78    83.0        83.0
##                                                yes   16    17.0       100.0
##
##             icd_major 94    0    0.0           F20   73    77.7        77.7
##                                                F21   13    13.8        91.5
##                                                F22    1     1.1        92.6
##                                                F23    0     0.0        92.6
##                                                F25    4     4.3        96.8
##                                                F28    1     1.1        97.9
##                                                F29    1     1.1        98.9
##                                               <NA>    1     1.1       100.0
##
##         cyp2c19_pheno 94    0    0.0            EM   68    72.3        72.3
##                                                 IM   24    25.5        97.9
##                                                 PM    2     2.1       100.0
##
##          cyp2d6_pheno 94    0    0.0            EM   46    48.9        48.9
##                                                 IM   32    34.0        83.0
##                                                 PM   12    12.8        95.7
##                                                 UM    4     4.3       100.0
##
##       cyp2d6_dep_drug 94    0    0.0         FALSE   12    12.8        12.8
##                                               TRUE   82    87.2       100.0
##
##      cyp2c19_dep_drug 94    0    0.0         FALSE   71    75.5        75.5
##                                               TRUE   23    24.5       100.0
##
##          cyp_dep_drug 94    0    0.0         FALSE   11    11.7        11.7
##                                               TRUE   83    88.3       100.0
##
##                fu_uku 94    0    0.0             0    4     4.3         4.3
##                                                  1    1     1.1         5.3
##                                                  2    1     1.1         6.4
##                                                2.5    0     0.0         6.4
##                                                  3    0     0.0         6.4
##                                                  4    0     0.0         6.4
##                                                  5    1     1.1         7.4
##                                                  6    1     1.1         8.5
##                                                  7    2     2.1        10.6
##                                                  8    1     1.1        11.7
##                                                8.5    1     1.1        12.8
```

© 2020 Jürgens G et al. *JAMA Network Open.*

```
##                                             9   2    2.1        14.9
##                                            10   2    2.1        17.0
##                                            11   4    4.3        21.3
##                                            12   3    3.2        24.5
##                                            13   1    1.1        25.5
##                                            14   2    2.1        27.7
##                                            15   6    6.4        34.0
##                                            16   1    1.1        35.1
##                                            17   4    4.3        39.4
##                                          17.5   0    0.0        39.4
##                                            18   4    4.3        43.6
##                                          18.5   0    0.0        43.6
##                                            19   1    1.1        44.7
##                                          19.5   0    0.0        44.7
##                                            20   2    2.1        46.8
##                                            21   2    2.1        48.9
##                                            22   4    4.3        53.2
##                                            23   3    3.2        56.4
##                                            24   0    0.0        56.4
##                                            25   1    1.1        57.4
##                                          25.5   0    0.0        57.4
##                                            26   5    5.3        62.8
##                                          26.5   1    1.1        63.8
##                                            27   1    1.1        64.9
##                                            28   1    1.1        66.0
##                                            29   2    2.1        68.1
##                                            30   2    2.1        70.2
##                                            31   2    2.1        72.3
##                                            32   0    0.0        72.3
##                                            33   0    0.0        72.3
##                                            34   1    1.1        73.4
##                                            35   0    0.0        73.4
##                                            36   1    1.1        74.5
##                                            37   1    1.1        75.5
##                                            38   1    1.1        76.6
##                                            39   1    1.1        77.7
##                                            40   1    1.1        78.7
##                                            41   0    0.0        78.7
##                                            42   1    1.1        79.8
##                                            44   0    0.0        79.8
##                                            45   2    2.1        81.9
##                                            49   0    0.0        81.9
##                                          49.5   0    0.0        81.9
##                                            50   1    1.1        83.0
##                                            51   0    0.0        83.0
##                                            53   0    0.0        83.0
##                                            56   0    0.0        83.0
##                                          <NA>  16   17.0       100.0
##
##    bl_hallucinations 94    0    0.0          0  40   42.6        42.6
##                                             1   5    5.3        47.9
##                                             2  19   20.2        68.1
##                                             3   7    7.4        75.5
##                                             4  14   14.9        90.4
##                                          <NA>   9    9.6       100.0
##
##    fu_hallucinations 94    0    0.0          0  42   44.7        44.7
##                                             1   7    7.4        52.1
##                                             2   7    7.4        59.6
```

© 2020 Jürgens G et al. *JAMA Network Open.*

```
##                                           3    7     7.4         67.0
##                                           4   17    18.1         85.1
##                                        <NA>   14    14.9        100.0
##
##       hallucinations 94    0    0.0          -4    1     1.1          1.1
##                                          -3    3     3.2          4.3
##                                          -2    5     5.3          9.6
##                                          -1    7     7.4         17.0
##                                           0   40    42.6         59.6
##                                           1    9     9.6         69.1
##                                           2    8     8.5         77.7
##                                           3    1     1.1         78.7
##                                           4    0     0.0         78.7
##                                        <NA>   20    21.3        100.0
##
##   hallucinations_bin 94    0    0.0 not improved   56    59.6         59.6
##                                    improved   18    19.1         78.7
##                                        <NA>   20    21.3        100.0
##
##         bl_delusions 94    0    0.0           0   21    22.3         22.3
##                                           1    4     4.3         26.6
##                                           2   23    24.5         51.1
##                                           3   18    19.1         70.2
##                                           4   22    23.4         93.6
##                                        <NA>    6     6.4        100.0
##
##         fu_delusions 94    0    0.0           0   27    28.7         28.7
##                                           1    7     7.4         36.2
##                                           2   17    18.1         54.3
##                                           3   14    14.9         69.1
##                                           4   14    14.9         84.0
##                                        <NA>   15    16.0        100.0
##
##            delusions 94    0    0.0          -4    0     0.0          0.0
##                                          -3    1     1.1          1.1
##                                          -2    6     6.4          7.4
##                                          -1    8     8.5         16.0
##                                           0   32    34.0         50.0
##                                           1   10    10.6         60.6
##                                           2   12    12.8         73.4
##                                           3    7     7.4         80.9
##                                           4    0     0.0         80.9
##                                        <NA>   18    19.1        100.0
##
##        delusions_bin 94    0    0.0 not improved   47    50.0         50.0
##                                    improved   29    30.9         80.9
##                                        <NA>   18    19.1        100.0
##
##        n_drug_shifts 94    0    0.0           0   87    92.6         92.6
##                                           1    5     5.3         97.9
##                                           2    2     2.1        100.0
##                                           3    0     0.0        100.0
##
##      bin_drug_shifts 94    0    0.0           0   87    92.6         92.6
##                                           1    7     7.4        100.0
##
##             retained 94    0    0.0         yes   94   100.0        100.0
##
##  n_shifts_all_binned 94    0    0.0           0   39    41.5         41.5
```

© 2020 Jürgens G et al. *JAMA Network Open.*

```
##                                              1   20    21.3         62.8
##                                              2   12    12.8         75.5
##                                              3    5     5.3         80.9
##                                             4+   18    19.1        100.0
##
##
## Standardize mean differences
##                         average      1 vs 2      1 vs 3      2 vs 3
## group                       NaN         NaN         NaN         NaN
## event               0.049091586 0.016169662 0.057459907 0.073645189
## gender              0.006446479 0.005651883 0.004017808 0.009669745
## extr_metabolizer    0.031677314 0.006405678 0.041110004 0.047516259
## poor_metabolizer    0.052819022 0.054372051 0.079224944 0.024860070
## fast_metabolizer    0.077284382 0.115599100 0.068902673 0.047351372
## saei                0.039539679 0.026044077 0.059307547 0.033267414
## icd_major           0.363784769 0.298532720 0.421590046 0.371231543
## cyp2c19_pheno       0.107314122 0.160633611 0.143420212 0.017888544
## cyp2d6_pheno        0.092954344 0.118946015 0.091463954 0.068453062
## cyp2d6_dep_drug     0.160166037 0.070436975 0.240224210 0.169836925
## cyp2c19_dep_drug    0.093882766 0.140829804 0.006602411 0.134216083
## cyp_dep_drug        0.115483629 0.054372051 0.173164956 0.118913880
## fu_uku              1.188755949 1.154357049 1.236402321 1.175508477
## bl_hallucinations   0.296563639 0.205738101 0.401313344 0.282639473
## fu_hallucinations   0.383535250 0.428734200 0.495200197 0.226671352
## hallucinations      0.359317386 0.420671489 0.366918328 0.290362342
## hallucinations_bin  0.160525090 0.235709797 0.110884724 0.134980749
## bl_delusions        0.339947188 0.227727163 0.457710786 0.334403616
## fu_delusions        0.319680135 0.430995986 0.317571549 0.210472869
## delusions           0.497300714 0.521954136 0.462146387 0.507801620
## delusions_bin       0.257039645 0.371380228 0.339564148 0.060174559
## n_drug_shifts       0.197991689 0.226181589 0.214997209 0.152796269
## bin_drug_shifts     0.139639213 0.136994721 0.209041150 0.072881769
## retained            0.000000000 0.000000000 0.000000000 0.000000000
## n_shifts_all_binned 0.181514890 0.082262853 0.199750676 0.262531142
```

Select summary stats across arms

```
CreateTableOne(vars = c("age", "illness_duration", "gender", "icd_major"),
               factorVars = c("gender", "icd_major"),
               includeNA = TRUE,
               test = FALSE,
               data = table1_df) %>%
  summary()

##
##      ### Summary of continuous variables ###
##
## strata: Overall
##                    n miss p.miss mean sd median p25 p75  min max skew kurt
## age              311    0      0   41 13     41  30  50 18.7  73  0.2 -0.9
## illness_duration 311   33     11    9  9      6   3  13  0.2  50  1.5  2.1
##
## =======================================================================================
##
##      ### Summary of categorical variables ###
##
## strata: Overall
##        var   n miss p.miss  level freq percent cum.percent
##     gender 311    0    0.0 female  139    44.7        44.7
```

© 2020 Jürgens G et al. *JAMA Network Open.*

```
##                             male  172    55.3       100.0
##
##  icd_major 311    0    0.0     F20  216    69.5        69.5
##                                F21   63    20.3        89.7
##                                F22    7     2.3        92.0
##                                F23    3     1.0        92.9
##                                F25   14     4.5        97.4
##                                F28    1     0.3        97.7
##                                F29    1     0.3        98.1
##                               <NA>    6     1.9       100.0
##
```

Summary stats for those not retained

```
CreateTableOne(vars = c("age", "gender", "icd_major", "illness_duration", "cyp2d6_pheno", "cyp2c19_p
heno"),
               strata = c("group", "retained"),
               factorVars = c("icd_major", "gender", "cyp2d6_pheno", "cyp2c19_pheno"),
               includeNA = TRUE,
               test = FALSE,
               data = table1_df) %>%
  summary()

##
##      ### Summary of continuous variables ###
##
## group: ctl
## retained: no
##                  n miss p.miss mean sd median p25 p75  min max skew kurt
## age              5    0      0   35 18     26  23  40 19.6  63  1.4    1
## illness_duration 5    1     20    4  3      4   3   6  0.6   8  0.2    1
## ----------------------------------------------------------------------------------------------------
----------------------------------------------------------------------------------------------------
----------------------------------------------------------------------------------------------------
----------------------------------------------------------------------------------------------------
----------------------------------------------------------------------------------------------------
----------------------------------------------------------------------------------------------------
---
## group: ctg
## retained: no
##                  n miss p.miss mean sd median p25 p75  min max skew kurt
## age              8    0      0   32 13     28  25  33 22.8  63  2.3  5.6
## illness_duration 8    1     12    3  3      3   1   5  0.5   8  0.5 -0.8
## ----------------------------------------------------------------------------------------------------
----------------------------------------------------------------------------------------------------
----------------------------------------------------------------------------------------------------
----------------------------------------------------------------------------------------------------
----------------------------------------------------------------------------------------------------
----------------------------------------------------------------------------------------------------
---
## group: scm
## retained: no
##                  n miss p.miss mean sd median p25 p75 min max skew kurt
## age              8    0      0   31 11     28  26  34  19  57    2    4
## illness_duration 8    1     12    5  4      3   2   6   1  13    1    1
## ----------------------------------------------------------------------------------------------------
----------------------------------------------------------------------------------------------------
----------------------------------------------------------------------------------------------------
----------------------------------------------------------------------------------------------------
```

© 2020 Jürgens G et al. *JAMA Network Open.*

```
-----------------------------------------------------------------------------------------
-----------------------------------------------------------------------------------------
---
## group: ctl
## retained: yes
##                    n miss p.miss mean sd median p25 p75  min max skew kurt
## age              101    0      0   42 13     42  31  53 20.1  73  0.2 -0.8
## illness_duration 101    9      9   10  9      7   3  13  0.2  38  1.3  1.2
## --------------------------------------------------------------------------------------
-----------------------------------------------------------------------------------------
-----------------------------------------------------------------------------------------
-----------------------------------------------------------------------------------------
-----------------------------------------------------------------------------------------
-----------------------------------------------------------------------------------------
---
## group: ctg
## retained: yes
##                   n miss p.miss mean sd median p25 p75  min max skew kurt
## age              95    0      0   42 13     42  32  51 19.0  68 0.05   -1
## illness_duration 95   10     11   10  9      7   3  15  0.2  50 1.64    4
## --------------------------------------------------------------------------------------
-----------------------------------------------------------------------------------------
-----------------------------------------------------------------------------------------
-----------------------------------------------------------------------------------------
-----------------------------------------------------------------------------------------
-----------------------------------------------------------------------------------------
---
## group: scm
## retained: yes
##                   n miss p.miss mean sd median p25 p75  min max skew kurt
## age              94    0      0   41 12     40  30  48 18.7  71  0.3 -0.7
## illness_duration 94   11     12   10  9      6   3  16  0.2  40  1.3  0.8
##
## Standardize mean differences
##                    average    1 vs 2    1 vs 3    1 vs 4    1 vs 5    1 vs 6     2 vs 3    2 vs 4
2 vs 5    2 vs 6    3 vs 4    3 vs 5    3 vs 6     4 vs 5      4 vs 6     5 vs 6
## age              0.4460617 0.1473411 0.2107401 0.4855889 0.4537129 0.3949138 0.06924558 0.7591365
0.7259139 0.6662275 0.8796631 0.8469351 0.7893797 0.04462711 0.131138840 0.08636213
## illness_duration 0.5467371 0.3646010 0.1272188 0.7983889 0.7894821 0.7610518 0.42687431 0.9777846
0.9632474 0.9328489 0.6870981 0.6824556 0.6564413 0.01007771 0.006857507 0.01662895
##
## ====================================================================================
##
##      ### Summary of categorical variables ###
##
## group: ctl
## retained: no
##            var n miss p.miss  level freq percent cum.percent
##         gender 5    0    0.0 female    1    20.0        20.0
##                                male    4    80.0       100.0
##
##      icd_major 5    0    0.0    F20    3    60.0        60.0
##                                 F21    1    20.0        80.0
##                                 F22    1    20.0       100.0
##                                 F23    0     0.0       100.0
##                                 F25    0     0.0       100.0
##                                 F28    0     0.0       100.0
##                                 F29    0     0.0       100.0
##                                <NA>    0     0.0       100.0
```

© 2020 Jürgens G et al. *JAMA Network Open.*

```
##
##   cyp2d6_pheno 5    0    0.0     EM    3    60.0        60.0
##                                  IM    2    40.0       100.0
##                                  PM    0     0.0       100.0
##                                  UM    0     0.0       100.0
##
##  cyp2c19_pheno 5    0    0.0     EM    3    60.0        60.0
##                                  IM    2    40.0       100.0
##                                  PM    0     0.0       100.0
##
## -------------------------------------------------------------------------------------------------
----------------------------------------------------------------------------------------------------
----------------------------------------------------------------------------------------------------
----------------------------------------------------------------------------------------------------
----------------------------------------------------------------------------------------------------
----------------------------------------------------------------------------------------------------
---
## group: ctg
## retained: no
##            var n miss p.miss  level freq percent cum.percent
##         gender 8    0    0.0 female    3    37.5        37.5
##                                male    5    62.5       100.0
##
##      icd_major 8    0    0.0    F20    3    37.5        37.5
##                                 F21    5    62.5       100.0
##                                 F22    0     0.0       100.0
##                                 F23    0     0.0       100.0
##                                 F25    0     0.0       100.0
##                                 F28    0     0.0       100.0
##                                 F29    0     0.0       100.0
##                                <NA>    0     0.0       100.0
##
##   cyp2d6_pheno 8    0    0.0     EM    4    50.0        50.0
##                                  IM    4    50.0       100.0
##                                  PM    0     0.0       100.0
##                                  UM    0     0.0       100.0
##
##  cyp2c19_pheno 8    0    0.0     EM    6    75.0        75.0
##                                  IM    1    12.5        87.5
##                                  PM    1    12.5       100.0
##
## -------------------------------------------------------------------------------------------------
----------------------------------------------------------------------------------------------------
----------------------------------------------------------------------------------------------------
----------------------------------------------------------------------------------------------------
----------------------------------------------------------------------------------------------------
----------------------------------------------------------------------------------------------------
---
## group: scm
## retained: no
##            var n miss p.miss  level freq percent cum.percent
##         gender 8    0    0.0 female    3    37.5        37.5
##                                male    5    62.5       100.0
##
##      icd_major 8    0    0.0    F20    3    37.5        37.5
##                                 F21    5    62.5       100.0
##                                 F22    0     0.0       100.0
##                                 F23    0     0.0       100.0
##                                 F25    0     0.0       100.0
```

© 2020 Jürgens G et al. *JAMA Network Open.*

```
##                                 F28    0     0.0       100.0
##                                 F29    0     0.0       100.0
##                                <NA>    0     0.0       100.0
##
##   cyp2d6_pheno 8    0    0.0     EM    4    50.0        50.0
##                                  IM    3    37.5        87.5
##                                  PM    1    12.5       100.0
##                                  UM    0     0.0       100.0
##
##  cyp2c19_pheno 8    0    0.0     EM    6    75.0        75.0
##                                  IM    2    25.0       100.0
##                                  PM    0     0.0       100.0
##
## ----------------------------------------------------------------------------------------------------
----------------------------------------------------------------------------------------------------
----------------------------------------------------------------------------------------------------
----------------------------------------------------------------------------------------------------
----------------------------------------------------------------------------------------------------
----------------------------------------------------------------------------------------------------
---
## group: ctl
## retained: yes
##           var   n miss p.miss  level freq percent cum.percent
##        gender 101    0    0.0 female   46    45.5        45.5
##                                 male   55    54.5       100.0
##
##     icd_major 101    0    0.0    F20   66    65.3        65.3
##                                  F21   20    19.8        85.1
##                                  F22    2     2.0        87.1
##                                  F23    2     2.0        89.1
##                                  F25    8     7.9        97.0
##                                  F28    0     0.0        97.0
##                                  F29    0     0.0        97.0
##                                 <NA>    3     3.0       100.0
##
##   cyp2d6_pheno 101    0    0.0     EM   50    49.5        49.5
##                                    IM   33    32.7        82.2
##                                    PM   15    14.9        97.0
##                                    UM    3     3.0       100.0
##
##  cyp2c19_pheno 101    0    0.0     EM   78    77.2        77.2
##                                    IM   20    19.8        97.0
##                                    PM    3     3.0       100.0
##
## ----------------------------------------------------------------------------------------------------
----------------------------------------------------------------------------------------------------
----------------------------------------------------------------------------------------------------
----------------------------------------------------------------------------------------------------
----------------------------------------------------------------------------------------------------
----------------------------------------------------------------------------------------------------
---
## group: ctg
## retained: yes
##           var  n miss p.miss  level freq percent cum.percent
##        gender 95    0    0.0 female   43    45.3        45.3
##                                male   52    54.7       100.0
##
##     icd_major 95    0    0.0    F20   68    71.6        71.6
##                                 F21   19    20.0        91.6
```

© 2020 Jürgens G et al. *JAMA Network Open.*

```
##                                  F22    3     3.2        94.7
##                                  F23    1     1.1        95.8
##                                  F25    2     2.1        97.9
##                                  F28    0     0.0        97.9
##                                  F29    0     0.0        97.9
##                                 <NA>    2     2.1       100.0
##
##   cyp2d6_pheno 95    0    0.0     EM   47    49.5        49.5
##                                   IM   30    31.6        81.1
##                                   PM   13    13.7        94.7
##                                   UM    5     5.3       100.0
##
##  cyp2c19_pheno 95    0    0.0     EM   68    71.6        71.6
##                                   IM   25    26.3        97.9
##                                   PM    2     2.1       100.0
##
## -----------------------------------------------------------------------------------------------------
------------------------------------------------------------------------------------------------------
------------------------------------------------------------------------------------------------------
------------------------------------------------------------------------------------------------------
------------------------------------------------------------------------------------------------------
------------------------------------------------------------------------------------------------------
---
## group: scm
## retained: yes
##            var  n miss p.miss  level freq percent cum.percent
##         gender 94    0    0.0 female   43    45.7        45.7
##                                 male   51    54.3       100.0
##
##      icd_major 94    0    0.0    F20   73    77.7        77.7
##                                  F21   13    13.8        91.5
##                                  F22    1     1.1        92.6
##                                  F23    0     0.0        92.6
##                                  F25    4     4.3        96.8
##                                  F28    1     1.1        97.9
##                                  F29    1     1.1        98.9
##                                 <NA>    1     1.1       100.0
##
##   cyp2d6_pheno 94    0    0.0     EM   46    48.9        48.9
##                                   IM   32    34.0        83.0
##                                   PM   12    12.8        95.7
##                                   UM    4     4.3       100.0
##
##  cyp2c19_pheno 94    0    0.0     EM   68    72.3        72.3
##                                   IM   24    25.5        97.9
##                                   PM    2     2.1       100.0
##
##
## Standardize mean differences
##                 average    1 vs 2    1 vs 3    1 vs 4    1 vs 5    1 vs 6    2 vs 3    2 vs 4
2 vs 5    2 vs 6    3 vs 4    3 vs 5    3 vs 6      4 vs 5      4 vs 6      5 vs 6
## gender        0.2321314 0.3940927 0.3940927 0.5655556 0.5595029 0.5698647 0.0000000 0.1638015 0.1
581142 0.1678461 0.1638015 0.1581142 0.1678461 0.005651883 0.004017808 0.009669745
## icd_major     0.8099633 1.1098231 1.1098231 0.8048227 0.6391121 0.8046172 0.0000000 1.0705652 1.0
150110 1.2093724 1.0705652 1.0150110 1.2093724 0.298532720 0.421590046 0.371231543
## cyp2d6_pheno  0.4370675 0.2020305 0.5378738 0.6586057 0.6841282 0.6408851 0.5547002 0.6933095 0.7
228957 0.6678255 0.2671895 0.3464645 0.3012407 0.118946015 0.091463954 0.068453062
## cyp2c19_pheno 0.3594409 0.8164966 0.3244428 0.5042810 0.3501499 0.3657624 0.6030227 0.3962326 0.5
140889 0.5023375 0.2710053 0.2118367 0.2100151 0.160633611 0.143420212 0.017888544
```

© 2020 Jürgens G et al. *JAMA Network Open.*

O1: Antipsychotic persistence

We create a function to produce the KM plot, so we can easily create KM plots of various flavours.

```
km_plot <- function(which_main, which_sub = "none", which_ref = which_main) {
  data_main <- switch(which_main,
                      extr = filter(df, extr_metabolizer == "yes"),
                      non_extr = filter(df, extr_metabolizer == "no"),
                      df)
  data_sub <- switch(which_sub,
                     extr = filter(df, extr_metabolizer == "yes"),
                     non_extr = filter(df, extr_metabolizer == "no"),
                     df)
  data_ref <- switch(which_ref,
                     extr = filter(df, extr_metabolizer == "yes"),
                     non_extr = filter(df, extr_metabolizer == "no"),
                     df)
  title_sub <- switch(which_sub,
                      extr = "Extreme metabolizers",
                      non_extr = "Non-extreme metabolizers",
                      "All patients")
  label_x <- switch(which_main, extr = c(178, 313, 115), c(22, 245, 115))
  label_y <- switch(which_main, extr = c(0.38, 0.2, 0.94), c(0.73, 0.39, 0.9))
  label_df <- data.frame(x = label_x, y = label_y,
                         col = paste0("group=", c("ctl", "ctg", "scm"))) %>%
      mutate(label = pretty[c("ctl", "ctg", "scm")])

  # Create inset plot
  survfit_sub <- survfit(Surv(persistence, event) ~ group, data = data_sub)
  survfit_sub_ref <- survfit(Surv(persistence, event) ~ 1, data = data_ref) %>%
    surv_summary() %>%
    { ggsurvplot_df(., color = "black", size = 0.3, linetype = "solid")$layers[[1]] }

  surv_sub <- ggsurvplot(survfit_sub, data = data_sub, size = 0.3, legend = "none",
                         break.time.by = 80, break.y.by = 0.25, risk.table = FALSE,
                         fontsize = geom_text_size, title = title_sub, palette = "Set1",
                         ggtheme = default_theme, censor = FALSE, newpage = FALSE)
  surv_sub$plot <- surv_sub$plot + survfit_sub_ref + guides(colour = FALSE)
  surv_sub$plot$layers <- rev(surv_sub$plot$layers) # put ref line in the back
  surv_sub$plot$theme <- surv_sub$plot$theme + plot_theme(0.6, FALSE)

  # Create main plot
  survfit_main <- survfit(Surv(persistence, event) ~ group, data = data_main)
  surv_main <- ggsurvplot(survfit_main, data = df, size = 0.5, xlab = "Persistence (days)",
                          legend = "none", palette = "Set1",break.time.by = 40,
                          risk.table = TRUE, risk.table.y.text = FALSE,
                          risk.table.height = 0.21, censor = FALSE,
                          risk.table.fontsize = geom_text_size, ggtheme = default_theme)

  # Customise main plots layout
  surv_main$plot$theme <- surv_main$plot$theme + plot_theme()
  surv_main$plot <- surv_main$plot +
    labs(y = "Proportion") +
    scale_y_continuous(limits = c(0, 1.05), breaks = seq(0, 1, 0.25)) +
    geom_text(aes(x, y, label = label, colour = col), label_df,
              size = text_size / ggplot2::.pt) +
    guides(colour = FALSE)
```

© 2020 Jürgens G et al. *JAMA Network Open.*

```
  # Add inset, if relevant
  if (which_sub != "none")
    surv_main$plot <- surv_main$plot +
      annotation_custom(grobTree(rectGrob(gp = gpar(size = 0.3)),
                                 ggplotGrob(surv_sub$plot)),
                        xmin = 250, xmax = 400, ymin = 0.55, ymax = 1.05)

  # Customise risk table layout
  surv_main$table$theme <- surv_main$table$theme +
    theme(axis.line = element_blank(),
          panel.background = element_blank(),
          axis.text.y = element_text(size = text_size),
          axis.text.x = element_blank(), axis.ticks = element_blank(),
          plot.title = element_text(size = text_size * 1.1),
          axis.title.y = element_text(size = text_size),
          axis.title.x = element_blank(),
          panel.grid = element_blank(),
          panel.grid.major = element_blank())
  surv_main$table <- surv_main$table +
    labs(title = "Number of patients at risk", y = "")

  surv_main # return final plot
}
```

eFigure 2: KM curves of all and extreme metabolizers only

Figure 2 to become two-panel.

```
km_plot("all") # export as PDF file, 6.65 x 4.71 inch.
```

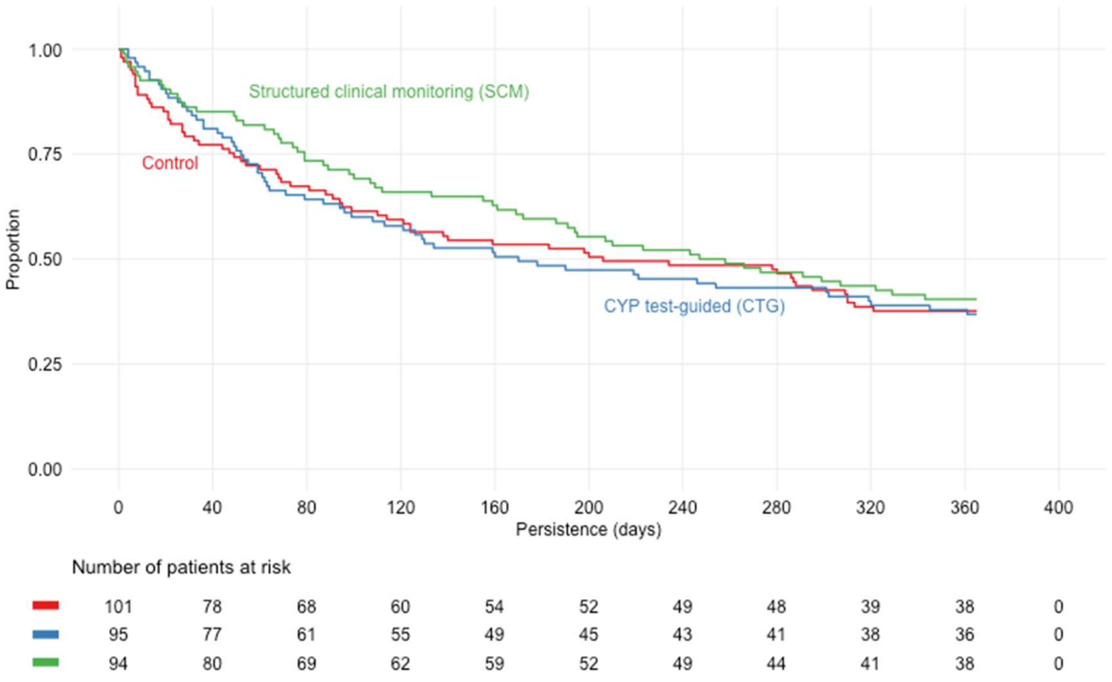


```
km_plot("extr")
```

© 2020 Jürgens G et al. *JAMA Network Open.*

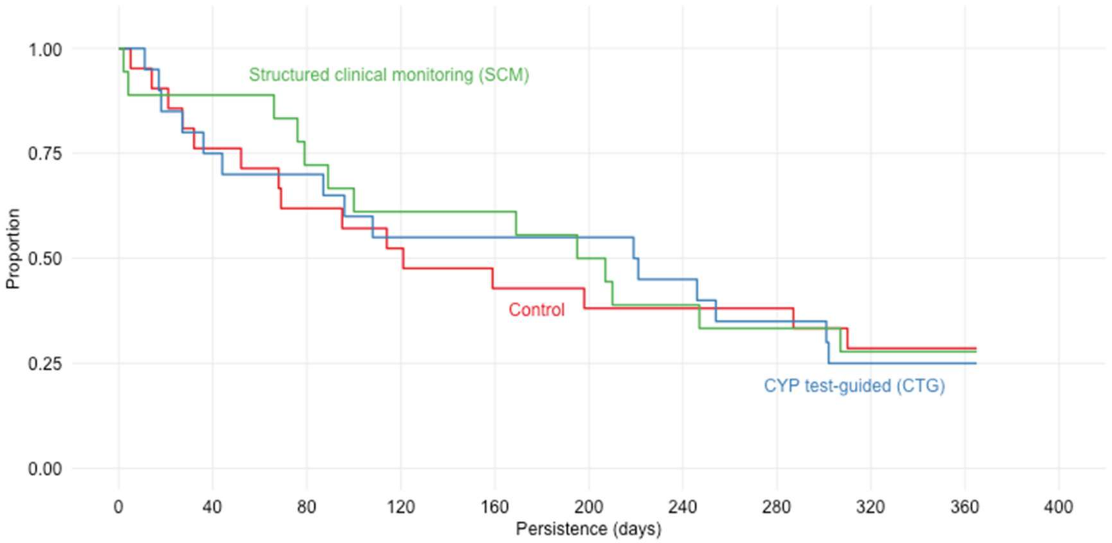

Number of patients at risk

	0	40	80	120	160	200	240	280	320	360	400
Control (red)	21	16	13	11	9	8	8	8	6	6	0
CTG (blue)	20	15	14	11	11	11	9	7	5	5	0
SCM (green)	18	16	13	11	11	9	7	6	5	5	0

In-text: Cox regression model, all patients

Will be reported in-text.

```
cox_model_all_null <- coxph(Surv(persistence, event) ~ 1, data = df, ties = "efron",
                            model = TRUE)
cox_model_all_base <- update(cox_model_all_null, formula = ~ . + group)

for (m in c("null", "base")) {
  mod <- get(paste0("cox_model_all_", m))
  cat("\nSummary of", m, "model:\n")
  print(summary(mod))
}

##
## Summary of null model:
## Call:  coxph(formula = Surv(persistence, event) ~ 1, data = df, ties = "efron",
##     model = TRUE)
##
## Null model
##   log likelihood= -942.9873
##   n=290 (21 observations deleted due to missingness)
##
## Summary of base model:
## Call:
## coxph(formula = Surv(persistence, event) ~ group, data = df,
##     ties = "efron", model = TRUE)
##
##   n= 290, number of events= 179
##    (21 observations deleted due to missingness)
##
##              coef exp(coef) se(coef)      z Pr(>|z|)
## groupctg  0.0165    1.0166   0.1804  0.091    0.927
```

© 2020 Jürgens G et al. *JAMA Network Open.*

```
## groupscm -0.1332    0.8753   0.1837 -0.725    0.469
##
##          exp(coef) exp(-coef) lower .95 upper .95
## groupctg    1.0166     0.9836    0.7138     1.448
## groupscm    0.8753     1.1424    0.6107     1.255
##
## Concordance= 0.521  (se = 0.02 )
## Likelihood ratio test= 0.79  on 2 df,   p=0.7
## Wald test            = 0.78  on 2 df,   p=0.7
## Score (logrank) test = 0.78  on 2 df,   p=0.7
```

Proportional hazards assumption

The KM curves cross, so the proportional hazards assumption doesn't hold per se. However, for completeness we conduct numeric and visual evaluation of the assumption. We plot the scaled Schoenfeld residuals vs. time to assert that the residuals of each variable are not time-dependent (i.e., non-zero slopes). The blue lines in these plots are LOESS-smoothed curves.

```
(prop_hazards_fit <- cox.zph(cox_model_all_base, transform = "identity"))

##        chisq df    p
## group   1.59  2 0.45
## GLOBAL  1.59  2 0.45

y <- prop_hazards_fit$y # y <- apply(prop_hazards_fit$y, 2, cumsum) would yield cum. sums
data.frame(t = prop_hazards_fit$x, y, row.names = NULL) %>%
  gather(group, value, -t) %>%
  mutate(group = pretty[group]) %>%
  ggplot(aes(x = t, y = value)) +
    geom_jitter(width = 0.1, height = 0.3) +
    geom_smooth(method = "loess", size = 0.3, alpha = 0.2, colour = "blue") +
    facet_wrap(~ group) +
    labs(x = "Time in days", y = "Scaled Schoenfeld residuals (with slight jitter)")

## `geom_smooth()` using formula 'y ~ x'
```

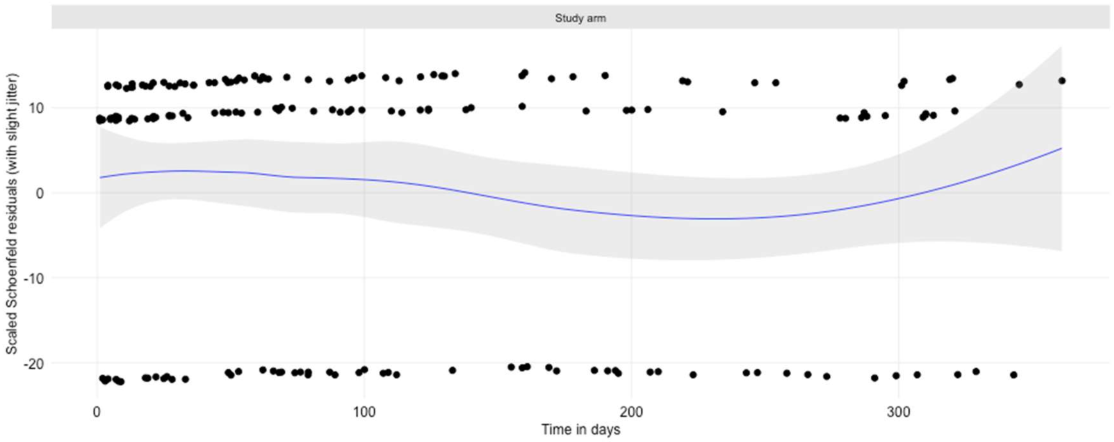

In-text: Cox regression model, extreme metabolizers

```
cox_model_extr_base <- update(cox_model_all_base, subset = extr_metabolizer == "yes")
summary(cox_model_extr_base)

## Call:
## coxph(formula = Surv(persistence, event) ~ group, data = df,
##     subset = extr_metabolizer == "yes", ties = "efron", model = TRUE)
```

© 2020 Jürgens G et al. *JAMA Network Open.*

```
##
##   n= 59, number of events= 43
##    (2 observations deleted due to missingness)
##
##                coef exp(coef)  se(coef)      z Pr(>|z|)
## groupctg -0.008961  0.991079  0.365792 -0.024    0.980
## groupscm -0.069653  0.932717  0.379415 -0.184    0.854
##
##          exp(coef) exp(-coef) lower .95 upper .95
## groupctg    0.9911      1.009    0.4839     2.030
## groupscm    0.9327      1.072    0.4434     1.962
##
## Concordance= 0.516  (se = 0.045 )
## Likelihood ratio test= 0.04  on 2 df,   p=1
## Wald test            = 0.04  on 2 df,   p=1
## Score (logrank) test = 0.04  on 2 df,   p=1
```

Proportional hazards assumption

```
(prop_hazards_fit_extr <- cox.zph(cox_model_extr_base, transform = "identity"))

##        chisq df    p
## group  0.838  2 0.66
## GLOBAL 0.838  2 0.66

data.frame(t = prop_hazards_fit_extr$x, prop_hazards_fit_extr$y, row.names = NULL) %>%
  gather(group, value, -t) %>%
  mutate(group = pretty[group]) %>%
  ggplot(aes(x = t, y = value)) +
    geom_jitter(width = 0.1, height = 0.3) +
    geom_smooth(method = "loess", size = 0.3, alpha = 0.2, colour = "blue") +
    facet_wrap(~ group) +
    labs(x = "Time in days", y = "Scaled Schoenfeld residuals (with slight jitter)")

## `geom_smooth()` using formula 'y ~ x'
```

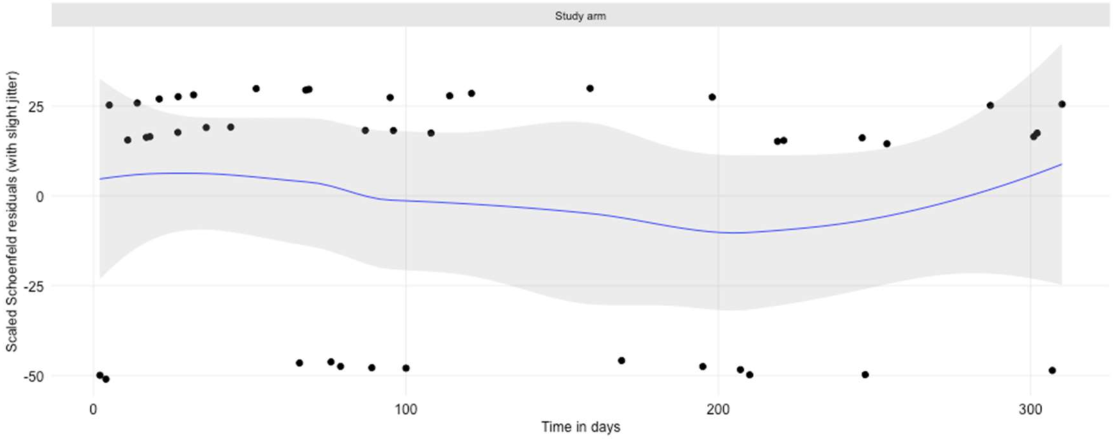

O2a: Changes in drugs and/or dose

Summary statistics:

```
select(df, group, n_shifts_all, n_drug_shifts) %>%
  gather(key, n, -group) %>%
  group_by(key, group) %>%
```

© 2020 Jürgens G et al. *JAMA Network Open.*

```r
  summarise(p50 = quantile(n, 0.5), # = median
    p25 = quantile(n, 0.25),
    p75 = quantile(n, 0.75),
    min = min(n),
    max = max(n),
    mean = mean(n),
    sd = sd(n)) %>%
  gather(statistic, value, -key, -group) %>%
  { ftable(xtabs(signif(value, 1) ~ statistic + key + group, data = .),
           row.vars = "statistic") }

## `summarise()` regrouping output by 'key' (override with `.groups` argument)

##           key   n_drug_shifts             n_shifts_all
##           group           ctl   ctg   scm          ctl   ctg   scm
## statistic
## max                      2.00  3.00  2.00        20.00 30.00 10.00
## mean                     0.20  0.10  0.09         2.00  3.00  2.00
## min                      0.00  0.00  0.00         0.00  0.00  0.00
## p25                      0.00  0.00  0.00         0.00  0.00  0.00
## p50                      0.00  0.00  0.00         1.00  1.00  1.00
## p75                      0.00  0.00  0.00         3.00  3.00  2.00
## sd                       0.40  0.50  0.30         4.00  5.00  2.00
```

Figure 3: Any changes (dose and/or drug)

The distributions across study arms are roughly identical. The counts of drugs and doses combined follow a negative binomial distribution reasonably well.

```r
p1 <- ggplot1() +
  plot_theme() +
  stat_count(aes(rnbinom(10000, 0.5071, mu = 2.25), ..prop..), geom = "line",
           size = 0.5, linetype = 2) +
  stat_count(aes(n_shifts_all, ..prop.., colour = group), df, geom = "line",
           size = 0.5, position = "identity") +
  guides(colour = FALSE) +
  labs(x = "Number of shifts in drugs and/or dose", y = "") +
  coord_cartesian(xlim = c(0, 33))

p2 <- ggplot1() +
  plot_theme() +
  stat_count(aes(n_drug_shifts, ..prop.., colour = group), df, geom = "line",
           size = 0.5, position = "identity") +
  guides(colour = FALSE) +
  labs(x = "Number of drug shifts", y = "")

grid.arrange(p1, p2, nrow = 1) # export as PDF file, 6.65 x 2.68 inch.
```

© 2020 Jürgens G et al. *JAMA Network Open.*

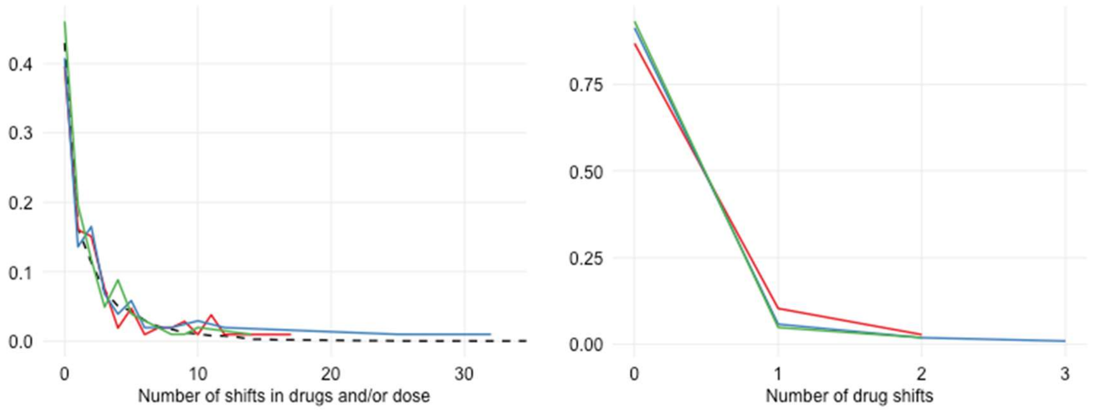

Negative binomial model for drug and/dose changes

We model directly the count vs. group, i.e., we do not log-transform the outcome variable. Thus, the coefficients of the group levels represent the change in counts compared to the control group. In this chunk, we define a function that runs the entire analysis, based on the input.

```
neg_binom_shifts <- function(dft, label = "all patients") {
  mod_null <- glm.nb(n_shifts_all ~ 1, data = dft, link = "identity")
  mod_base <- update(mod_null, formula = . ~ . + group)

  cat("> Base model:\n")
  print(tidy_res(mod_base))
  x <- with(summary(mod_base), round(c(theta, SE.theta), 2))
  cat(sprintf("Dispersion parameter (std. error): %s (%s)\n", x[1], x[2]))

  cat("\n> Comparing null and base models, log-likelihood test:\n")
  print(anova(mod_null, mod_base))

  cat("\n> Comparing null and base models, AIC and BIC:\n")
  matrix(c(AIC(mod_null, mod_base)$AIC, BIC(mod_null, mod_base)$BIC), nrow = 2,
         dimnames = list(c("mod_null", "mod_base"), c("AIC", "BIC"))) %>%
    addmargins(margin = 1, FUN = diff) %>%
    print()

  p1 <- ggplot() +
    geom_histogram(aes(x = rstudent(mod_base)), binwidth = 0.1, fill = "white",
                   colour = "black", size = 0.5) +
    geom_vline(aes(xintercept = c(-1.96, 1.96)), linetype = 2, colour = "red") +
    xlab("Studentised residuals")

  p2 <- gather(data.frame(dfbeta(mod_base)), predictor, value) %>%
    mutate(predictor = pretty[predictor]) %>%
    ggplot2(aes(x = value, y = ..density..)) +
      stat_density(aes(colour = predictor), geom = "line", size = 0.5,
                   show.legend = FALSE) +
      facet_wrap(~ predictor, scales = "free") +
      xlab("DF beta values")

  grid.arrange(p1, p2, ncol = 1, top = paste("Diagnostic plots for", label,
                                             "and all shifts"))
}
```

© 2020 Jürgens G et al. *JAMA Network Open.*

All patients

Fit the negative binomial model for all patients.

```
neg_binom_shifts(df, "all patients")

## > Base model:
## # A tibble: 3 x 4
##   term                        estimate conf.low conf.high
##   <chr>                       <chr>    <chr>    <chr>
## 1 (Intercept)                 2.50     1.89     3.37
## 2 Group: CTG (vs. control) 0.10     -0.98    1.21
## 3 Group: SCM (vs. control) -0.87    -1.85    -0.01
## Dispersion parameter (std. error): 0.52 (0.06)
##
## > Comparing null and base models, log-likelihood test:
## Likelihood ratio tests of Negative Binomial Models
##
## Response: n_shifts_all
##   Model     theta Resid. df    2 x log-lik.   Test    df LR stat.    Pr(Chi)
## 1     1 0.5071184       310       -1214.920
## 2 group 0.5228318       308       -1209.492 1 vs 2     2  5.42862 0.06625064
##
## > Comparing null and base models, AIC and BIC:
##                 AIC         BIC
## mod_null 1218.92032 1226.399909
## mod_base 1217.49170 1232.450874
## diff       -1.42862    6.050965
```

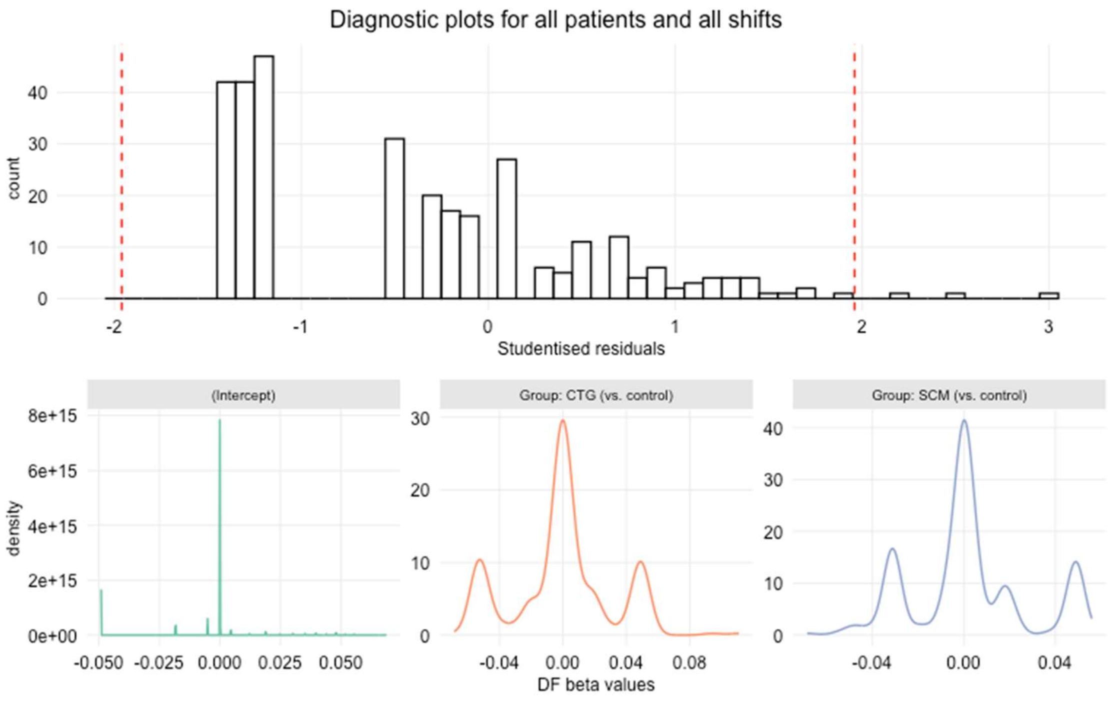

Extreme metabolizers

Then, fit the same model using extreme metabolizers only.

© 2020 Jürgens G et al. *JAMA Network Open.*

```
neg_binom_shifts(filter(df, extr_metabolizer == "yes"), "extreme metabolizers")

## > Base model:
## # A tibble: 3 x 4
##   term                       estimate conf.low conf.high
##   <chr>                      <chr>    <chr>    <chr>
## 1 (Intercept)                3.62     2.20     6.33
## 2 Group: CTG (vs. control) -1.19    -4.09    1.19
## 3 Group: SCM (vs. control) -2.25    -5.04    -0.36
## Dispersion parameter (std. error): 0.81 (0.23)
##
## > Comparing null and base models, log-likelihood test:
## Likelihood ratio tests of Negative Binomial Models
##
## Response: n_shifts_all
##   Model     theta Resid. df    2 x log-lik.   Test    df LR stat.    Pr(Chi)
## 1     1 0.7042931        60       -254.0829
## 2 group 0.8100803        58       -249.0045 1 vs 2     2 5.078364 0.07893095
##
## > Comparing null and base models, AIC and BIC:
##                   AIC         BIC
## mod_null 258.082900 262.304648
## mod_base 257.004537 265.448032
## diff      -1.078364   3.143384
```

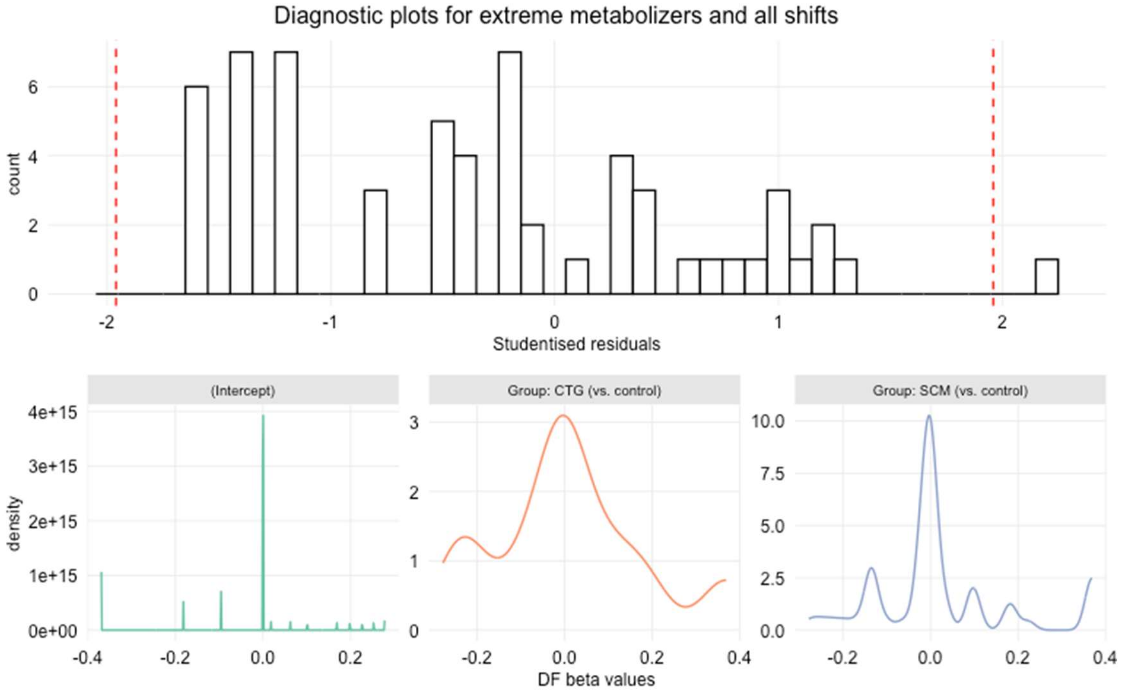

Non-extreme metabolizers

Then, fit the same model using non-extreme metabolizers only.

```
neg_binom_shifts(filter(df, extr_metabolizer == "no"), "non-extreme metabolizers")

## > Base model:
## # A tibble: 3 x 4
```

```
##   term                       estimate conf.low conf.high
##   <chr>                      <chr>    <chr>    <chr>
## 1 (Intercept)                2.22     1.60     3.16
## 2 Group: CTG (vs. control) 0.42     -0.77    1.71
## 3 Group: SCM (vs. control) -0.54    -1.60    0.43
## Dispersion parameter (std. error): 0.48 (0.06)
##
## > Comparing null and base models, log-likelihood test:
## Likelihood ratio tests of Negative Binomial Models
##
## Response: n_shifts_all
##   Model     theta Resid. df    2 x log-lik.   Test    df LR stat.   Pr(Chi)
## 1     1 0.4675777       249       -958.4856
## 2 group 0.4785403       247       -955.2591 1 vs 2     2 3.226439 0.1992451
##
## > Comparing null and base models, AIC and BIC:
##                AIC         BIC
## mod_null 962.4855615 969.528483
## mod_base 963.2591227 977.344966
## diff       0.7735611   7.816483
```

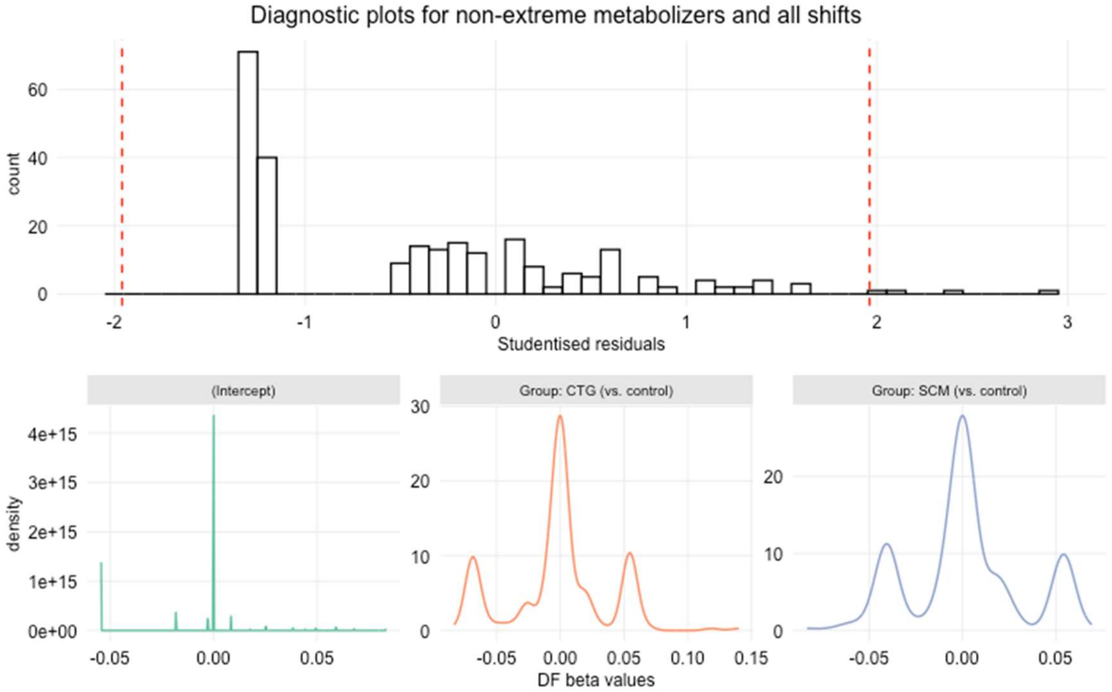

Binomial model for dichotomised drug changes

There are very few non-zero counts for drug changes, yielding quite few observations on which the fitting process can base parameter estimates. Therefore, we dichotomise the outcome to 0 vs. ≥ 1 changes. As above, we define a helper function that fits the model and returns results with diagnostics, including plots.

```
binomial_shifts <- function(dft) {
  cat("> Contingency table, ascertain sufficient observations")
  print(ct(dft$bin_drug_shifts, pretty[as.character(dft$group)]))

  mod_null <- glm(bin_drug_shifts ~ 1, data = dft, family = "binomial")
```

```
  mod_base <- update(mod_null, formula = . ~ . + group)

  cat("\n> Parameter estimates with confidence intervals:\n")
  print(tidy_res(mod_base, "OR"))
  logistic_pseudo_R2s(mod_base)

  cat("\n> Comparing null and base models: \n")
  anova_res <- anova(mod_null, mod_base)
  pval <- round(pchisq(anova_res$Deviance[2], anova_res$Df[2], lower.tail = FALSE), 3)
  print(cbind(anova_res, Pval = c(NA, pval))); cat("\n")

  matrix(c(AIC(mod_null, mod_base)$AIC, BIC(mod_null, mod_base)$BIC), nrow = 2,
         dimnames = list(c("mod_null", "mod_base"), c("AIC", "BIC"))) %>%
    addmargins(margin = 1, FUN = diff) %>%
    print()
}
```

All patients

Fit the binomial (logit-linked) model using data from all patients.

```
binomial_shifts(df)

## > Contingency table, ascertain sufficient observations
## ==========================================================================================
##          Control   CYP test-guided (CTG)   Structured clinical monitoring (SCM)   Total
## ------------------------------------------------------------------------------------------
## 0             92                      94                                     95     281
## row %         33                      33                                     34      90
## col %         87                      91                                     93
## ------------------------------------------------------------------------------------------
## 1             14                       9                                      7      30
## row %         47                      30                                     23      10
## col %         13                       9                                      7
## ------------------------------------------------------------------------------------------
## Total        106                     103                                    102     311
##               34                      33                                     33
## ==========================================================================================
##
## > Parameter estimates with confidence intervals:
## # A tibble: 3 x 4
##   term                      OR    conf.low conf.high
##   <chr>                     <chr> <chr>    <chr>
## 1 (Intercept)               0.15  0.08     0.26
## 2 Group: CTG (vs. control) 0.63  0.25     1.51
## 3 Group: SCM (vs. control) 0.48  0.18     1.22
##
## > Pseudo R^2 for logistic regression:
## Hosmer and Lemeshow   0.013
## Cox and Snell         0.008
## Nagelkerke            0.017
##
## > Comparing null and base models:
##   Resid. Df Resid. Dev Df Deviance  Pval
## 1       310   197.3240 NA       NA    NA
## 2       308   194.8262  2 2.497827 0.287
##
##                  AIC        BIC
```

© 2020 Jürgens G et al. *JAMA Network Open.*

```
## mod_null 199.324024 203.063817
## mod_base 200.826197 212.045576
## diff       1.502173   8.981759
```

Extreme metabolizers

Even with dichotomised outcome, we cannot fit in the extremes metabolizers due to sparsity. To avoid cluttering the notebook, function warnings from the following code chunk are hidden, but the the parameter estimate for the SCM group clearly reflects the problem.

```
binomial_shifts(filter(df, extr_metabolizer == "yes"))

## > Contingency table, ascertain sufficient observations
## =====================================================================================
##          Control   CYP test-guided (CTG)   Structured clinical monitoring (SCM)   Total
## -------------------------------------------------------------------------------------
## 0             19                      17                                     19      55
## row %         35                      31                                     35      90
## col %         90                      81                                    100
## -------------------------------------------------------------------------------------
## 1              2                       4                                      0       6
## row %         33                      67                                      0      10
## col %         10                      19                                      0
## -------------------------------------------------------------------------------------
## Total         21                      21                                     19      61
##               34                      34                                     31
## =====================================================================================
##
## > Parameter estimates with confidence intervals:
## # A tibble: 3 x 4
##   term                      OR    conf.low conf.high
##   <chr>                     <chr> <chr>    <chr>
## 1 (Intercept)               0.11  0.02     0.36
## 2 Group: CTG (vs. control) 2.24  0.39     17.63
## 3 Group: SCM (vs. control) 0.00  NA       NA
##
## > Pseudo R^2 for logistic regression:
## Hosmer and Lemeshow   0.142
## Cox and Snell         0.087
## Nagelkerke            0.184
##
## > Comparing null and base models:
##   Resid. Df Resid. Dev Df Deviance  Pval
## 1        60   39.21885 NA       NA    NA
## 2        58   33.65901  2 5.559841 0.062
##
##                 AIC       BIC
## mod_null 41.218847 43.329721
## mod_base 39.659006 45.991628
## diff     -1.559841  2.661907
```

Non-extreme metabolizers

```
binomial_shifts(filter(df, extr_metabolizer == "no"))

## > Contingency table, ascertain sufficient observations
## =====================================================================================
##          Control   CYP test-guided (CTG)   Structured clinical monitoring (SCM)   Total
## -------------------------------------------------------------------------------------
```

© 2020 Jürgens G et al. *JAMA Network Open.*

```
## 0            73                       77                                     76     226
## row %        32                       34                                     34      90
## col %        86                       94                                     92
## ---------------------------------------------------------------------------------------
## 1            12                        5                                      7      24
## row %        50                       21                                     29      10
## col %        14                        6                                      8
## ---------------------------------------------------------------------------------------
## Total        85                       82                                     83     250
##              34                       33                                     33
## =======================================================================================
##
## > Parameter estimates with confidence intervals:
## # A tibble: 3 x 4
##   term                      OR    conf.low conf.high
##   <chr>                     <chr> <chr>    <chr>
## 1 (Intercept)               0.16  0.08     0.29
## 2 Group: CTG (vs. control) 0.40  0.12     1.12
## 3 Group: SCM (vs. control) 0.56  0.20     1.47
##
## > Pseudo R^2 for logistic regression:
## Hosmer and Lemeshow   0.02
## Cox and Snell         0.013
## Nagelkerke            0.027
##
## > Comparing null and base models:
##   Resid. Df Resid. Dev Df Deviance Pval
## 1       249   158.1021 NA       NA   NA
## 2       247   154.8807  2 3.221306  0.2
##
##                  AIC        BIC
## mod_null 160.1020554 163.623516
## mod_base 160.8807491 171.445132
## diff       0.7786937   7.821616
```

O2b: Hallucinations and delusions

We start by looking at the data. Unfortunately, we have quite some missing values on these outcome variables.

```
apply(df[, c("hallucinations", "delusions")], 2, table, useNA = "ifany")

##      hallucinations delusions
## -4                7         4
## -3               11         6
## -2               12        20
## -1               20        25
## 0               131       107
## 1                30        35
## 2                24        33
## 3                 6        13
## 4                 2         2
## <NA>             68        66
```

It seems that slighly more patients were stable in terms of hallucinations than delusions.

```
na.exclude(df[, c("hallucinations", "delusions", "group")]) %>%
  gather(key, value, -group) %>%
  mutate(group = pretty[as.character(group)],
         key = pretty[key]) %>%
```

```r
    ggplot1(aes(x = value)) +
      geom_histogram(aes(colour = group, fill = group), alpha = 0.25, binwidth = 1) +
      geom_vline(xintercept = 0.5, linetype = 2, size = 0.4) +
      geom_label(x = -2.7, y = 30, label = "Non-improvement", size = geom_text_size) +
      geom_label(x = 2.7, y = 30, label = "Improvement", size = geom_text_size) +
      facet_grid(key ~ group, scales = "free_y") +
      guides(fill = FALSE, colour = FALSE) +
      labs(x = "Difference in SAPS score", y = "Number of patients") +
      scale_x_continuous(breaks = -4:4, minor_breaks = NULL)
```

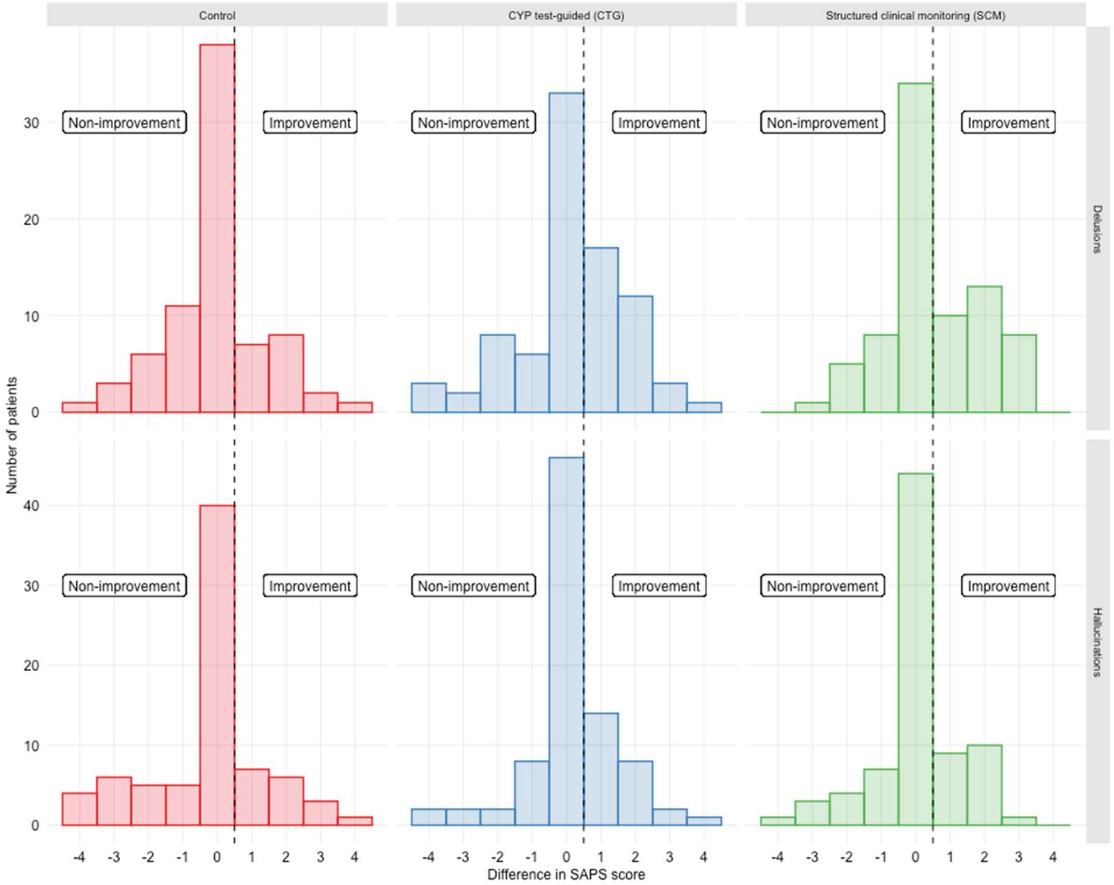

Tabulated of study group vs. dichotomised outcome variables for hallucinations and delusions

In this chunk we also show the tabulation.

```r
for (v in c("hallucinations", "delusions"))
  print(ct(df[, paste0(v, "_bin")], pretty[as.character(df$group)],
           dnn = c(first_up(v), "")))

##
## ==========================================================================================
## Hallucinations     Control   CYP test-guided (CTG)   Structured clinical monitoring (SCM)   Total
## ------------------------------------------------------------------------------------------
## not improved            61                      60                                     60     181
## row %                   34                      33                                     33      58
## col %                   58                      58                                     59
## ------------------------------------------------------------------------------------------
```

© 2020 Jürgens G et al. *JAMA Network Open.*

```
## improved                17                       25                                      20      62
## row %                   27                       40                                      32      20
## col %                   16                       24                                      20
## -------------------------------------------------------------------------------------------------
## NA                      28                       18                                      22      68
## row %                   41                       26                                      32      22
## col %                   26                       17                                      22
## -------------------------------------------------------------------------------------------------
## Total                  106                      103                                     102     311
##                         34                       33                                      33
## =================================================================================================
##
## ===============================================================================================
## Delusions       Control   CYP test-guided (CTG)   Structured clinical monitoring (SCM)   Total
## -----------------------------------------------------------------------------------------------
## not improved         59                      52                                     51     162
## row %                36                      32                                     31      52
## col %                56                      50                                     50
## -----------------------------------------------------------------------------------------------
## improved             18                      34                                     31      83
## row %                22                      41                                     37      27
## col %                17                      33                                     30
## -----------------------------------------------------------------------------------------------
## NA                   29                      17                                     20      66
## row %                44                      26                                     30      21
## col %                27                      17                                     20
## -----------------------------------------------------------------------------------------------
## Total               106                     103                                    102     311
##                      34                      33                                     33
## ===============================================================================================
```

Logistic models of SAPS

Then, we run the logistic regression analyses. Not surprisingly, age and illness duration covary somewhat, but not critically. Further, we confirm linearity of the logit (i.e., including an interaction between each numeric variable and its own log-transform). Continuous variables are arbitrarily cut into 6 intervals with approximately equal frequencies.

```
supp_3 <- list() # table 3 is a subset of supplemental 3

run_log_model <- function(v, local_df, s = "all patients") {
  preds <- c("group", "age", "gender", "illness_duration", "cyp2d6_dep_drug",
             "cyp2c19_dep_drug", "bl_uku")
  if (s == "all patients") preds <- c(preds, "poor_metabolizer", "fast_metabolizer")

  # Keep only complete cases
  local_df <- na.exclude(local_df[, c(paste0(v, "_bin"), preds)])

  cat("> Two-way tabulations between explanatory and outcome variables:")
  for (p in preds) {
      x <- local_df[, p]
      if (is.numeric(x)) x <- cut_number(x, 6)
      cat("\n", pretty[p], sep = "")
      print(ct(x, local_df[, paste0(v, "_bin")], prop.r = FALSE, prop.c = FALSE))
  }

  mod_full <- as.formula(paste0(v, "_bin ~", paste(preds, collapse = " + "))) %>%
    glm(data = local_df, family = "binomial", maxit = 100000)
```

© 2020 Jürgens G et al. *JAMA Network Open.*

```
  cat("\n> Coefficients with 95% confidence intervals:\n")
  print(tidy_res(mod_full, est = "OR"))
  logistic_pseudo_R2s(mod_full) # Pseuso-R^2s

  cat("\n> Comparing models:\n")
  print(anova(update(mod_full, formula = . ~ 1), mod_full, test = "Chisq"))

  cat("\n> Testing for multicollinearity (VIF) in full model:\n")
  print(vif(mod_full))

  cat("\n> Testing for linearity of the logit:\n")
  log_preds <- sprintf("I(%1$s * log(%1$s))", c("age", "illness_duration"))
  mod_full_expanded <- update(mod_full,
                              formula = as.formula(paste0(v, "_bin ~",
                                                          paste(c(preds, log_preds),
                                                                collapse = " + "))))
  print(tidy_res(mod_full_expanded, est = "OR", nice = FALSE))
  cat("\n")

  p1 <- ggplot() +
    geom_histogram(aes(x = rstudent(mod_full)), binwidth = 0.1, fill = "white",
                   colour = "black", size = 0.5) +
    geom_vline(aes(xintercept = c(-1.96, 1.96)), linetype = 2, colour = "red") +
    xlab("Studentised residuals")

  p2 <- data.frame(dfbeta(mod_full)[, 1:3]) %>%
    gather(predictor) %>%
    mutate(predictor = pretty[predictor]) %>%
    ggplot2(aes(x = value, y = ..density.., colour = predictor)) +
      geom_freqpoly(binwidth = 0.025) +
      xlab("DF beta values")

  exp_lev <- length(mod_full$coefficients) / nrow(local_df)
  p3 <- ggplot() +
    geom_histogram(aes(x = hatvalues(mod_full)), binwidth = 0.005, fill = "white",
                   colour = "black", size = 0.5) +
    geom_vline(aes(xintercept = c(1, 2, 3) * exp_lev),
               colour = c(grey(0.5), "orange", "red"), linetype = 2) +
    xlab("Leverage")

  grid.arrange(p1, p2, p3, ncol = 1, top = paste("Diagnostic plots for", v, "in", s))

  mutate(tidy_res(mod_full, est = "OR"), subset = first_up(s), outcome = first_up(v))
}
```

Hallucinations

All patients

```
supp_3$hallucinations_all <- run_log_model("hallucinations", df)

## > Two-way tabulations between explanatory and outcome variables:
## Study arm
## ========================================
##          not improved   improved   Total
## ----------------------------------------
## ctl                49         12      61
## ----------------------------------------
```

```
## ctg                45         22      67
## ----------------------------------------
## scm                51         16      67
## ----------------------------------------
## Total             145         50     195
## ========================================
##
## Age
## ==============================================
##                not improved   improved   Total
## ----------------------------------------------
## [18.7,26.9]              24          9      33
## ----------------------------------------------
## (26.9,32.1]              28          4      32
## ----------------------------------------------
## (32.1,37.9]              24          9      33
## ----------------------------------------------
## (37.9,44.8]              24          8      32
## ----------------------------------------------
## (44.8,53.3]              20         12      32
## ----------------------------------------------
## (53.3,72.7]              25          8      33
## ----------------------------------------------
## Total                   145         50     195
## ==============================================
##
## Gender
## =========================================
##           not improved   improved   Total
## -----------------------------------------
## female              69         18      87
## -----------------------------------------
## male                76         32     108
## -----------------------------------------
## Total              145         50     195
## =========================================
##
## Duration of illness
## ==============================================
##                not improved   improved   Total
## ----------------------------------------------
## [0.17,1.49]              21         12      33
## ----------------------------------------------
## (1.49,3.87]              27          5      32
## ----------------------------------------------
## (3.87,6.14]              28          5      33
## ----------------------------------------------
## (6.14,8.74]              22         10      32
## ----------------------------------------------
## (8.74,16.8]              25          7      32
## ----------------------------------------------
## (16.8,40.4]              22         11      33
## ----------------------------------------------
## Total                   145         50     195
## ==============================================
##
## CYP2D6-dependent drug use
## ========================================
##          not improved   improved   Total
```

© 2020 Jürgens G et al. *JAMA Network Open.*

```
## ----------------------------------------
## FALSE             33         13      46
## ----------------------------------------
## TRUE             112         37     149
## ----------------------------------------
## Total            145         50     195
## ========================================
##
## CYP2C19-dependent drug use
## ========================================
##          not improved   improved   Total
## ----------------------------------------
## FALSE            115         38     153
## ----------------------------------------
## TRUE              30         12      42
## ----------------------------------------
## Total            145         50     195
## ========================================
##
## UKU score at baseline
## ================================================
##              not improved   improved   Total
## ------------------------------------------------
## [0,11]                29          9      38
## ------------------------------------------------
## (11,14.7]             19          8      27
## ------------------------------------------------
## (14.7,19]             29         10      39
## ------------------------------------------------
## (19,23]               24          7      31
## ------------------------------------------------
## (23,29]               24          5      29
## ------------------------------------------------
## (29,47]               20         11      31
## ------------------------------------------------
## Total                145         50     195
## ================================================
##
## NA
## ========================================
##          not improved   improved   Total
## ----------------------------------------
## no               123         44     167
## ----------------------------------------
## yes               22          6      28
## ----------------------------------------
## Total            145         50     195
## ========================================
##
## NA
## ========================================
##          not improved   improved   Total
## ----------------------------------------
## no               141         49     190
## ----------------------------------------
## yes                4          1       5
## ----------------------------------------
## Total            145         50     195
## ========================================
```

```
##
## > Coefficients with 95% confidence intervals:
## # A tibble: 11 x 4
##    term                        OR    conf.low conf.high
##    <chr>                       <chr> <chr>    <chr>
##  1 (Intercept)                 0.10  0.02     0.50
##  2 Group: CTG (vs. control)    2.20  0.97     5.20
##  3 Group: SCM (vs. control)    1.38  0.58     3.36
##  4 Age                         1.01  0.97     1.05
##  5 Male gender                 1.83  0.92     3.73
##  6 Duration of illness         1.00  0.95     1.05
##  7 CYP2D6-dependent drug use   0.84  0.39     1.85
##  8 CYP2C19-dependent drug use  1.27  0.56     2.79
##  9 UKU score at baseline       1.01  0.97     1.05
## 10 Poor metabolizer            0.67  0.23     1.73
## 11 Fast metabolizer            0.68  0.03     5.41
##
## > Pseudo R^2 for logistic regression:
## Hosmer and Lemeshow   0.035
## Cox and Snell         0.039
## Nagelkerke            0.058
##
## > Comparing models:
## Analysis of Deviance Table
##
## Model 1: hallucinations_bin ~ 1
## Model 2: hallucinations_bin ~ group + age + gender + illness_duration +
##     cyp2d6_dep_drug + cyp2c19_dep_drug + bl_uku + poor_metabolizer +
##     fast_metabolizer
##   Resid. Df Resid. Dev Df Deviance Pr(>Chi)
## 1       194     222.01
## 2       184     214.17 10   7.8456   0.6439
##
## > Testing for multicollinearity (VIF) in full model:
##                      GVIF Df GVIF^(1/(2*Df))
## group            1.052176  2        1.012796
## age              1.846539  1        1.358874
## gender           1.074345  1        1.036506
## illness_duration 1.679914  1        1.296115
## cyp2d6_dep_drug  1.040306  1        1.019954
## cyp2c19_dep_drug 1.051708  1        1.025528
## bl_uku           1.202733  1        1.096692
## poor_metabolizer 1.021733  1        1.010808
## fast_metabolizer 1.043335  1        1.021438
##
## > Testing for linearity of the logit:
## # A tibble: 13 x 4
##    term                                        OR    conf.low conf.high
##    <chr>                                       <chr> <chr>    <chr>
##  1 (Intercept)                                 0.00  0.00     10.46
##  2 groupctg                                    2.27  0.99     5.42
##  3 groupscm                                    1.40  0.59     3.41
##  4 age                                         1.61  0.59     4.94
##  5 gendermale                                  1.80  0.90     3.71
##  6 illness_duration                            0.78  0.54     1.12
##  7 cyp2d6_dep_drugTRUE                         0.83  0.39     1.85
##  8 cyp2c19_dep_drugTRUE                        1.24  0.54     2.73
##  9 bl_uku                                      1.01  0.97     1.05
## 10 poor_metabolizeryes                         0.65  0.21     1.73
```

© 2020 Jürgens G et al. *JAMA Network Open.*

```
## 11 fast_metabolizeryes                        0.60  0.03     4.74
## 12 I(age * log(age))                           0.91  0.71     1.12
## 13 I(illness_duration * log(illness_duration)) 1.07  0.97     1.19
```

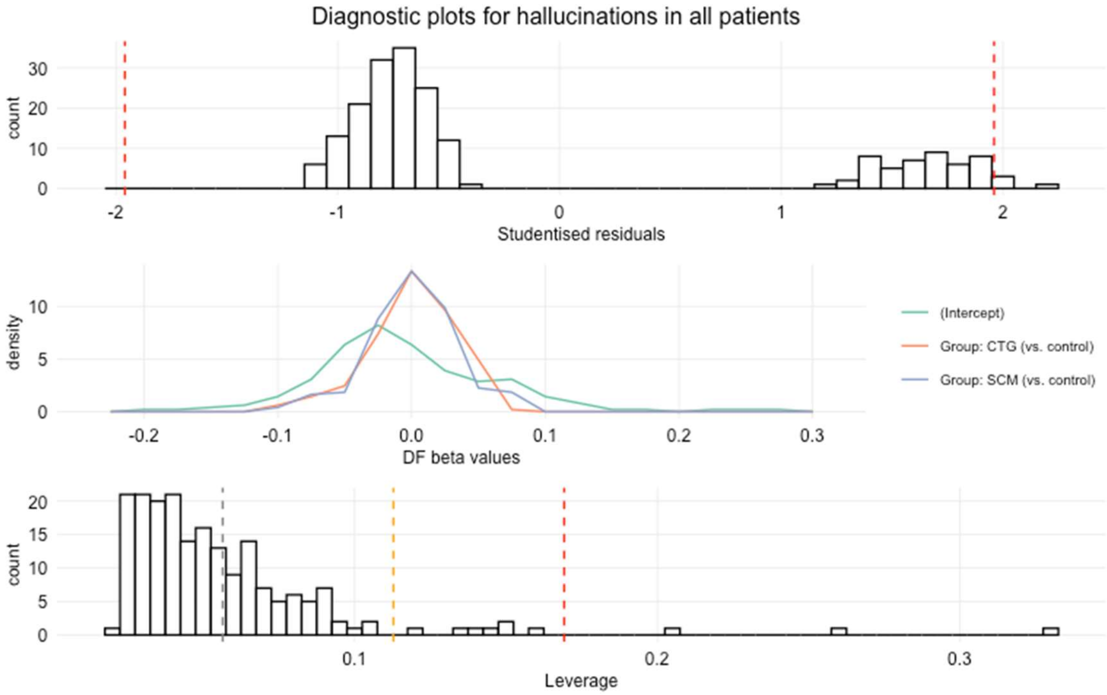

Non-extreme metabolizers

```
supp_3$hallucinations_non_extr <- run_log_model("hallucinations",
                                                filter(df, extr_metabolizer == "no"),
                                                "non-extremes metabolizers")

## > Two-way tabulations between explanatory and outcome variables:
## Study arm
## ========================================
##          not improved   improved   Total
## ----------------------------------------
## ctl                41         10      51
## ----------------------------------------
## ctg                35         19      54
## ----------------------------------------
## scm                43         14      57
## ----------------------------------------
## Total             119         43     162
## ========================================
##
## Age
## ==============================================
##                not improved   improved   Total
## ----------------------------------------------
## [18.7,27.1]              22          5      27
## ----------------------------------------------
## (27.1,32.6]              22          5      27
## ----------------------------------------------
## (32.6,38.5]              18          9      27
## ----------------------------------------------
```

© 2020 Jürgens G et al. *JAMA Network Open.*

```
## (38.5,45.1]              21          6      27
## ----------------------------------------------
## (45.1,51.8]              17         10      27
## ----------------------------------------------
## (51.8,72.7]              19          8      27
## ----------------------------------------------
## Total                   119         43     162
## ==============================================
##
## Gender
## ========================================
##           not improved   improved   Total
## ----------------------------------------
## female              57         18      75
## ----------------------------------------
## male                62         25      87
## ----------------------------------------
## Total              119         43     162
## ========================================
##
## Duration of illness
## ==============================================
##                not improved   improved   Total
## ----------------------------------------------
## [0.17,1.71]              16         11      27
## ----------------------------------------------
## (1.71,4.3]               25          3      28
## ----------------------------------------------
## (4.3,6.21]               22          4      26
## ----------------------------------------------
## (6.21,9.03]              20          7      27
## ----------------------------------------------
## (9.03,16.8]              20          8      28
## ----------------------------------------------
## (16.8,40.4]              16         10      26
## ----------------------------------------------
## Total                   119         43     162
## ==============================================
##
## CYP2D6-dependent drug use
## =======================================
##          not improved   improved   Total
## ---------------------------------------
## FALSE              27         11      38
## ---------------------------------------
## TRUE               92         32     124
## ---------------------------------------
## Total             119         43     162
## =======================================
##
## CYP2C19-dependent drug use
## =======================================
##          not improved   improved   Total
## ---------------------------------------
## FALSE              95         31     126
## ---------------------------------------
## TRUE               24         12      36
## ---------------------------------------
## Total             119         43     162
```

© 2020 Jürgens G et al. *JAMA Network Open.*

```
## ========================================
##
## UKU score at baseline
## ============================================
##              not improved   improved   Total
## --------------------------------------------
## [0,10.8]               21          6      27
## --------------------------------------------
## (10.8,14]              22          7      29
## --------------------------------------------
## (14,19]                23          8      31
## --------------------------------------------
## (19,23]                17          6      23
## --------------------------------------------
## (23,29.2]              20          5      25
## --------------------------------------------
## (29.2,47]              16         11      27
## --------------------------------------------
## Total                 119         43     162
## ============================================
##
## > Coefficients with 95% confidence intervals:
## # A tibble: 9 x 4
##   term                        OR    conf.low conf.high
##   <chr>                       <chr> <chr>    <chr>
## 1 (Intercept)                 0.09  0.01     0.52
## 2 Group: CTG (vs. control)    2.50  1.01     6.50
## 3 Group: SCM (vs. control)    1.43  0.56     3.78
## 4 Age                         1.01  0.97     1.05
## 5 Male gender                 1.48  0.71     3.17
## 6 Duration of illness         1.02  0.96     1.07
## 7 CYP2D6-dependent drug use   0.80  0.35     1.91
## 8 CYP2C19-dependent drug use 1.57  0.66     3.63
## 9 UKU score at baseline       1.02  0.98     1.06
##
## > Pseudo R^2 for logistic regression:
## Hosmer and Lemeshow   0.045
## Cox and Snell         0.051
## Nagelkerke            0.075
##
## > Comparing models:
## Analysis of Deviance Table
##
## Model 1: hallucinations_bin ~ 1
## Model 2: hallucinations_bin ~ group + age + gender + illness_duration +
##     cyp2d6_dep_drug + cyp2c19_dep_drug + bl_uku
##   Resid. Df Resid. Dev Df Deviance Pr(>Chi)
## 1       161     187.49
## 2       153     178.98  8   8.5086   0.3854
##
## > Testing for multicollinearity (VIF) in full model:
##                      GVIF Df GVIF^(1/(2*Df))
## group            1.061396  2        1.015008
## age              1.749200  1        1.322573
## gender           1.068379  1        1.033624
## illness_duration 1.574677  1        1.254862
## cyp2d6_dep_drug  1.038059  1        1.018852
## cyp2c19_dep_drug 1.057219  1        1.028211
## bl_uku           1.224952  1        1.106775
```

© 2020 Jürgens G et al. *JAMA Network Open.*

```
##
## > Testing for linearity of the logit:
## # A tibble: 11 x 4
##    term                                      OR    conf.low conf.high
##    <chr>                                     <chr> <chr>    <chr>
##  1 (Intercept)                               0.00  0.00     8.67
##  2 groupctg                                  2.57  1.03     6.75
##  3 groupscm                                  1.48  0.57     3.93
##  4 age                                       1.89  0.60     7.05
##  5 gendermale                                1.46  0.69     3.16
##  6 illness_duration                          0.74  0.48     1.13
##  7 cyp2d6_dep_drugTRUE                       0.74  0.32     1.79
##  8 cyp2c19_dep_drugTRUE                      1.46  0.60     3.43
##  9 bl_uku                                    1.02  0.97     1.06
## 10 I(age * log(age))                         0.88  0.66     1.12
## 11 I(illness_duration * log(illness_duration)) 1.09  0.97     1.23
```

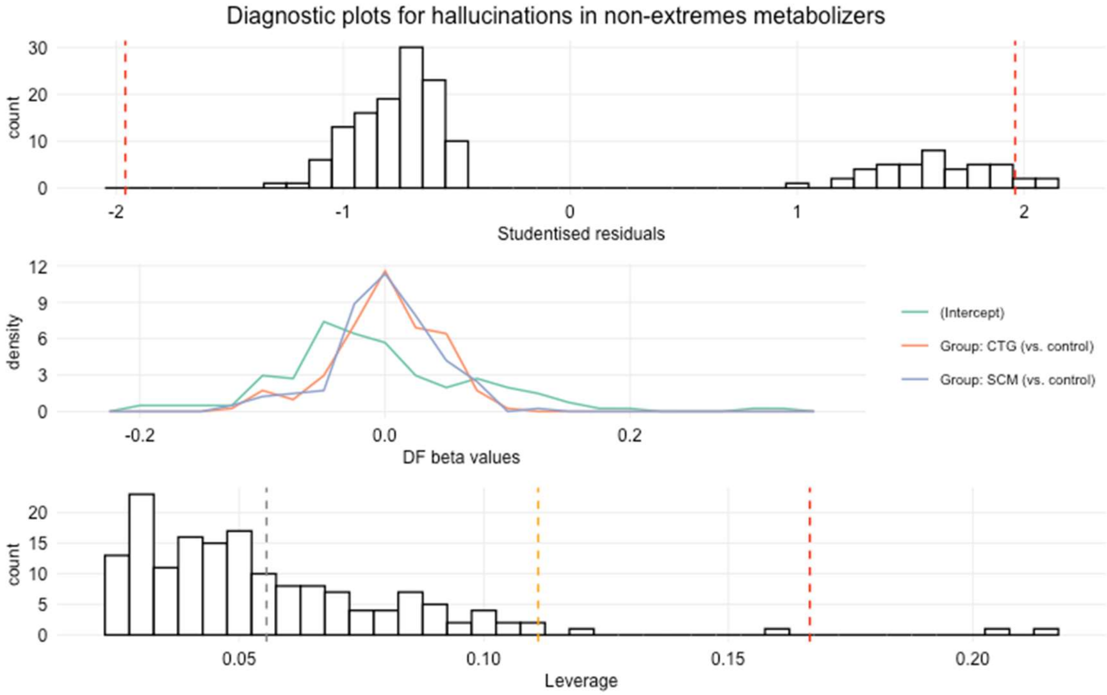

Extreme metabolizers

Clearly, there are issues due to data sparsity. We've hidden warnings from the function to avoid cluttering, but the parameter estimates leave little doubt anyway.

```
supp_3$hallucinations_extr <- run_log_model("hallucinations",
                                            filter(df, extr_metabolizer == "yes"),
                                            "extremes metabolizers")
```

```
## > Two-way tabulations between explanatory and outcome variables:
## Study arm
## ========================================
##          not improved   improved   Total
## ----------------------------------------
## ctl                 8          2      10
## ----------------------------------------
```

```
## ctg                10          3      13
## ----------------------------------------
## scm                 8          2      10
## ----------------------------------------
## Total              26          7      33
## ========================================
## 
## Age
## ==============================================
##                not improved   improved   Total
## ----------------------------------------------
## [20.4,26]                 3          3       6
## ----------------------------------------------
## (26,27.8]                 4          1       5
## ----------------------------------------------
## (27.8,32.7]               6          0       6
## ----------------------------------------------
## (32.7,42.8]               4          1       5
## ----------------------------------------------
## (42.8,57.5]               3          2       5
## ----------------------------------------------
## (57.5,68.2]               6          0       6
## ----------------------------------------------
## Total                    26          7      33
## ==============================================
## 
## Gender
## ========================================
##           not improved   improved   Total
## ----------------------------------------
## female             12          0      12
## ----------------------------------------
## male               14          7      21
## ----------------------------------------
## Total              26          7      33
## ========================================
## 
## Duration of illness
## ===============================================
##                 not improved   improved   Total
## -----------------------------------------------
## [0.27,0.643]               4          2       6
## -----------------------------------------------
## (0.643,1.44]               3          2       5
## -----------------------------------------------
## (1.44,5.51]                6          0       6
## -----------------------------------------------
## (5.51,7.28]                3          2       5
## -----------------------------------------------
## (7.28,11.5]                5          1       6
## -----------------------------------------------
## (11.5,37.5]                5          0       5
## -----------------------------------------------
## Total                     26          7      33
## ===============================================
## 
## CYP2D6-dependent drug use
## ========================================
##          not improved   improved   Total
```

© 2020 Jürgens G et al. *JAMA Network Open.*

```
## ----------------------------------------
## FALSE              6          2       8
## ----------------------------------------
## TRUE              20          5      25
## ----------------------------------------
## Total             26          7      33
## ========================================
##
## CYP2C19-dependent drug use
## ========================================
##          not improved   improved   Total
## ----------------------------------------
## FALSE             20          7      27
## ----------------------------------------
## TRUE               6          0       6
## ----------------------------------------
## Total             26          7      33
## ========================================
##
## UKU score at baseline
## ==============================================
##                not improved   improved   Total
## ----------------------------------------------
## [9,12.3]                  4          2       6
## ----------------------------------------------
## (12.3,15.7]               3          2       5
## ----------------------------------------------
## (15.7,19]                 4          2       6
## ----------------------------------------------
## (19,21]                   6          0       6
## ----------------------------------------------
## (21,24.7]                 3          1       4
## ----------------------------------------------
## (24.7,37]                 6          0       6
## ----------------------------------------------
## Total                    26          7      33
## ==============================================
##
## > Coefficients with 95% confidence intervals:
## # A tibble: 9 x 4
##   term                       OR    conf.low conf.high
##   <chr>                      <chr> <chr>    <chr>
## 1 (Intercept)                0.00  NA       NA
## 2 Group: CTG (vs. control)   0.55  0.01     12.72
## 3 Group: SCM (vs. control)   1.54  0.02     104.32
## 4 Age                        1.06  0.92     1.34
## 5 Male gender                NA    0.00     NA
## 6 Duration of illness        0.79  0.38     1.03
## 7 CYP2D6-dependent drug use  1.76  0.12     35.37
## 8 CYP2C19-dependent drug use 0.00  NA       NA
## 9 UKU score at baseline      0.77  0.45     0.99
##
## > Pseudo R^2 for logistic regression:
## Hosmer and Lemeshow   0.534
## Cox and Snell         0.424
## Nagelkerke            0.658
##
## > Comparing models:
## Analysis of Deviance Table
```

© 2020 Jürgens G et al. *JAMA Network Open.*

```
##
## Model 1: hallucinations_bin ~ 1
## Model 2: hallucinations_bin ~ group + age + gender + illness_duration +
##     cyp2d6_dep_drug + cyp2c19_dep_drug + bl_uku
##   Resid. Df Resid. Dev Df Deviance Pr(>Chi)
## 1        32     34.106
## 2        24     15.899  8   18.207  0.01973 *
## ---
## Signif. codes:  0 '***' 0.001 '**' 0.01 '*' 0.05 '.' 0.1 ' ' 1
##
## > Testing for multicollinearity (VIF) in full model:
##                      GVIF Df GVIF^(1/(2*Df))
## group            1.569530  2        1.119289
## age              2.341245  1        1.530113
## gender           1.000000  1        1.000000
## illness_duration 2.502782  1        1.582018
## cyp2d6_dep_drug  1.174890  1        1.083923
## cyp2c19_dep_drug 1.000000  1        1.000000
## bl_uku           1.592440  1        1.261919
##
## > Testing for linearity of the logit:
## # A tibble: 11 x 4
##    term                                       OR    conf.low conf.high
##    <chr>                                      <chr> <chr>    <chr>
##  1 (Intercept)                                NA    NA       NA
##  2 groupctg                                   2.29  0.03     649.07
##  3 groupscm                                   84.03 0.17     NA
##  4 age                                        0.01  0.00     2.54
##  5 gendermale                                 NA    0.00     NA
##  6 illness_duration                           9.96  2.41     NA
##  7 cyp2d6_dep_drugTRUE                        1.00  0.02     62.97
##  8 cyp2c19_dep_drugTRUE                       0.00  NA       NA
##  9 bl_uku                                     0.84  0.46     1.07
## 10 I(age * log(age))                          3.06  0.83     37.58
## 11 I(illness_duration * log(illness_duration)) 0.37  0.01     0.69
```

© 2020 Jürgens G et al. *JAMA Network Open.*

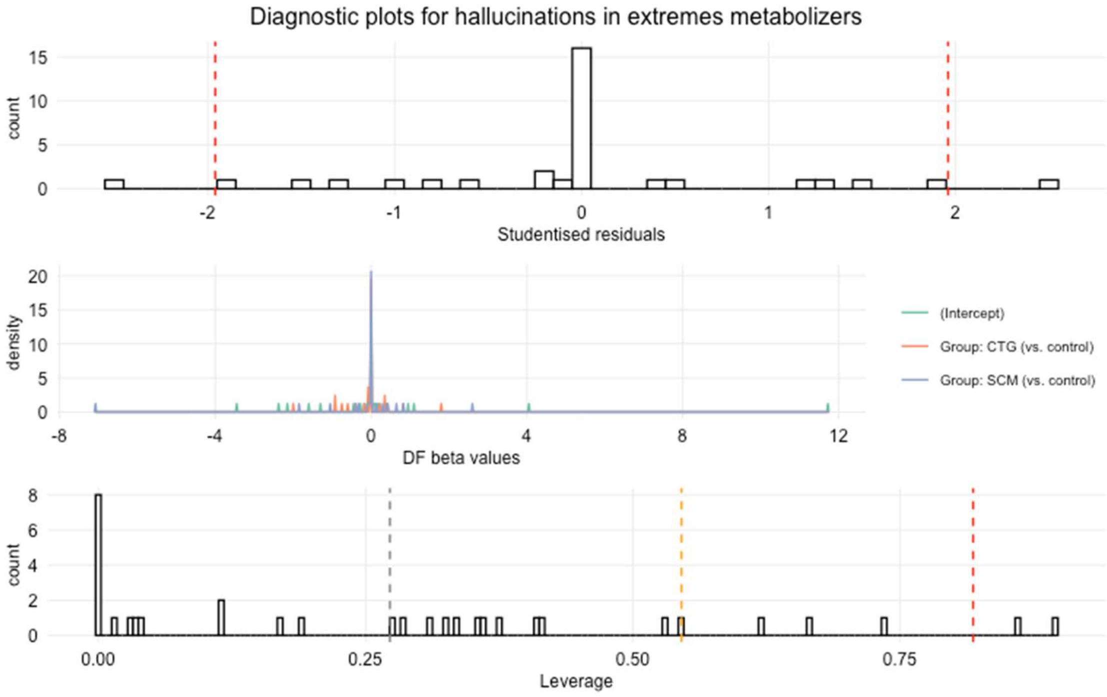

Delusions

All patients

```
supp_3$delusions_all <- run_log_model("delusions", df)

## > Two-way tabulations between explanatory and outcome variables:
## Study arm
## ========================================
##           not improved   improved   Total
## ----------------------------------------
## ctl                 46         15      61
## ----------------------------------------
## ctg                 40         28      68
## ----------------------------------------
## scm                 43         26      69
## ----------------------------------------
## Total              129         69     198
## ========================================
##
## Age
## ==============================================
##                 not improved   improved   Total
## ----------------------------------------------
## [18.7,27]                 18         15      33
## ----------------------------------------------
## (27,32.1]                 23         10      33
## ----------------------------------------------
## (32.1,37.9]               23         10      33
## ----------------------------------------------
## (37.9,45.1]               20         13      33
## ----------------------------------------------
## (45.1,53.6]               21         12      33
```

© 2020 Jürgens G et al. *JAMA Network Open.*

```
## ---------------------------------------------
## (53.6,72.7]              24          9      33
## ---------------------------------------------
## Total                   129         69     198
## =============================================
##
## Gender
## ========================================
##           not improved   improved   Total
## ----------------------------------------
## female              57         32      89
## ----------------------------------------
## male                72         37     109
## ----------------------------------------
## Total              129         69     198
## ========================================
##
## Duration of illness
## =============================================
##                not improved   improved   Total
## ---------------------------------------------
## [0.17,1.49]              20         14      34
## ---------------------------------------------
## (1.49,3.52]              20         12      32
## ---------------------------------------------
## (3.52,5.71]              25          8      33
## ---------------------------------------------
## (5.71,8.6]               24          9      33
## ---------------------------------------------
## (8.6,16.7]               19         14      33
## ---------------------------------------------
## (16.7,40.4]              21         12      33
## ---------------------------------------------
## Total                   129         69     198
## =============================================
##
## CYP2D6-dependent drug use
## =======================================
##          not improved   improved   Total
## ---------------------------------------
## FALSE              30         17      47
## ---------------------------------------
## TRUE               99         52     151
## ---------------------------------------
## Total             129         69     198
## =======================================
##
## CYP2C19-dependent drug use
## =======================================
##          not improved   improved   Total
## ---------------------------------------
## FALSE             100         56     156
## ---------------------------------------
## TRUE               29         13      42
## ---------------------------------------
## Total             129         69     198
## =======================================
##
## UKU score at baseline
```

```
## ============================================
##              not improved   improved   Total
## --------------------------------------------
## [0,10.8]               22         11      33
## --------------------------------------------
## (10.8,14]              27          7      34
## --------------------------------------------
## (14,19]                24         15      39
## --------------------------------------------
## (19,23]                20         11      31
## --------------------------------------------
## (23,29]                18         12      30
## --------------------------------------------
## (29,47]                18         13      31
## --------------------------------------------
## Total                 129         69     198
## ============================================
##
## NA
## ========================================
##          not improved   improved   Total
## ----------------------------------------
## no                115         56     171
## ----------------------------------------
## yes                14         13      27
## ----------------------------------------
## Total             129         69     198
## ========================================
##
## NA
## ========================================
##          not improved   improved   Total
## ----------------------------------------
## no                127         66     193
## ----------------------------------------
## yes                 2          3       5
## ----------------------------------------
## Total             129         69     198
## ========================================
##
## > Coefficients with 95% confidence intervals:
## # A tibble: 11 x 4
##    term                        OR    conf.low conf.high
##    <chr>                       <chr> <chr>    <chr>
##  1 (Intercept)                 0.46  0.10     2.05
##  2 Group: CTG (vs. control)    2.00  0.93     4.45
##  3 Group: SCM (vs. control)    1.78  0.81     4.01
##  4 Age                         0.97  0.94     1.01
##  5 Male gender                 0.90  0.48     1.69
##  6 Duration of illness         1.02  0.97     1.07
##  7 CYP2D6-dependent drug use   0.87  0.43     1.81
##  8 CYP2C19-dependent drug use  0.85  0.39     1.83
##  9 UKU score at baseline       1.03  1.00     1.07
## 10 Poor metabolizer            2.08  0.87     4.95
## 11 Fast metabolizer            2.81  0.43     22.62
##
## > Pseudo R^2 for logistic regression:
## Hosmer and Lemeshow   0.052
## Cox and Snell         0.065
```

© 2020 Jürgens G et al. *JAMA Network Open.*

```
## Nagelkerke            0.089
##
## > Comparing models:
## Analysis of Deviance Table
##
## Model 1: delusions_bin ~ 1
## Model 2: delusions_bin ~ group + age + gender + illness_duration + cyp2d6_dep_drug +
##     cyp2c19_dep_drug + bl_uku + poor_metabolizer + fast_metabolizer
##   Resid. Df Resid. Dev Df Deviance Pr(>Chi)
## 1       197     256.01
## 2       187     242.80 10   13.216   0.2118
##
## > Testing for multicollinearity (VIF) in full model:
##                      GVIF Df GVIF^(1/(2*Df))
## group            1.044086  2        1.010844
## age              1.938415  1        1.392270
## gender           1.062346  1        1.030702
## illness_duration 1.782398  1        1.335065
## cyp2d6_dep_drug  1.037702  1        1.018677
## cyp2c19_dep_drug 1.043278  1        1.021410
## bl_uku           1.195063  1        1.093189
## poor_metabolizer 1.033855  1        1.016786
## fast_metabolizer 1.054711  1        1.026991
##
## > Testing for linearity of the logit:
## # A tibble: 13 x 4
##    term                                      OR    conf.low conf.high
##    <chr>                                     <chr> <chr>    <chr>
##  1 (Intercept)                               1.63  0.00     NA
##  2 groupctg                                  2.03  0.94     4.54
##  3 groupscm                                  1.80  0.82     4.06
##  4 age                                       0.84  0.32     2.25
##  5 gendermale                                0.91  0.48     1.71
##  6 illness_duration                          0.98  0.70     1.39
##  7 cyp2d6_dep_drugTRUE                       0.89  0.43     1.85
##  8 cyp2c19_dep_drugTRUE                      0.86  0.39     1.83
##  9 bl_uku                                    1.03  1.00     1.07
## 10 poor_metabolizeryes                       1.97  0.81     4.80
## 11 fast_metabolizeryes                       2.62  0.39     21.57
## 12 I(age * log(age))                         1.03  0.84     1.26
## 13 I(illness_duration * log(illness_duration)) 1.01  0.92     1.11
```

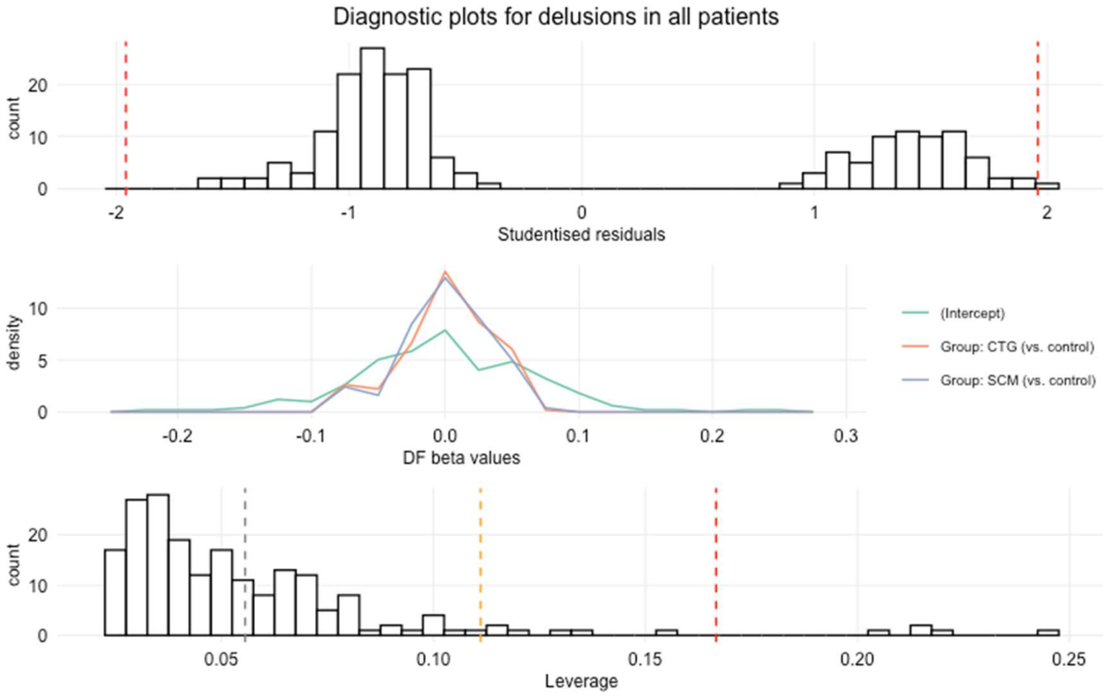

Non-extreme metabolizers

```
supp_3$delusions_non_extr <- run_log_model("delusions", filter(df, extr_metabolizer == "no"),
                                           "non-extremes metabolizers")

## > Two-way tabulations between explanatory and outcome variables:
## Study arm
## ========================================
##          not improved   improved   Total
## ----------------------------------------
## ctl                39         12      51
## ----------------------------------------
## ctg                34         21      55
## ----------------------------------------
## scm                40         20      60
## ----------------------------------------
## Total             113         53     166
## ========================================
##
## Age
## ==============================================
##                not improved   improved   Total
## ----------------------------------------------
## [18.7,27.2]              18         10      28
## ----------------------------------------------
## (27.2,32.7]              21          7      28
## ----------------------------------------------
## (32.7,38.5]              18          9      27
## ----------------------------------------------
## (38.5,45.5]              18         10      28
## ----------------------------------------------
## (45.5,52.1]              18          9      27
## ----------------------------------------------
## (52.1,72.7]              20          8      28
```

© 2020 Jürgens G et al. *JAMA Network Open.*

```
## ---------------------------------------------
## Total                  113         53     166
## =============================================
##
## Gender
## ========================================
##           not improved   improved   Total
## ----------------------------------------
## female              50         27      77
## ----------------------------------------
## male                63         26      89
## ----------------------------------------
## Total              113         53     166
## ========================================
##
## Duration of illness
## =============================================
##                not improved   improved   Total
## ---------------------------------------------
## [0.17,1.68]              20          8      28
## ---------------------------------------------
## (1.68,4.24]              18         10      28
## ---------------------------------------------
## (4.24,6.01]              22          5      27
## ---------------------------------------------
## (6.01,8.89]              21          7      28
## ---------------------------------------------
## (8.89,16.7]              14         13      27
## ---------------------------------------------
## (16.7,40.4]              18         10      28
## ---------------------------------------------
## Total                   113         53     166
## =============================================
##
## CYP2D6-dependent drug use
## =======================================
##          not improved   improved   Total
## ---------------------------------------
## FALSE              26         13      39
## ---------------------------------------
## TRUE               87         40     127
## ---------------------------------------
## Total             113         53     166
## =======================================
##
## CYP2C19-dependent drug use
## =======================================
##          not improved   improved   Total
## ---------------------------------------
## FALSE              88         41     129
## ---------------------------------------
## TRUE               25         12      37
## ---------------------------------------
## Total             113         53     166
## =======================================
##
## UKU score at baseline
## =========================================
##            not improved   improved   Total
```

```
## ----------------------------------------
## [0,10]              20         9      29
## ----------------------------------------
## (10,14]             25         4      29
## ----------------------------------------
## (14,19]             21        10      31
## ----------------------------------------
## (19,23]             16         7      23
## ----------------------------------------
## (23,29]             16        11      27
## ----------------------------------------
## (29,47]             15        12      27
## ----------------------------------------
## Total              113        53     166
## ========================================
##
## > Coefficients with 95% confidence intervals:
## # A tibble: 9 x 4
##   term                        OR    conf.low conf.high
##   <chr>                       <chr> <chr>    <chr>
## 1 (Intercept)                 0.46  0.08     2.39
## 2 Group: CTG (vs. control)    1.91  0.81     4.65
## 3 Group: SCM (vs. control)    1.47  0.62     3.55
## 4 Age                         0.97  0.93     1.01
## 5 Male gender                 0.81  0.41     1.62
## 6 Duration of illness         1.03  0.98     1.09
## 7 CYP2D6-dependent drug use   0.83  0.37     1.87
## 8 CYP2C19-dependent drug use 0.99  0.42     2.23
## 9 UKU score at baseline       1.04  1.00     1.08
##
## > Pseudo R^2 for logistic regression:
## Hosmer and Lemeshow   0.04
## Cox and Snell         0.049
## Nagelkerke            0.068
##
## > Comparing models:
## Analysis of Deviance Table
##
## Model 1: delusions_bin ~ 1
## Model 2: delusions_bin ~ group + age + gender + illness_duration + cyp2d6_dep_drug +
##     cyp2c19_dep_drug + bl_uku
##   Resid. Df Resid. Dev Df Deviance Pr(>Chi)
## 1       165     207.94
## 2       157     199.62  8   8.3197   0.4029
##
## > Testing for multicollinearity (VIF) in full model:
##                      GVIF Df GVIF^(1/(2*Df))
## group            1.031596  2        1.007807
## age              1.851072  1        1.360541
## gender           1.056111  1        1.027673
## illness_duration 1.643210  1        1.281878
## cyp2d6_dep_drug  1.036330  1        1.018003
## cyp2c19_dep_drug 1.051577  1        1.025464
## bl_uku           1.267520  1        1.125842
##
## > Testing for linearity of the logit:
## # A tibble: 11 x 4
##    term                                      OR    conf.low conf.high
##    <chr>                                     <chr> <chr>    <chr>
```

© 2020 Jürgens G et al. *JAMA Network Open.*

```
## 1 (Intercept)                            17.07 0.00     NA
## 2 groupctg                               1.87  0.79     4.56
## 3 groupscm                               1.43  0.60     3.48
## 4 age                                    0.58  0.20     1.77
## 5 gendermale                             0.82  0.41     1.66
## 6 illness_duration                       1.35  0.90     2.07
## 7 cyp2d6_dep_drugTRUE                    0.87  0.39     2.00
## 8 cyp2c19_dep_drugTRUE                   1.04  0.45     2.37
## 9 bl_uku                                 1.04  1.00     1.08
## 10 I(age * log(age))                     1.11  0.88     1.39
## 11 I(illness_duration * log(illness_duration)) 0.93  0.82     1.04
```

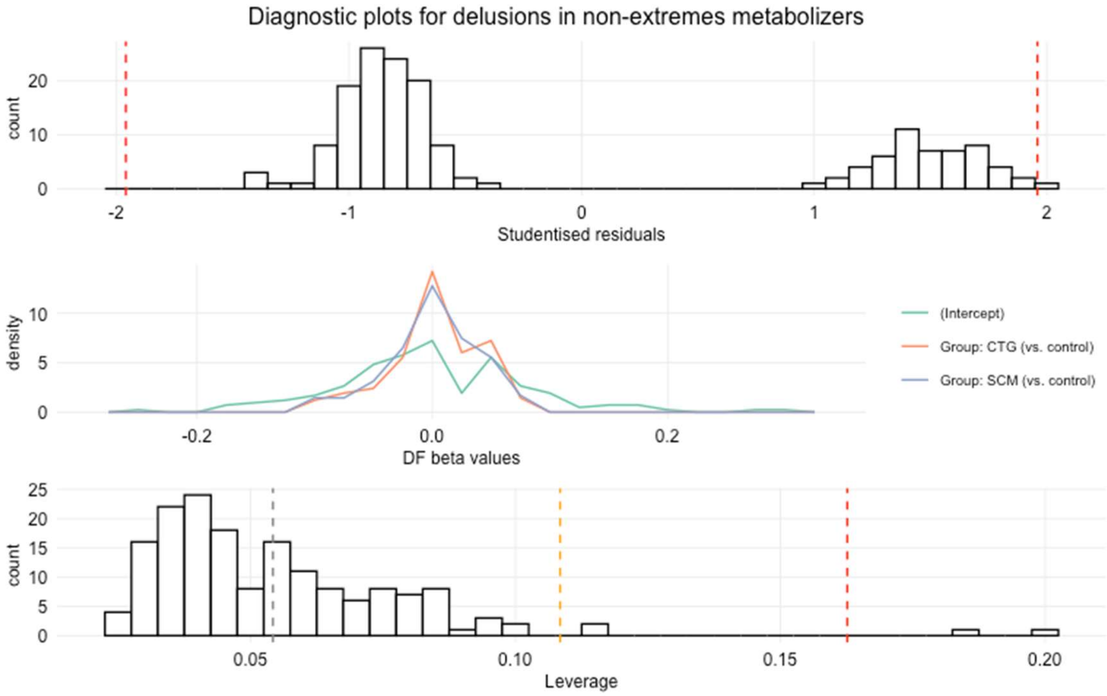

Extreme metabolizers

Again, there are issues due to data sparsity. We've hidden warnings from the function to avoid cluttering, but the parameter estimates leave little doubt.

```
supp_3$delusions_extr <- run_log_model("delusions", filter(df, extr_metabolizer == "yes"),
                                       "extremes metabolizers")

## > Two-way tabulations between explanatory and outcome variables:
## Study arm
## ========================================
##           not improved   improved   Total
## ----------------------------------------
## ctl                  7          3      10
## ----------------------------------------
## ctg                  6          7      13
## ----------------------------------------
## scm                  3          6       9
## ----------------------------------------
## Total               16         16      32
## ========================================
```

© 2020 Jürgens G et al. *JAMA Network Open.*

```
##
## Age
## ==============================================
##                 not improved   improved   Total
## ----------------------------------------------
## [20.4,26]                  2          4       6
## ----------------------------------------------
## (26,27.6]                  2          3       5
## ----------------------------------------------
## (27.6,32.5]                1          4       5
## ----------------------------------------------
## (32.5,41.1]                4          1       5
## ----------------------------------------------
## (41.1,58.2]                3          2       5
## ----------------------------------------------
## (58.2,68.2]                4          2       6
## ----------------------------------------------
## Total                     16         16      32
## ==============================================
##
## Gender
## =========================================
##            not improved   improved   Total
## -----------------------------------------
## female                7          5      12
## -----------------------------------------
## male                  9         11      20
## -----------------------------------------
## Total                16         16      32
## =========================================
##
## Duration of illness
## ===============================================
##                  not improved   improved   Total
## -----------------------------------------------
## [0.27,0.637]                0          6       6
## -----------------------------------------------
## (0.637,1.41]                2          3       5
## -----------------------------------------------
## (1.41,4.92]                 3          2       5
## -----------------------------------------------
## (4.92,6.74]                 3          2       5
## -----------------------------------------------
## (6.74,11.4]                 4          1       5
## -----------------------------------------------
## (11.4,37.5]                 4          2       6
## -----------------------------------------------
## Total                      16         16      32
## ===============================================
##
## CYP2D6-dependent drug use
## ========================================
##           not improved   improved   Total
## ----------------------------------------
## FALSE                4          4       8
## ----------------------------------------
## TRUE                12         12      24
## ----------------------------------------
## Total               16         16      32
```

© 2020 Jürgens G et al. *JAMA Network Open.*

```
## ========================================
##
## CYP2C19-dependent drug use
## ========================================
##          not improved   improved   Total
## ----------------------------------------
## FALSE              12         15      27
## ----------------------------------------
## TRUE                4          1       5
## ----------------------------------------
## Total              16         16      32
## ========================================
##
## UKU score at baseline
## ==============================================
##                not improved   improved   Total
## ----------------------------------------------
## [9,12.2]                  3          3       6
## ----------------------------------------------
## (12.2,15.3]               2          3       5
## ----------------------------------------------
## (15.3,19]                 2          4       6
## ----------------------------------------------
## (19,20.7]                 2          2       4
## ----------------------------------------------
## (20.7,24]                 4          2       6
## ----------------------------------------------
## (24,37]                   3          2       5
## ----------------------------------------------
## Total                    16         16      32
## ==============================================
##
## > Coefficients with 95% confidence intervals:
## # A tibble: 9 x 4
##   term                        OR    conf.low conf.high
##   <chr>                       <chr> <chr>    <chr>
## 1 (Intercept)                 4.78  0.07     464.97
## 2 Group: CTG (vs. control)    2.80  0.41     22.26
## 3 Group: SCM (vs. control)    8.58  0.95     128.58
## 4 Age                         0.96  0.87     1.06
## 5 Male gender                 1.66  0.27     11.36
## 6 Duration of illness         1.01  0.87     1.17
## 7 CYP2D6-dependent drug use   0.70  0.07     5.68
## 8 CYP2C19-dependent drug use 0.14  0.01     1.48
## 9 UKU score at baseline       0.95  0.85     1.07
##
## > Pseudo R^2 for logistic regression:
## Hosmer and Lemeshow   0.2
## Cox and Snell         0.242
## Nagelkerke            0.323
##
## > Comparing models:
## Analysis of Deviance Table
##
## Model 1: delusions_bin ~ 1
## Model 2: delusions_bin ~ group + age + gender + illness_duration + cyp2d6_dep_drug +
##     cyp2c19_dep_drug + bl_uku
##   Resid. Df Resid. Dev Df Deviance Pr(>Chi)
## 1        31     44.361
```

© 2020 Jürgens G et al. *JAMA Network Open.*

```
## 2        23     35.497  8   8.8641   0.3539
##
## > Testing for multicollinearity (VIF) in full model:
##                       GVIF Df GVIF^(1/(2*Df))
## group             1.316536  2        1.071169
## age               3.014477  1        1.736225
## gender            1.158855  1        1.076502
## illness_duration 3.224828  1        1.795781
## cyp2d6_dep_drug  1.252302  1        1.119063
## cyp2c19_dep_drug 1.141999  1        1.068643
## bl_uku            1.091242  1        1.044625
##
## > Testing for linearity of the logit:
## # A tibble: 11 x 4
##    term                                      OR    conf.low conf.high
##    <chr>                                     <chr> <chr>    <chr>
##  1 (Intercept)                               0.04  0.00     NA
##  2 groupctg                                  10.51 0.72     636.90
##  3 groupscm                                  9.07  0.40     621.30
##  4 age                                       2.08  0.06     116.58
##  5 gendermale                                1.07  0.11     9.80
##  6 illness_duration                          0.20  0.03     0.70
##  7 cyp2d6_dep_drugTRUE                       2.42  0.16     51.26
##  8 cyp2c19_dep_drugTRUE                      0.17  0.01     2.23
##  9 bl_uku                                    0.93  0.81     1.06
## 10 I(age * log(age))                         0.86  0.36     1.80
## 11 I(illness_duration * log(illness_duration)) 1.56  1.10     2.69
```

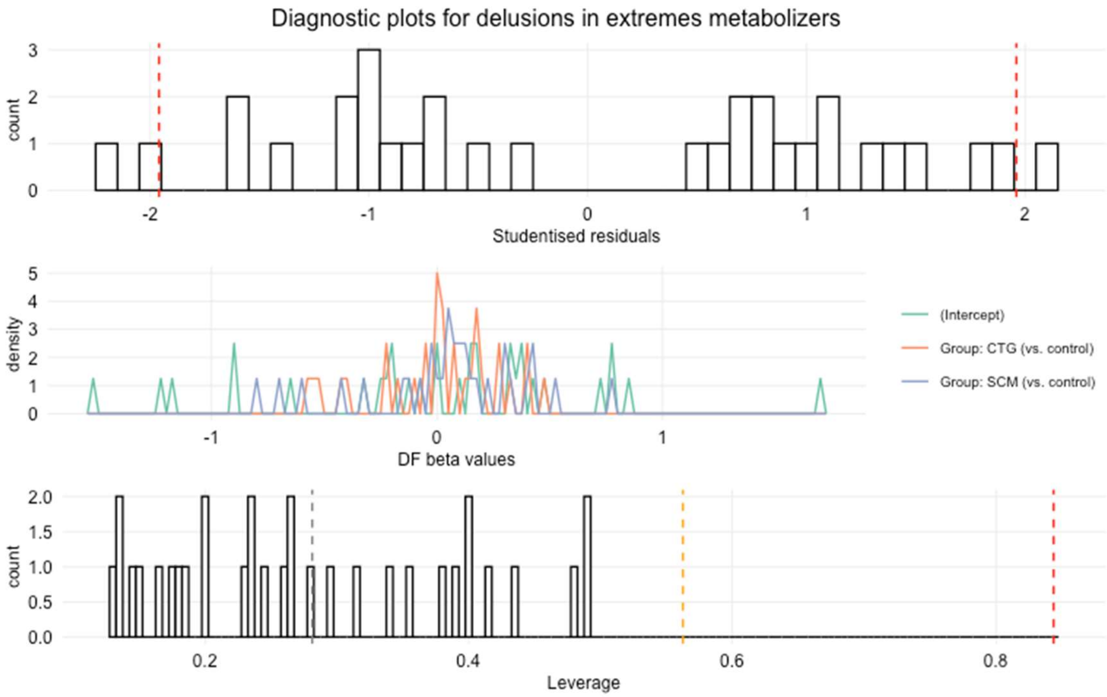

Table 3

Table 3 is a subset of the table in supplemental 3 (separate file).

© 2020 Jürgens G et al. *JAMA Network Open.*

```
supplemental_3 <- mutate(bind_rows(supp_3),
                         OR = sprintf("%s (%s-%s)", OR, conf.low, conf.high)) %>%
  select(outcome, subset, term, OR) %>%
  spread(outcome, OR) %>%
  setNames(sapply(names(.), first_up)) # cosmetics
save(supplemental_3, file = "supplemental_3.RData") # save as file; loaded in other file
```

O2c: Adverse drug reactions

As always, we start by looking at the data.

```
select(df, starts_with("uku_"), group) %>%
  na.exclude() %>%
  gather(key, value, -group) %>%
  mutate(group = pretty[as.character(group)],
         key = pretty[substring(key, 5)]) %>%
  ggplot1(aes(x = value)) +
    geom_histogram(aes(fill = group, colour = group), bins = 12, alpha = 0.25) +
    facet_grid(group ~ key, scales = "free") +
    guides(fill = FALSE, colour = FALSE) +
    labs(y = "Number of patients", x = "Difference in UKU score")
```

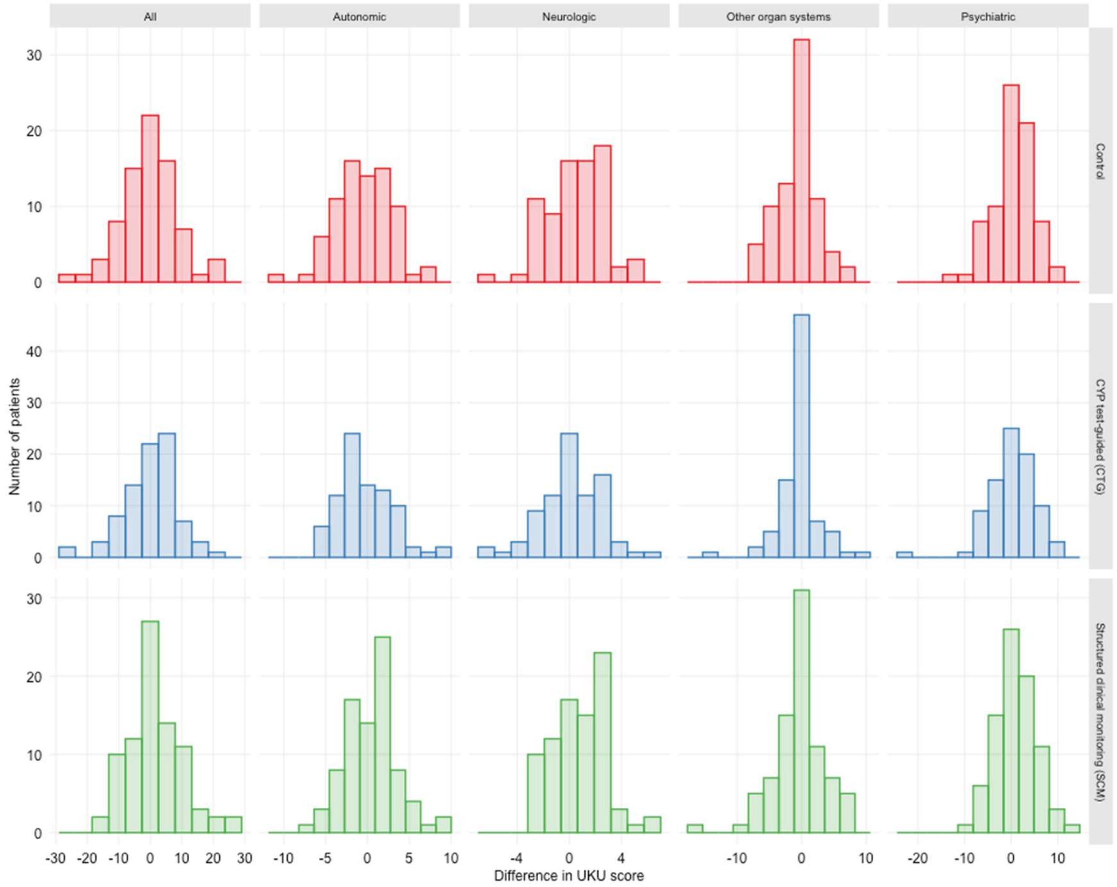

© 2020 Jürgens G et al. *JAMA Network Open.*

Linear models of UKU scores

As several times above, we define a helper function that we call repeatedly with slightly different information to produce appropriate results.

```
supp_4 <- list() # only part of this will make into the actual table 4 in the paper

# In this part, we assume the baseline numeric values of hallucinations and delusions
df <- mutate(df, bl_hallucinations_num = as.numeric(bl_hallucinations),
             bl_delusions_num = as.numeric(bl_delusions))

run_lin_model <- function(v, local_df, s = "all patients", include_interaction = TRUE) {
  vars <- c(paste0("uku_", v), "group", "age", "gender", "saei", "illness_duration",
            "cyp2d6_dep_drug", "cyp2c19_dep_drug", "bl_hallucinations_num",
            "bl_delusions_num", "poor_metabolizer", "fast_metabolizer")
  local_df <- na.exclude(local_df[, c(vars, "bl_hallucinations", "bl_delusions")])
  vars <- if (include_interaction) c(vars, "gender * poor_metabolizer", "gender * fast_metabolizer")
else vars[1:(length(vars) - 2)]
  vars <- if (s == "non-ultra-rapid metabolizers") grep("fast_metabolizer", vars, invert = TRUE, val
ue = TRUE) else vars
  mod <- lm(as.formula(paste0("uku_", v, " ~ ", paste(vars[-1], collapse = "+"))),
            data = local_df)

  cat("> Coefficients with 95% confidence intervals:\n")
  print(tidy_res(mod))

  cat("\n> Adjusted R^2 using Stern's equation: ") # from Discovering Stats with R (p. 273)
  n <- nrow(local_df)
  k <- length(vars) + include_interaction
  cat(1 - ((n - 1)/(n - k - 1) * (n - 2)/(n - k - 2)* (n + 1)/n) *
        (1 - summary(mod)$r.squared), "\n")

  cat("\n> Model comparison:\n")
  print(anova(update(mod, formula = . ~ 1), mod, test = "F"))

  cat("\n> Durbin-Watson Test for independence:\n")
  print(durbinWatsonTest(mod))
  cat("\n> Asserting the assumption of no multicollinearity:\n")
  print(vif(mod))

  h <- ggplot() +
    geom_histogram(aes(x = rstudent(mod), y = ..density..), binwidth = 0.5,
                   colour = "black", fill = "white") +
    stat_function(aes(x = rstudent(mod)), fun = dnorm, colour = "blue",
                  args = list(mean = mean(rstudent(mod)), sd = sd(rstudent(mod)))) +
    labs(x = "Studentised residuals", y = "Density")

  q <- qplot(sample = rstudent(mod)) +
    geom_abline(slope = 1, intercept = 0, colour = "blue") +
    labs(y = "Observed values", x = "Theoretical values")

  r <- ggplot() +
    geom_jitter(aes(x = mod$fitted.values, y = rstudent(mod)), alpha = 0.4, shape = 1) +
    geom_smooth(aes(x = mod$fitted.values, y = rstudent(mod)), method = "lm",
                colour = "blue") +
    labs(y = "Studentised residuals", x = "Fitted values")
```

© 2020 Jürgens G et al. *JAMA Network Open.*

```r
  grid.arrange(h, q, r, ncol = 3,
               top = paste0("Diagnostic plots for variable uku_", v, " in ", s))

  mutate(tidy_res(mod), subset = first_up(s), outcome = pretty[v])
}
```

Autonomic

All patients

```r
supp_4$aut_all <- run_lin_model("aut", df, "all patients")

## > Coefficients with 95% confidence intervals:
## # A tibble: 15 x 4
##    term                                estimate conf.low conf.high
##    <chr>                               <chr>    <chr>    <chr>
##  1 (Intercept)                         -0.92    -3.31    1.46
##  2 Group: CTG (vs. control)            0.28     -0.74    1.30
##  3 Group: SCM (vs. control)            0.58     -0.46    1.63
##  4 Age                                 -0.01    -0.06    0.04
##  5 Male gender                         0.55     -0.39    1.49
##  6 Special Assertive Early Intervention 0.30    -1.13    1.72
##  7 Duration of illness                 0.03     -0.03    0.09
##  8 CYP2D6-dependent drug use           0.77     -0.33    1.86
##  9 CYP2C19-dependent drug use          -0.45    -1.43    0.54
## 10 Hallucinations at baseline, numeric -0.23    -0.57    0.11
## 11 Delusions at baseline, numeric      0.14     -0.19    0.46
## 12 Poor metabolizer                    0.91     -0.79    2.60
## 13 Fast metabolizer                    1.18     -2.46    4.83
## 14 Poor metabolizer + male             -2.64    -4.91    -0.38
## 15 Fast metabolizer + male             -4.72    -9.56    0.13
##
## > Adjusted R^2 using Stern's equation: -0.06056598
##
## > Model comparison:
## Analysis of Variance Table
##
## Model 1: uku_aut ~ 1
## Model 2: uku_aut ~ group + age + gender + saei + illness_duration + cyp2d6_dep_drug +
##     cyp2c19_dep_drug + bl_hallucinations_num + bl_delusions_num +
##     poor_metabolizer + fast_metabolizer + gender * poor_metabolizer +
##     gender * fast_metabolizer
##   Res.Df    RSS Df Sum of Sq      F Pr(>F)
## 1    210 1987.4
## 2    196 1808.2 14    179.23 1.3877 0.1618
##
## > Durbin-Watson Test for independence:
##  lag Autocorrelation D-W Statistic p-value
##    1      0.09758675      1.804208   0.148
##  Alternative hypothesis: rho != 0
##
## > Asserting the assumption of no multicollinearity:
##                             GVIF Df GVIF^(1/(2*Df))
## group                   1.075250  2        1.018304
## age                     1.982288  1        1.407938
## gender                  1.290453  1        1.135981
## saei                    1.461880  1        1.209082
## illness_duration        1.671466  1        1.292852
## cyp2d6_dep_drug         1.019730  1        1.009817
```

© 2020 Jürgens G et al. *JAMA Network Open.*

```
## cyp2c19_dep_drug        1.035619  1        1.017654
## bl_hallucinations_num   1.475798  1        1.214824
## bl_delusions_num        1.508415  1        1.228176
## poor_metabolizer        2.345798  1        1.531600
## fast_metabolizer        2.505915  1        1.583008
## gender:poor_metabolizer 2.592019  1        1.609975
## gender:fast_metabolizer 2.569007  1        1.602812

## `geom_smooth()` using formula 'y ~ x'
```

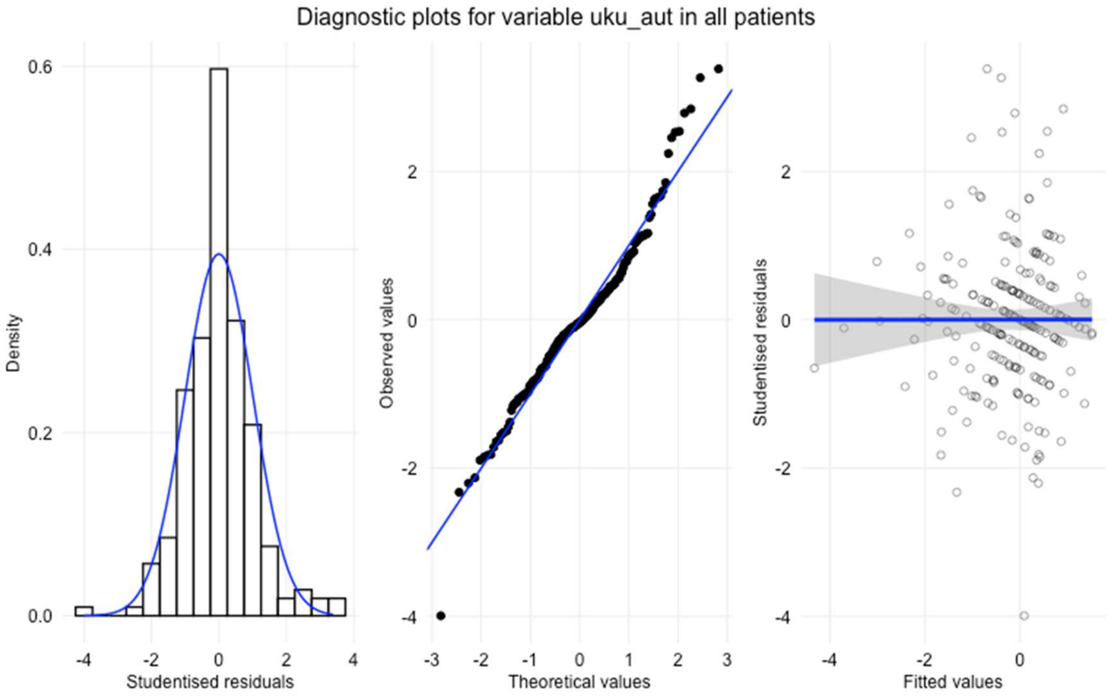

Non-extreme metabolizers

```
supp_4$aut_non_extr <- run_lin_model("aut", filter(df, extr_metabolizer == "no"),
                                     "non-extreme metabolizers", FALSE)

## > Coefficients with 95% confidence intervals:
## # A tibble: 11 x 4
##    term                                 estimate conf.low conf.high
##    <chr>                                <chr>    <chr>    <chr>
##  1 (Intercept)                          -1.54    -4.17    1.08
##  2 Group: CTG (vs. control)             0.06     -1.07    1.19
##  3 Group: SCM (vs. control)             1.03     -0.12    2.17
##  4 Age                                  0.01     -0.04    0.06
##  5 Male gender                          0.67     -0.26    1.60
##  6 Special Assertive Early Intervention -0.49    -2.09    1.11
##  7 Duration of illness                  -0.01    -0.08    0.06
##  8 CYP2D6-dependent drug use            0.61     -0.59    1.80
##  9 CYP2C19-dependent drug use           0.14     -0.92    1.20
## 10 Hallucinations at baseline, numeric  -0.16    -0.53    0.21
## 11 Delusions at baseline, numeric       0.13     -0.22    0.49
##
## > Adjusted R^2 using Stern's equation: -0.0784104
##
## > Model comparison:
```

© 2020 Jürgens G et al. *JAMA Network Open.*

```
## Analysis of Variance Table
##
## Model 1: uku_aut ~ 1
## Model 2: uku_aut ~ group + age + gender + saei + illness_duration + cyp2d6_dep_drug +
##     cyp2c19_dep_drug + bl_hallucinations_num + bl_delusions_num
##   Res.Df    RSS Df Sum of Sq      F Pr(>F)
## 1    168 1488.0
## 2    158 1410.4 10    77.556 0.8688 0.5637
##
## > Durbin-Watson Test for independence:
##  lag Autocorrelation D-W Statistic p-value
##    1      0.01779437      1.962193   0.774
##  Alternative hypothesis: rho != 0
##
## > Asserting the assumption of no multicollinearity:
##                           GVIF Df GVIF^(1/(2*Df))
## group                 1.077420  2        1.018817
## age                   1.829033  1        1.352417
## gender                1.048388  1        1.023908
## saei                  1.357753  1        1.165227
## illness_duration      1.619405  1        1.272558
## cyp2d6_dep_drug       1.009578  1        1.004777
## cyp2c19_dep_drug      1.033867  1        1.016792
## bl_hallucinations_num 1.440999  1        1.200416
## bl_delusions_num      1.471785  1        1.213171

## `geom_smooth()` using formula 'y ~ x'
```

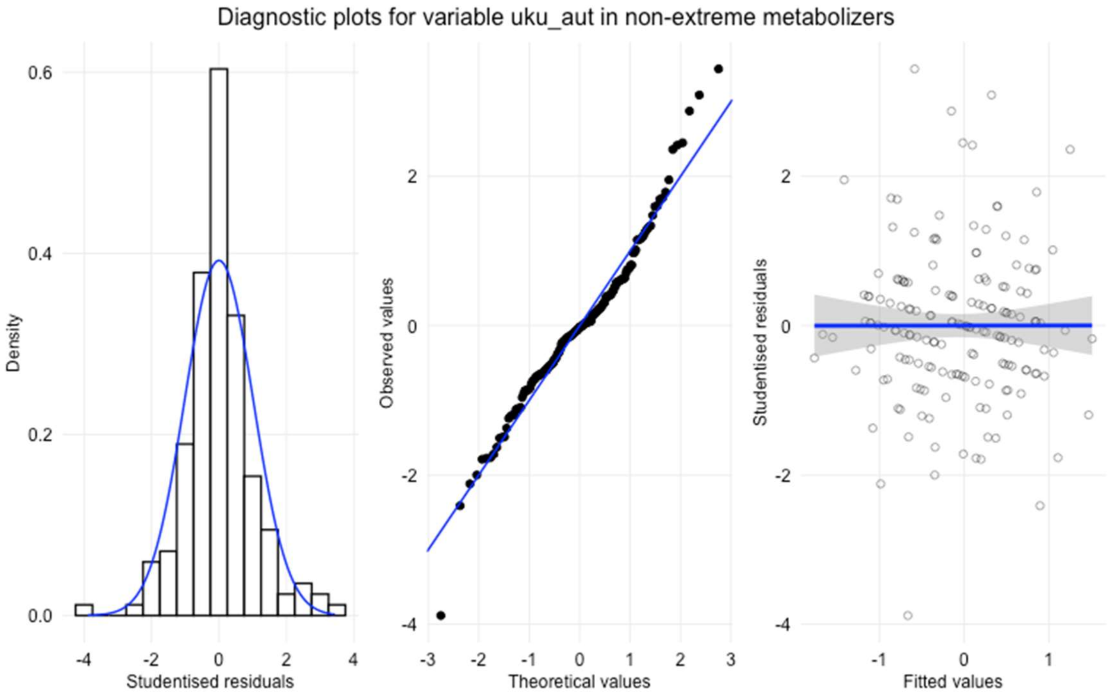

Extreme metabolizers

```
supp_4$aut_extr <- run_lin_model("aut", filter(df, extr_metabolizer == "yes"),
                                 "extreme metabolizers", FALSE)
```

© 2020 Jürgens G et al. *JAMA Network Open.*

```
## > Coefficients with 95% confidence intervals:
## # A tibble: 11 x 4
##    term                                estimate conf.low conf.high
##    <chr>                               <chr>    <chr>    <chr>
##  1 (Intercept)                         0.48     -4.93    5.90
##  2 Group: CTG (vs. control)            1.09     -1.19    3.37
##  3 Group: SCM (vs. control)            -0.37    -2.80    2.06
##  4 Age                                 -0.05    -0.15    0.05
##  5 Male gender                         -2.68    -4.60    -0.76
##  6 Special Assertive Early Intervention 1.86     -0.92    4.64
##  7 Duration of illness                 0.11     -0.03    0.25
##  8 CYP2D6-dependent drug use           1.40     -1.12    3.92
##  9 CYP2C19-dependent drug use          -2.86    -5.47    -0.25
## 10 Hallucinations at baseline, numeric  -0.34    -1.19    0.50
## 11 Delusions at baseline, numeric      0.30     -0.49    1.08
##
## > Adjusted R^2 using Stern's equation: 0.09734494
##
## > Model comparison:
## Analysis of Variance Table
##
## Model 1: uku_aut ~ 1
## Model 2: uku_aut ~ group + age + gender + saei + illness_duration + cyp2d6_dep_drug +
##     cyp2c19_dep_drug + bl_hallucinations_num + bl_delusions_num
##   Res.Df    RSS Df Sum of Sq      F   Pr(>F)
## 1     41 483.62
## 2     31 241.79 10    241.82 3.1004 0.007538 **
## ---
## Signif. codes:  0 '***' 0.001 '**' 0.01 '*' 0.05 '.' 0.1 ' ' 1
##
## > Durbin-Watson Test for independence:
##  lag Autocorrelation D-W Statistic p-value
##    1     -0.08803095      2.165014   0.574
##  Alternative hypothesis: rho != 0
##
## > Asserting the assumption of no multicollinearity:
##                           GVIF Df GVIF^(1/(2*Df))
## group                 1.449698  2        1.097285
## age                   2.602831  1        1.613329
## gender                1.169655  1        1.081506
## saei                  1.683502  1        1.297498
## illness_duration      2.210197  1        1.486673
## cyp2d6_dep_drug       1.143843  1        1.069506
## cyp2c19_dep_drug      1.226047  1        1.107270
## bl_hallucinations_num 2.096487  1        1.447925
## bl_delusions_num      1.868208  1        1.366824

## `geom_smooth()` using formula 'y ~ x'
```

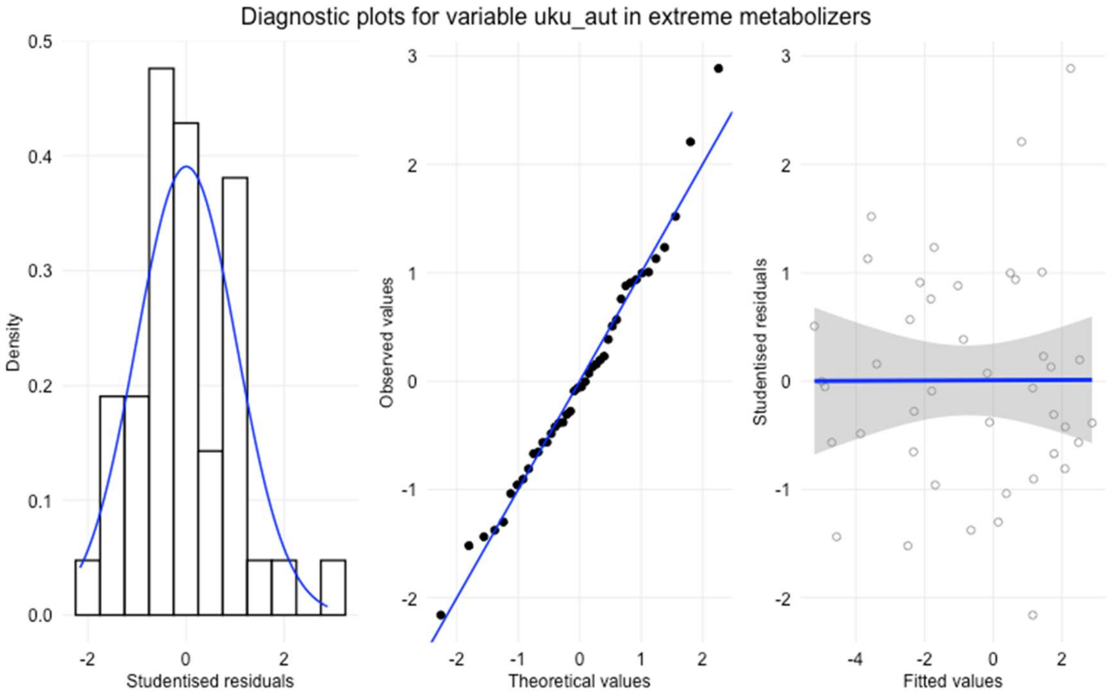

Neurological

All patients

```
supp_4$neu_all <- run_lin_model("neu", df, "all patients")

## > Coefficients with 95% confidence intervals:
## # A tibble: 15 x 4
##    term                                  estimate conf.low conf.high
##    <chr>                                 <chr>    <chr>    <chr>
##  1 (Intercept)                           -0.34    -2.13    1.45
##  2 Group: CTG (vs. control)              -0.46    -1.23    0.30
##  3 Group: SCM (vs. control)              0.36     -0.42    1.15
##  4 Age                                   0.01     -0.02    0.05
##  5 Male gender                           0.27     -0.43    0.98
##  6 Special Assertive Early Intervention  0.46     -0.61    1.53
##  7 Duration of illness                   -0.01    -0.05    0.04
##  8 CYP2D6-dependent drug use             0.25     -0.57    1.07
##  9 CYP2C19-dependent drug use            -0.27    -1.01    0.47
## 10 Hallucinations at baseline, numeric   0.26     0.01     0.52
## 11 Delusions at baseline, numeric        -0.18    -0.43    0.06
## 12 Poor metabolizer                      0.19     -1.09    1.46
## 13 Fast metabolizer                      -1.59    -4.32    1.15
## 14 Poor metabolizer + male               -0.39    -2.09    1.31
## 15 Fast metabolizer + male               1.78     -1.85    5.42
##
## > Adjusted R^2 using Stern's equation: -0.1016832
##
## > Model comparison:
## Analysis of Variance Table
##
## Model 1: uku_neu ~ 1
## Model 2: uku_neu ~ group + age + gender + saei + illness_duration + cyp2d6_dep_drug +
```

© 2020 Jürgens G et al. *JAMA Network Open.*

```
##     cyp2c19_dep_drug + bl_hallucinations_num + bl_delusions_num +
##     poor_metabolizer + fast_metabolizer + gender * poor_metabolizer +
##     gender * fast_metabolizer
##   Res.Df    RSS Df Sum of Sq      F Pr(>F)
## 1    210 1076.8
## 2    196 1017.6 14    59.122 0.8134 0.6541
##
## > Durbin-Watson Test for independence:
##  lag Autocorrelation D-W Statistic p-value
##    1      0.03879938       1.92238   0.598
##  Alternative hypothesis: rho != 0
##
## > Asserting the assumption of no multicollinearity:
##                             GVIF Df GVIF^(1/(2*Df))
## group                   1.075250  2        1.018304
## age                     1.982288  1        1.407938
## gender                  1.290453  1        1.135981
## saei                    1.461880  1        1.209082
## illness_duration        1.671466  1        1.292852
## cyp2d6_dep_drug         1.019730  1        1.009817
## cyp2c19_dep_drug        1.035619  1        1.017654
## bl_hallucinations_num   1.475798  1        1.214824
## bl_delusions_num        1.508415  1        1.228176
## poor_metabolizer        2.345798  1        1.531600
## fast_metabolizer        2.505915  1        1.583008
## gender:poor_metabolizer 2.592019  1        1.609975
## gender:fast_metabolizer 2.569007  1        1.602812

## `geom_smooth()` using formula 'y ~ x'
```

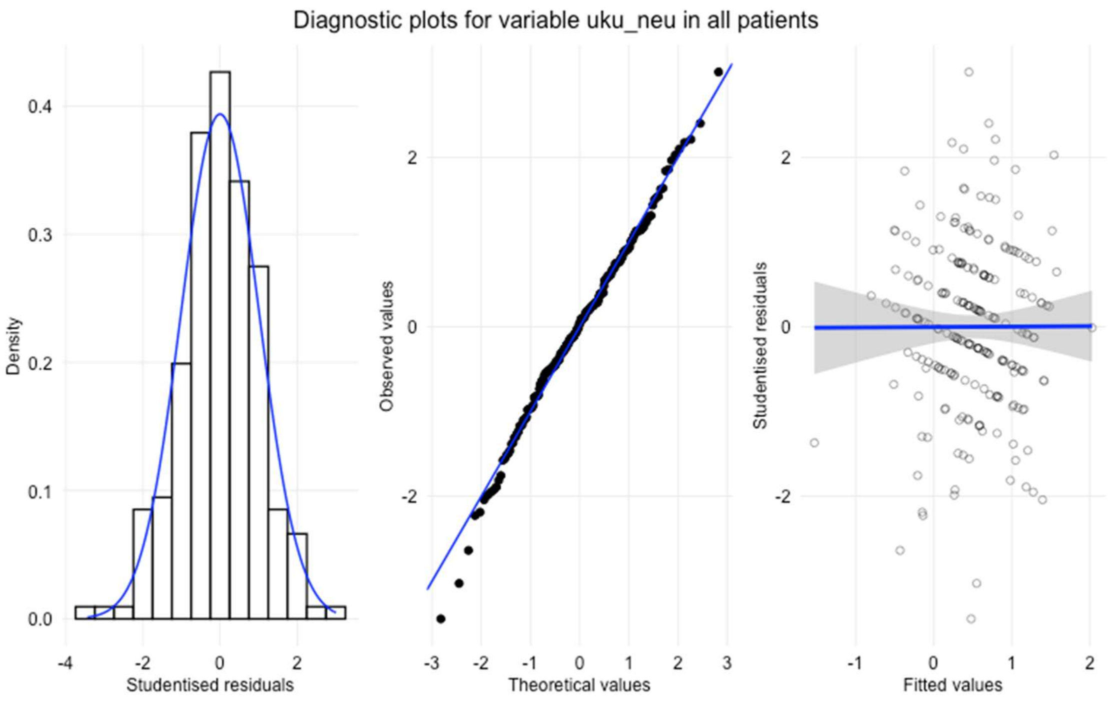

Non-extreme metabolizers

```
supp_4$neu_non_extr <- run_lin_model("neu", filter(df, extr_metabolizer == "no"),
                                     "non-extreme metabolizers", FALSE)
```

© 2020 Jürgens G et al. *JAMA Network Open.*

```
## > Coefficients with 95% confidence intervals:
## # A tibble: 11 x 4
##    term                               estimate conf.low conf.high
##    <chr>                              <chr>    <chr>    <chr>
##  1 (Intercept)                        -1.16    -3.13    0.80
##  2 Group: CTG (vs. control)           -0.16    -1.01    0.68
##  3 Group: SCM (vs. control)           0.58     -0.27    1.44
##  4 Age                                0.03     -0.01    0.07
##  5 Male gender                        0.37     -0.32    1.07
##  6 Special Assertive Early Intervention -0.16  -1.36    1.04
##  7 Duration of illness                -0.02    -0.07    0.03
##  8 CYP2D6-dependent drug use          0.44     -0.46    1.33
##  9 CYP2C19-dependent drug use         -0.17    -0.96    0.62
## 10 Hallucinations at baseline, numeric 0.28    0.01     0.56
## 11 Delusions at baseline, numeric     -0.18    -0.45    0.08
##
## > Adjusted R^2 using Stern's equation: -0.06408985
##
## > Model comparison:
## Analysis of Variance Table
##
## Model 1: uku_neu ~ 1
## Model 2: uku_neu ~ group + age + gender + saei + illness_duration + cyp2d6_dep_drug +
##     cyp2c19_dep_drug + bl_hallucinations_num + bl_delusions_num
##   Res.Df    RSS Df Sum of Sq      F Pr(>F)
## 1    168 842.21
## 2    158 787.71 10    54.498 1.0931 0.3706
##
## > Durbin-Watson Test for independence:
##  lag Autocorrelation D-W Statistic p-value
##    1       0.1106416      1.778278   0.108
##  Alternative hypothesis: rho != 0
##
## > Asserting the assumption of no multicollinearity:
##                           GVIF Df GVIF^(1/(2*Df))
## group                 1.077420  2        1.018817
## age                   1.829033  1        1.352417
## gender                1.048388  1        1.023908
## saei                  1.357753  1        1.165227
## illness_duration      1.619405  1        1.272558
## cyp2d6_dep_drug       1.009578  1        1.004777
## cyp2c19_dep_drug      1.033867  1        1.016792
## bl_hallucinations_num 1.440999  1        1.200416
## bl_delusions_num      1.471785  1        1.213171

## `geom_smooth()` using formula 'y ~ x'
```

© 2020 Jürgens G et al. *JAMA Network Open.*

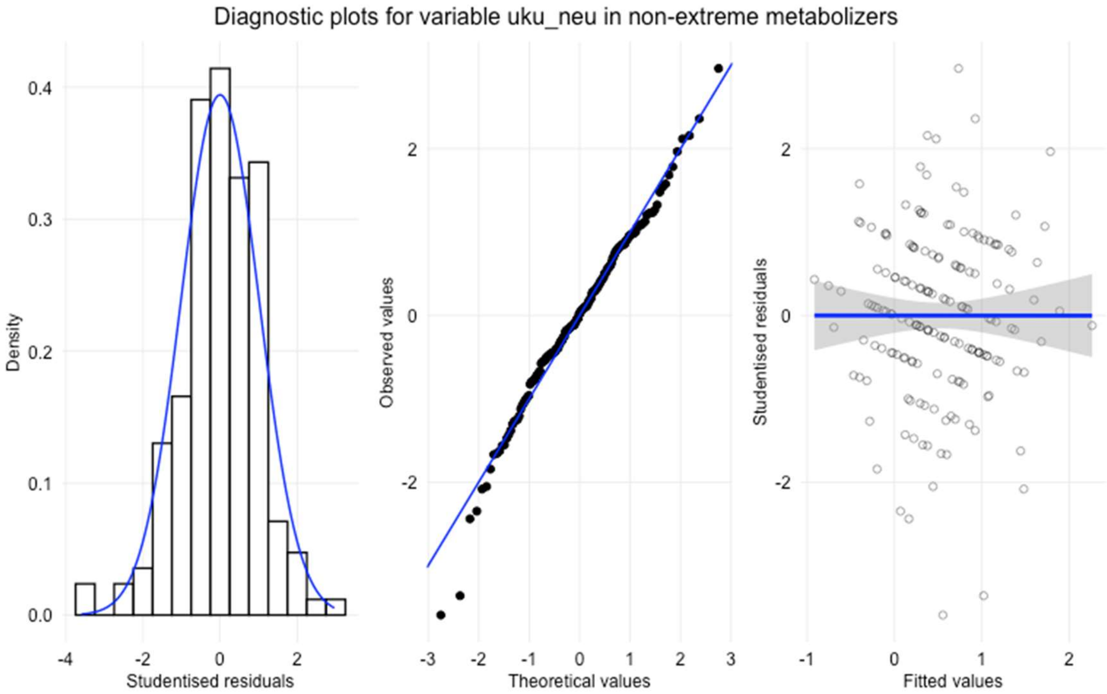

Extreme metabolizers

```
supp_4$neu_extr <- run_lin_model("neu", filter(df, extr_metabolizer == "yes"),
                                 "extreme metabolizers", FALSE)

## > Coefficients with 95% confidence intervals:
## # A tibble: 11 x 4
##    term                                estimate conf.low conf.high
##    <chr>                               <chr>    <chr>    <chr>
##  1 (Intercept)                         3.11     -1.51    7.74
##  2 Group: CTG (vs. control)            -1.91    -3.86    0.04
##  3 Group: SCM (vs. control)            -0.63    -2.70    1.45
##  4 Age                                 -0.04    -0.13    0.04
##  5 Male gender                         0.31     -1.33    1.95
##  6 Special Assertive Early Intervention 1.49    -0.89    3.86
##  7 Duration of illness                 0.03     -0.09    0.14
##  8 CYP2D6-dependent drug use           -0.27    -2.42    1.88
##  9 CYP2C19-dependent drug use          0.04     -2.19    2.27
## 10 Hallucinations at baseline, numeric -0.02    -0.74    0.70
## 11 Delusions at baseline, numeric      -0.17    -0.84    0.50
##
## > Adjusted R^2 using Stern's equation: -0.3580904
##
## > Model comparison:
## Analysis of Variance Table
##
## Model 1: uku_neu ~ 1
## Model 2: uku_neu ~ group + age + gender + saei + illness_duration + cyp2d6_dep_drug +
##     cyp2c19_dep_drug + bl_hallucinations_num + bl_delusions_num
##   Res.Df    RSS Df Sum of Sq      F Pr(>F)
## 1     41 234.41
## 2     31 176.32 10    58.079 1.0211 0.4488
##
## > Durbin-Watson Test for independence:
```

© 2020 Jürgens G et al. *JAMA Network Open.*

```
##  lag Autocorrelation D-W Statistic p-value
##    1      0.02135242      1.900875   0.734
##  Alternative hypothesis: rho != 0
##
## > Asserting the assumption of no multicollinearity:
##                           GVIF Df GVIF^(1/(2*Df))
## group                 1.449698  2        1.097285
## age                   2.602831  1        1.613329
## gender                1.169655  1        1.081506
## saei                  1.683502  1        1.297498
## illness_duration      2.210197  1        1.486673
## cyp2d6_dep_drug       1.143843  1        1.069506
## cyp2c19_dep_drug      1.226047  1        1.107270
## bl_hallucinations_num 2.096487  1        1.447925
## bl_delusions_num      1.868208  1        1.366824

## `geom_smooth()` using formula 'y ~ x'
```

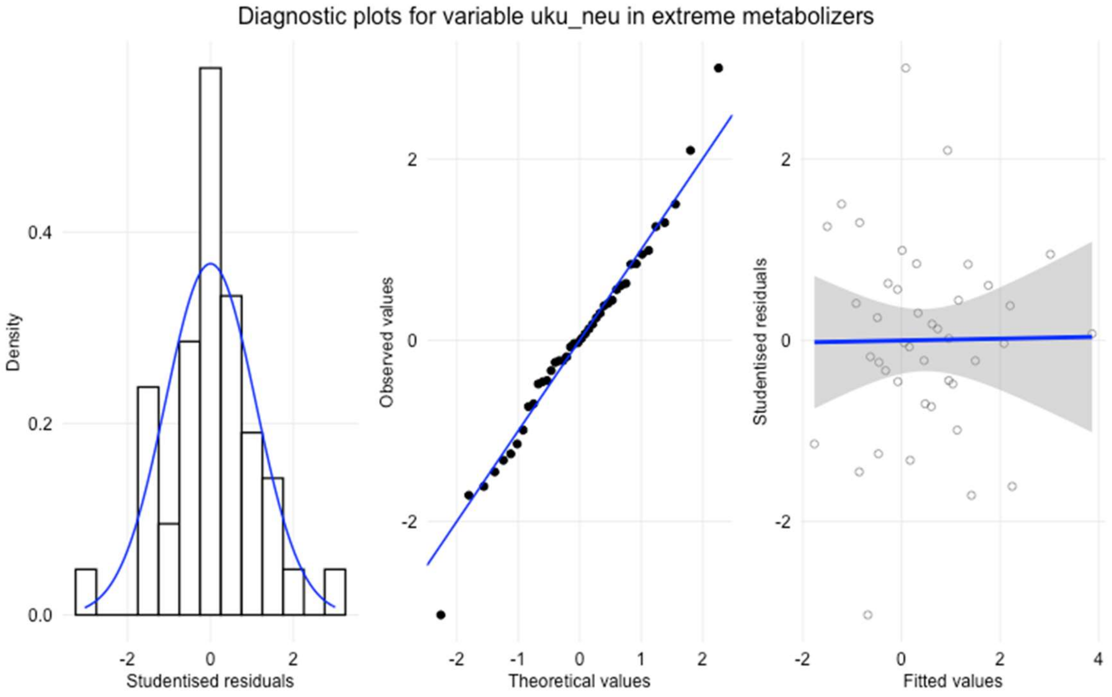

Psychiatric

All patients

```
supp_4$psy_all <- run_lin_model("psy", df, "all patients")

## > Coefficients with 95% confidence intervals:
## # A tibble: 15 x 4
##    term                                  estimate conf.low conf.high
##    <chr>                                 <chr>    <chr>    <chr>
##  1 (Intercept)                           1.38     -2.09    4.84
##  2 Group: CTG (vs. control)              0.40     -1.07    1.88
##  3 Group: SCM (vs. control)              0.64     -0.88    2.15
##  4 Age                                   -0.04    -0.11    0.03
##  5 Male gender                           0.31     -1.05    1.68
##  6 Special Assertive Early Intervention -0.27     -2.34    1.80
```

© 2020 Jürgens G et al. *JAMA Network Open.*

```
##  7 Duration of illness                 0.00     -0.09    0.09
##  8 CYP2D6-dependent drug use           0.03     -1.56    1.62
##  9 CYP2C19-dependent drug use          1.58     0.15     3.02
## 10 Hallucinations at baseline, numeric  0.13     -0.36    0.62
## 11 Delusions at baseline, numeric      -0.27    -0.74    0.20
## 12 Poor metabolizer                    1.04     -1.42    3.51
## 13 Fast metabolizer                    2.35     -2.93    7.64
## 14 Poor metabolizer + male             -0.27    -3.56    3.02
## 15 Fast metabolizer + male             -4.24    -11.27   2.79
##
## > Adjusted R^2 using Stern's equation: -0.1102825
##
## > Model comparison:
## Analysis of Variance Table
##
## Model 1: uku_psy ~ 1
## Model 2: uku_psy ~ group + age + gender + saei + illness_duration + cyp2d6_dep_drug +
##     cyp2c19_dep_drug + bl_hallucinations_num + bl_delusions_num +
##     poor_metabolizer + fast_metabolizer + gender * poor_metabolizer +
##     gender * fast_metabolizer
##   Res.Df    RSS Df Sum of Sq      F Pr(>F)
## 1    210 3993.3
## 2    196 3803.5 14    189.81 0.6986 0.7742
##
## > Durbin-Watson Test for independence:
##  lag Autocorrelation D-W Statistic p-value
##    1      -0.1141409      2.228108   0.102
##  Alternative hypothesis: rho != 0
##
## > Asserting the assumption of no multicollinearity:
##                             GVIF Df GVIF^(1/(2*Df))
## group                   1.075250  2        1.018304
## age                     1.982288  1        1.407938
## gender                  1.290453  1        1.135981
## saei                    1.461880  1        1.209082
## illness_duration        1.671466  1        1.292852
## cyp2d6_dep_drug         1.019730  1        1.009817
## cyp2c19_dep_drug        1.035619  1        1.017654
## bl_hallucinations_num   1.475798  1        1.214824
## bl_delusions_num        1.508415  1        1.228176
## poor_metabolizer        2.345798  1        1.531600
## fast_metabolizer        2.505915  1        1.583008
## gender:poor_metabolizer 2.592019  1        1.609975
## gender:fast_metabolizer 2.569007  1        1.602812

## `geom_smooth()` using formula 'y ~ x'
```

© 2020 Jürgens G et al. *JAMA Network Open.*

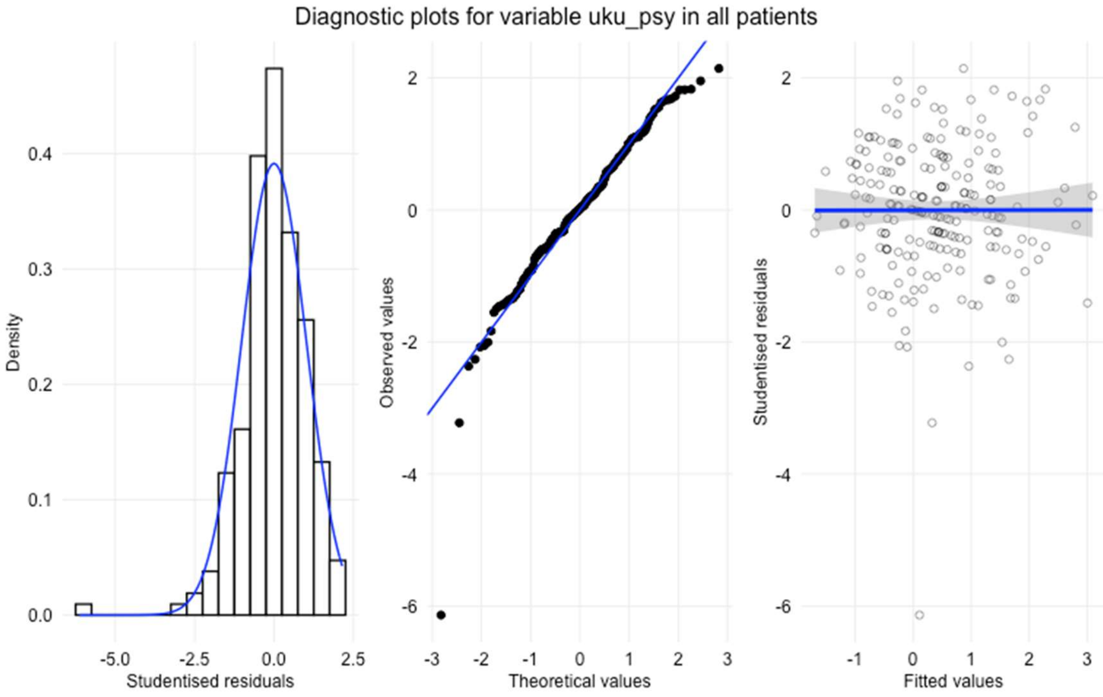

Non-extreme metabolizers

```
supp_4$psy_non_extr <- run_lin_model("psy", filter(df, extr_metabolizer == "no"),
                                     "non-extreme metabolizers", FALSE)

## > Coefficients with 95% confidence intervals:
## # A tibble: 11 x 4
##    term                                 estimate conf.low conf.high
##    <chr>                                <chr>    <chr>    <chr>
##  1 (Intercept)                          1.34     -2.55    5.23
##  2 Group: CTG (vs. control)             -0.29    -1.97    1.38
##  3 Group: SCM (vs. control)             0.30     -1.39    1.99
##  4 Age                                  -0.02    -0.10    0.05
##  5 Male gender                          0.24     -1.14    1.62
##  6 Special Assertive Early Intervention -0.05    -2.43    2.32
##  7 Duration of illness                  0.00     -0.10    0.11
##  8 CYP2D6-dependent drug use            -0.49    -2.26    1.28
##  9 CYP2C19-dependent drug use           1.50     -0.07    3.07
## 10 Hallucinations at baseline, numeric  0.13     -0.42    0.67
## 11 Delusions at baseline, numeric       -0.15    -0.67    0.38
##
## > Adjusted R^2 using Stern's equation: -0.1022367
##
## > Model comparison:
## Analysis of Variance Table
##
## Model 1: uku_psy ~ 1
## Model 2: uku_psy ~ group + age + gender + saei + illness_duration + cyp2d6_dep_drug +
##     cyp2c19_dep_drug + bl_hallucinations_num + bl_delusions_num
##   Res.Df    RSS Df Sum of Sq      F Pr(>F)
## 1    168 3191.6
## 2    158 3092.1 10     99.51 0.5085 0.8823
##
## > Durbin-Watson Test for independence:
```

© 2020 Jürgens G et al. *JAMA Network Open.*

```
##  lag Autocorrelation D-W Statistic p-value
##    1      -0.1345164      2.268522   0.098
##  Alternative hypothesis: rho != 0
##
## > Asserting the assumption of no multicollinearity:
##                          GVIF Df GVIF^(1/(2*Df))
## group                1.077420  2        1.018817
## age                  1.829033  1        1.352417
## gender               1.048388  1        1.023908
## saei                 1.357753  1        1.165227
## illness_duration     1.619405  1        1.272558
## cyp2d6_dep_drug      1.009578  1        1.004777
## cyp2c19_dep_drug     1.033867  1        1.016792
## bl_hallucinations_num 1.440999  1        1.200416
## bl_delusions_num     1.471785  1        1.213171

## `geom_smooth()` using formula 'y ~ x'
```

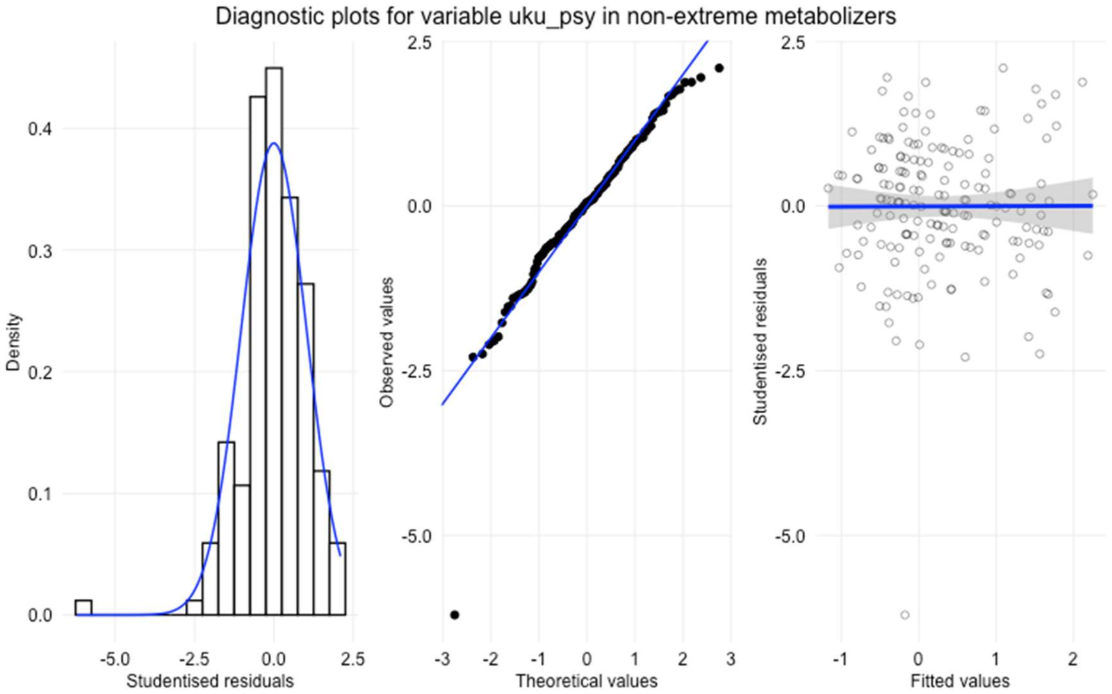

Extreme metabolizers

```
supp_4$psy_extr <- run_lin_model("psy", filter(df, extr_metabolizer == "yes"),
                                 "extreme metabolizers", FALSE)

## > Coefficients with 95% confidence intervals:
## # A tibble: 11 x 4
##    term                               estimate conf.low conf.high
##    <chr>                              <chr>    <chr>    <chr>
##  1 (Intercept)                        3.22     -5.63    12.06
##  2 Group: CTG (vs. control)           2.26     -1.47    5.98
##  3 Group: SCM (vs. control)           2.11     -1.85    6.08
##  4 Age                                -0.07    -0.23    0.09
##  5 Male gender                        -0.20    -3.34    2.93
##  6 Special Assertive Early Intervention -0.97  -5.50    3.57
##  7 Duration of illness                0.01     -0.22    0.23
```

© 2020 Jürgens G et al. *JAMA Network Open.*

```
##  8 CYP2D6-dependent drug use            1.17     -2.94    5.29
##  9 CYP2C19-dependent drug use           1.28     -2.98    5.54
## 10 Hallucinations at baseline, numeric  -0.14    -1.52    1.24
## 11 Delusions at baseline, numeric       -0.57    -1.85    0.72
## 
## > Adjusted R^2 using Stern's equation: -0.4717321
## 
## > Model comparison:
## Analysis of Variance Table
## 
## Model 1: uku_psy ~ 1
## Model 2: uku_psy ~ group + age + gender + saei + illness_duration + cyp2d6_dep_drug + 
##     cyp2c19_dep_drug + bl_hallucinations_num + bl_delusions_num
##   Res.Df    RSS Df Sum of Sq      F Pr(>F)
## 1     41 790.40                           
## 2     31 644.31 10    146.09 0.7029 0.7145
## 
## > Durbin-Watson Test for independence:
##  lag Autocorrelation D-W Statistic p-value
##    1     -0.07172491      2.075757   0.758
##  Alternative hypothesis: rho != 0
## 
## > Asserting the assumption of no multicollinearity:
##                           GVIF Df GVIF^(1/(2*Df))
## group                 1.449698  2        1.097285
## age                   2.602831  1        1.613329
## gender                1.169655  1        1.081506
## saei                  1.683502  1        1.297498
## illness_duration      2.210197  1        1.486673
## cyp2d6_dep_drug       1.143843  1        1.069506
## cyp2c19_dep_drug      1.226047  1        1.107270
## bl_hallucinations_num 2.096487  1        1.447925
## bl_delusions_num      1.868208  1        1.366824

## `geom_smooth()` using formula 'y ~ x'
```

© 2020 Jürgens G et al. *JAMA Network Open.*

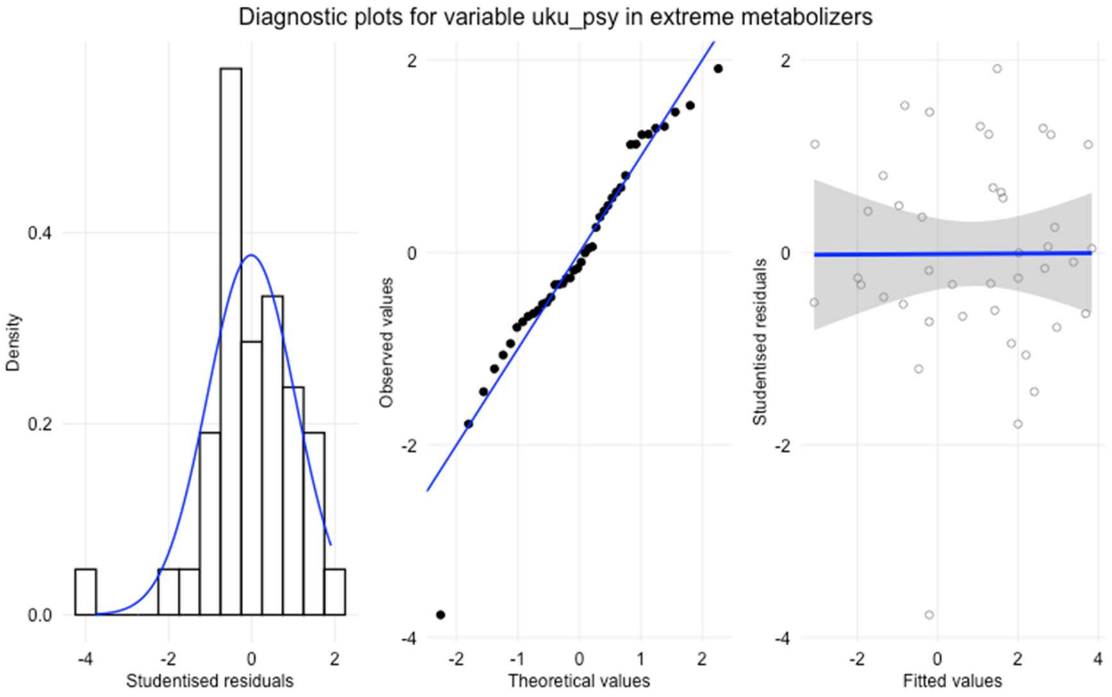

Other

All patients

```
supp_4$oth_all <- run_lin_model("oth", df, "all patients")

## > Coefficients with 95% confidence intervals:
## # A tibble: 15 x 4
##    term                                     estimate conf.low conf.high
##    <chr>                                    <chr>    <chr>    <chr>
##  1 (Intercept)                              0.25     -2.32    2.82
##  2 Group: CTG (vs. control)                 0.04     -1.07    1.14
##  3 Group: SCM (vs. control)                 0.03     -1.09    1.16
##  4 Age                                      -0.01    -0.07    0.04
##  5 Male gender                              -0.30    -1.31    0.72
##  6 Special Assertive Early Intervention -0.08    -1.64    1.48
##  7 Duration of illness                      0.05     -0.02    0.12
##  8 CYP2D6-dependent drug use                -1.09    -2.28    0.09
##  9 CYP2C19-dependent drug use               0.62     -0.45    1.68
## 10 Hallucinations at baseline, numeric  -0.07    -0.43    0.30
## 11 Delusions at baseline, numeric       0.19     -0.16    0.54
## 12 Poor metabolizer                         1.02     -0.81    2.84
## 13 Fast metabolizer                         -5.14    -9.06    -1.21
## 14 Poor metabolizer + male                  -1.60    -4.04    0.84
## 15 Fast metabolizer + male                  3.07     -2.15    8.29
##
## > Adjusted R^2 using Stern's equation: -0.04852641
##
## > Model comparison:
## Analysis of Variance Table
##
## Model 1: uku_oth ~ 1
## Model 2: uku_oth ~ group + age + gender + saei + illness_duration + cyp2d6_dep_drug +
```

© 2020 Jürgens G et al. *JAMA Network Open.*

```
##     cyp2c19_dep_drug + bl_hallucinations_num + bl_delusions_num +
##     poor_metabolizer + fast_metabolizer + gender * poor_metabolizer +
##     gender * fast_metabolizer
##   Res.Df    RSS Df Sum of Sq     F  Pr(>F)
## 1    208 2310.0
## 2    194 2074.6 14    235.36 1.572 0.08991 .
## ---
## Signif. codes:  0 '***' 0.001 '**' 0.01 '*' 0.05 '.' 0.1 ' ' 1
##
## > Durbin-Watson Test for independence:
##  lag Autocorrelation D-W Statistic p-value
##    1      0.09183531      1.803807   0.168
##  Alternative hypothesis: rho != 0
##
## > Asserting the assumption of no multicollinearity:
##                             GVIF Df GVIF^(1/(2*Df))
## group                   1.075606  2        1.018388
## age                     1.978612  1        1.406632
## gender                  1.293384  1        1.137271
## saei                    1.461560  1        1.208950
## illness_duration        1.663455  1        1.289750
## cyp2d6_dep_drug         1.020856  1        1.010374
## cyp2c19_dep_drug        1.035240  1        1.017467
## bl_hallucinations_num   1.483038  1        1.217800
## bl_delusions_num        1.511205  1        1.229311
## poor_metabolizer        2.341479  1        1.530189
## fast_metabolizer        2.509067  1        1.584003
## gender:poor_metabolizer 2.595187  1        1.610958
## gender:fast_metabolizer 2.570398  1        1.603246

## `geom_smooth()` using formula 'y ~ x'
```

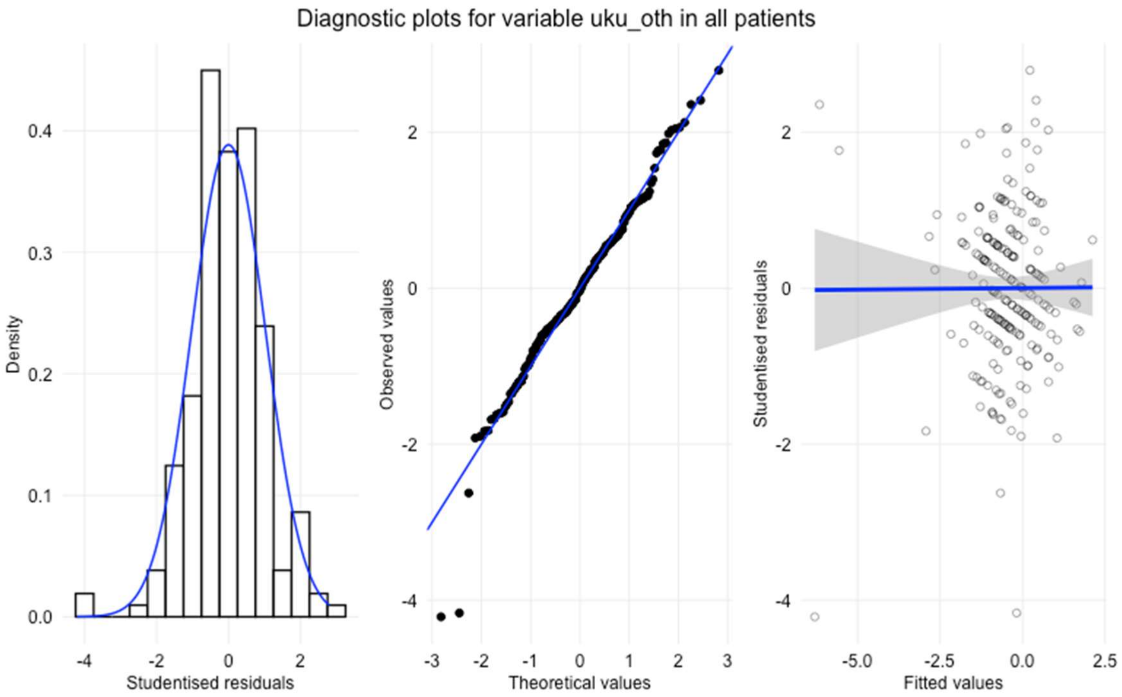

© 2020 Jürgens G et al. *JAMA Network Open.*

Non-extreme metabolizers

```
supp_4$oth_non_extr <- run_lin_model("oth", filter(df, extr_metabolizer == "no"),
                                     "non-extreme metabolizers", FALSE)

## > Coefficients with 95% confidence intervals:
## # A tibble: 11 x 4
##    term                                 estimate conf.low conf.high
##    <chr>                                <chr>    <chr>    <chr>    
##  1 (Intercept)                          -0.44    -3.27    2.39     
##  2 Group: CTG (vs. control)             -0.12    -1.35    1.10     
##  3 Group: SCM (vs. control)             0.26     -0.97    1.49     
##  4 Age                                  0.00     -0.05    0.06     
##  5 Male gender                          -0.24    -1.25    0.77     
##  6 Special Assertive Early Intervention -0.35    -2.11    1.41     
##  7 Duration of illness                  0.03     -0.04    0.11     
##  8 CYP2D6-dependent drug use            -0.92    -2.21    0.37     
##  9 CYP2C19-dependent drug use           0.86     -0.29    2.00     
## 10 Hallucinations at baseline, numeric  -0.20    -0.60    0.20     
## 11 Delusions at baseline, numeric       0.26     -0.12    0.65     
## 
## > Adjusted R^2 using Stern's equation: -0.07714111
## 
## > Model comparison:
## Analysis of Variance Table
## 
## Model 1: uku_oth ~ 1
## Model 2: uku_oth ~ group + age + gender + saei + illness_duration + cyp2d6_dep_drug + 
##     cyp2c19_dep_drug + bl_hallucinations_num + bl_delusions_num
##   Res.Df    RSS Df Sum of Sq      F Pr(>F)
## 1    166 1707.9                           
## 2    156 1614.3 10    93.515 0.9037 0.5314
## 
## > Durbin-Watson Test for independence:
##  lag Autocorrelation D-W Statistic p-value
##    1       0.1496739      1.696634   0.046
##  Alternative hypothesis: rho != 0
## 
## > Asserting the assumption of no multicollinearity:
##                           GVIF Df GVIF^(1/(2*Df))
## group                 1.076757  2        1.018660
## age                   1.822353  1        1.349945
## gender                1.048123  1        1.023779
## saei                  1.351045  1        1.162344
## illness_duration      1.607405  1        1.267835
## cyp2d6_dep_drug       1.011381  1        1.005674
## cyp2c19_dep_drug      1.033564  1        1.016644
## bl_hallucinations_num 1.448422  1        1.203504
## bl_delusions_num      1.472683  1        1.213541

## `geom_smooth()` using formula 'y ~ x'
```

© 2020 Jürgens G et al. *JAMA Network Open.*

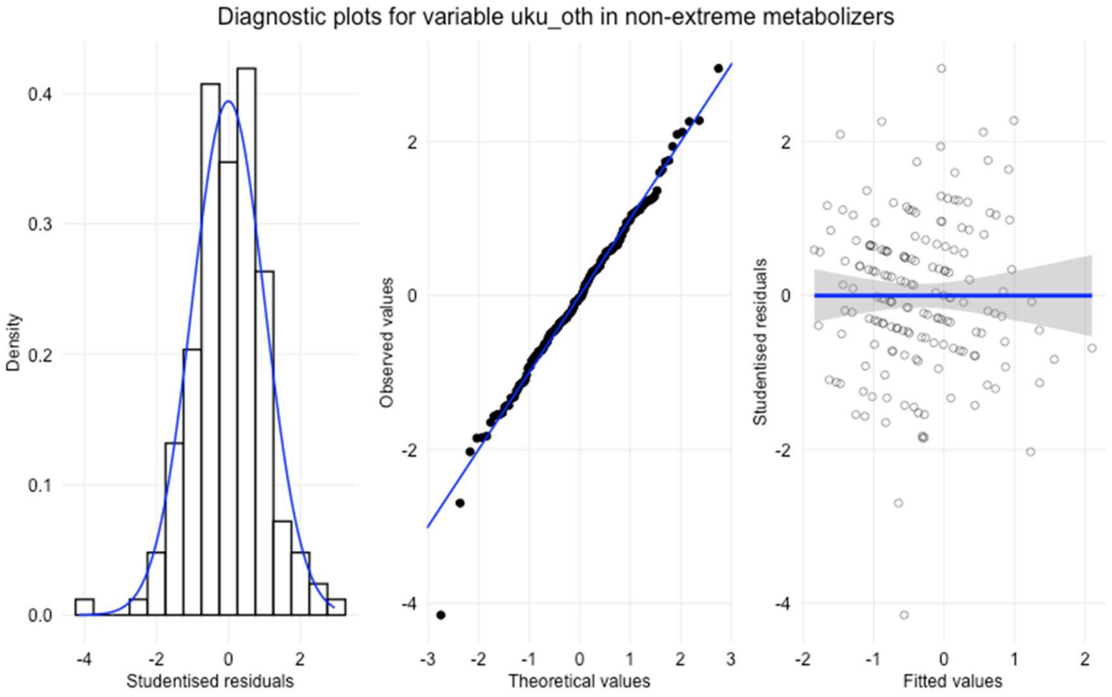

Extreme metabolizers

```
supp_4$oth_extr <- run_lin_model("oth", filter(df, extr_metabolizer == "yes"),
                                  "extreme metabolizers", FALSE)

## > Coefficients with 95% confidence intervals:
## # A tibble: 11 x 4
##    term                                 estimate conf.low conf.high
##    <chr>                                <chr>    <chr>    <chr>
##  1 (Intercept)                          0.46     -7.13    8.05
##  2 Group: CTG (vs. control)             0.94     -2.25    4.14
##  3 Group: SCM (vs. control)             -1.18    -4.59    2.22
##  4 Age                                  -0.02    -0.16    0.12
##  5 Male gender                          -1.85    -4.54    0.85
##  6 Special Assertive Early Intervention -0.44    -4.33    3.46
##  7 Duration of illness                  0.07     -0.12    0.26
##  8 CYP2D6-dependent drug use            -2.15    -5.68    1.39
##  9 CYP2C19-dependent drug use           -0.61    -4.27    3.05
## 10 Hallucinations at baseline, numeric  0.44     -0.74    1.63
## 11 Delusions at baseline, numeric       0.32     -0.78    1.42
##
## > Adjusted R^2 using Stern's equation: -0.4518885
##
## > Model comparison:
## Analysis of Variance Table
##
## Model 1: uku_oth ~ 1
## Model 2: uku_oth ~ group + age + gender + saei + illness_duration + cyp2d6_dep_drug +
##     cyp2c19_dep_drug + bl_hallucinations_num + bl_delusions_num
##   Res.Df    RSS Df Sum of Sq      F Pr(>F)
## 1     41 590.79
## 2     31 475.10 10    115.69 0.7549 0.6691
##
## > Durbin-Watson Test for independence:
```

© 2020 Jürgens G et al. *JAMA Network Open.*

```
##  lag Autocorrelation D-W Statistic p-value
##    1      -0.3001928      2.536629   0.066
##  Alternative hypothesis: rho != 0
##
## > Asserting the assumption of no multicollinearity:
##                             GVIF Df GVIF^(1/(2*Df))
## group                   1.449698  2        1.097285
## age                     2.602831  1        1.613329
## gender                  1.169655  1        1.081506
## saei                    1.683502  1        1.297498
## illness_duration        2.210197  1        1.486673
## cyp2d6_dep_drug         1.143843  1        1.069506
## cyp2c19_dep_drug        1.226047  1        1.107270
## bl_hallucinations_num 2.096487  1        1.447925
## bl_delusions_num        1.868208  1        1.366824

## `geom_smooth()` using formula 'y ~ x'
```

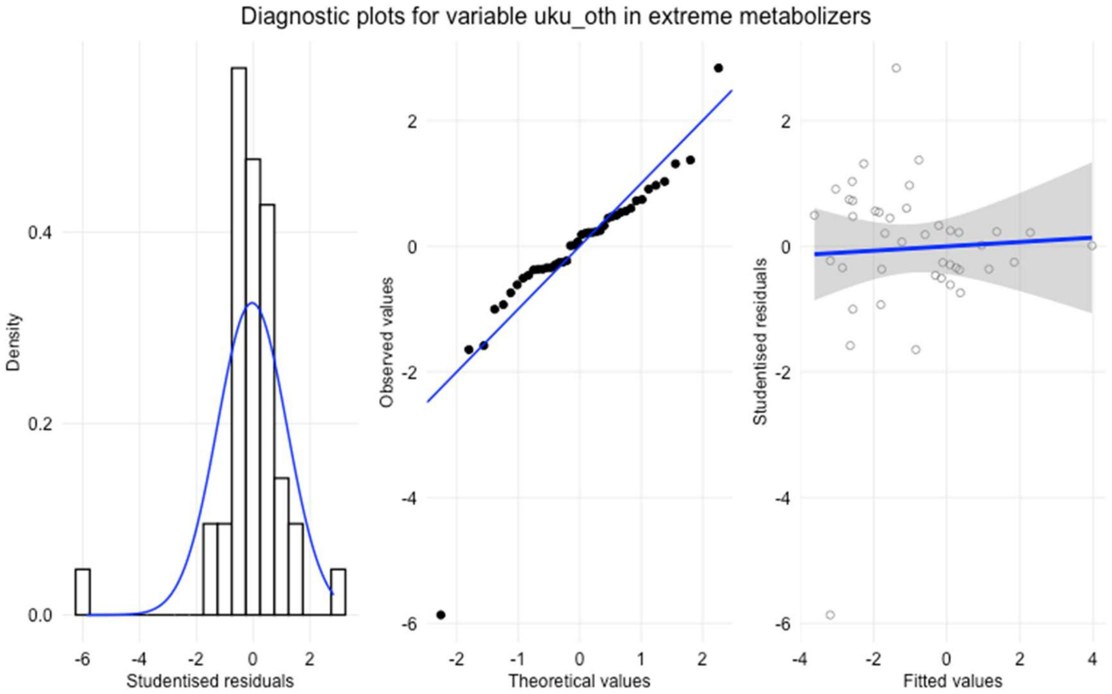

All side effects (combined)

All patients

```
supp_4$all_all <- run_lin_model("all", df, "all patients")

## > Coefficients with 95% confidence intervals:
## # A tibble: 15 x 4
##    term                                  estimate conf.low conf.high
##    <chr>                                 <chr>    <chr>    <chr>
##  1 (Intercept)                           0.41     -6.18    7.00
##  2 Group: CTG (vs. control)              0.35     -2.48    3.17
##  3 Group: SCM (vs. control)              1.61     -1.29    4.50
##  4 Age                                   -0.05    -0.18    0.08
##  5 Male gender                           0.93     -1.68    3.53
##  6 Special Assertive Early Intervention 0.34     -3.66    4.34
```

© 2020 Jürgens G et al. *JAMA Network Open.*

```
##  7 Duration of illness                  0.07     -0.10    0.24
##  8 CYP2D6-dependent drug use            0.00     -3.02    3.03
##  9 CYP2C19-dependent drug use           1.45     -1.28    4.17
## 10 Hallucinations at baseline, numeric  0.07     -0.86    1.00
## 11 Delusions at baseline, numeric       -0.11    -1.01    0.80
## 12 Poor metabolizer                     3.14     -1.54    7.82
## 13 Fast metabolizer                     -3.17    -13.24   6.89
## 14 Poor metabolizer + male              -4.97    -11.23   1.29
## 15 Fast metabolizer + male              -4.22    -17.60   9.16
##
## > Adjusted R^2 using Stern's equation: -0.1173423
##
## > Model comparison:
## Analysis of Variance Table
##
## Model 1: uku_all ~ 1
## Model 2: uku_all ~ group + age + gender + saei + illness_duration + cyp2d6_dep_drug +
##     cyp2c19_dep_drug + bl_hallucinations_num + bl_delusions_num +
##     poor_metabolizer + fast_metabolizer + gender * poor_metabolizer +
##     gender * fast_metabolizer
##   Res.Df   RSS Df Sum of Sq      F Pr(>F)
## 1    208 14234
## 2    194 13622 14    611.22 0.6218 0.8452
##
## > Durbin-Watson Test for independence:
##  lag Autocorrelation D-W Statistic p-value
##    1      0.06019163      1.876686   0.356
##  Alternative hypothesis: rho != 0
##
## > Asserting the assumption of no multicollinearity:
##                             GVIF Df GVIF^(1/(2*Df))
## group                   1.075606  2        1.018388
## age                     1.978612  1        1.406632
## gender                  1.293384  1        1.137271
## saei                    1.461560  1        1.208950
## illness_duration        1.663455  1        1.289750
## cyp2d6_dep_drug         1.020856  1        1.010374
## cyp2c19_dep_drug        1.035240  1        1.017467
## bl_hallucinations_num   1.483038  1        1.217800
## bl_delusions_num        1.511205  1        1.229311
## poor_metabolizer        2.341479  1        1.530189
## fast_metabolizer        2.509067  1        1.584003
## gender:poor_metabolizer 2.595187  1        1.610958
## gender:fast_metabolizer 2.570398  1        1.603246

## `geom_smooth()` using formula 'y ~ x'
```

© 2020 Jürgens G et al. *JAMA Network Open.*

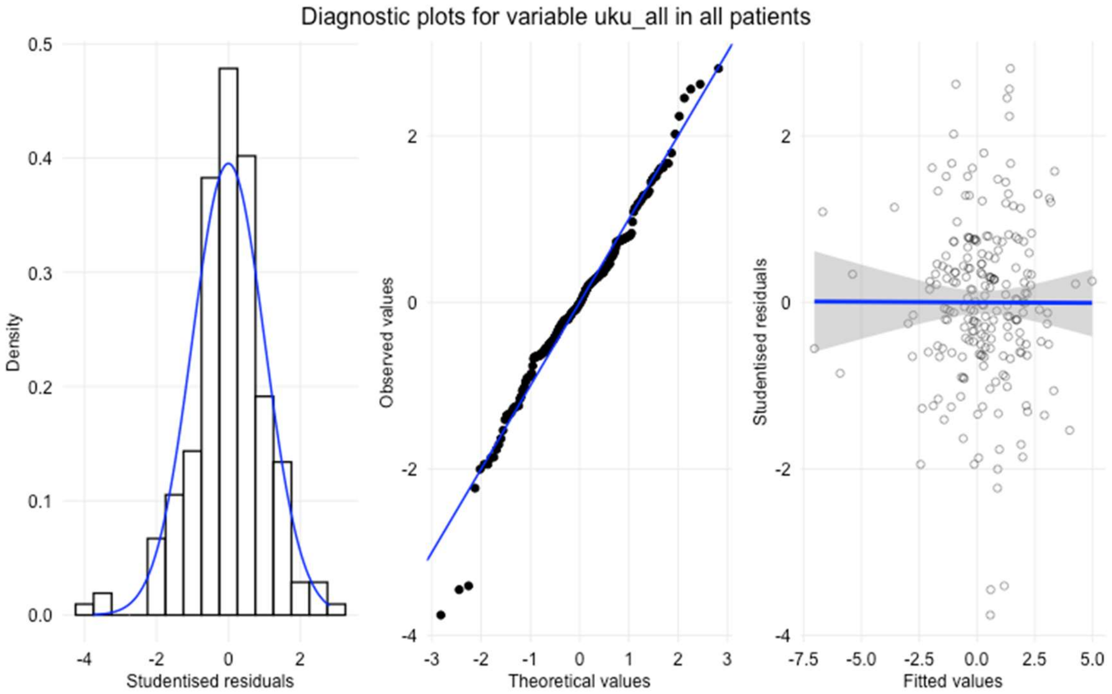

Non-extreme metabolizers

```
supp_4$all_non_extr <- run_lin_model("all", filter(df, extr_metabolizer == "no"),
                                     "non-extreme metabolizers", FALSE)

## > Coefficients with 95% confidence intervals:
## # A tibble: 11 x 4
##    term                                   estimate conf.low conf.high
##    <chr>                                  <chr>    <chr>    <chr>
##  1 (Intercept)                            -1.76    -9.27    5.75
##  2 Group: CTG (vs. control)               -0.39    -3.65    2.87
##  3 Group: SCM (vs. control)               2.18     -1.10    5.45
##  4 Age                                    0.01     -0.14    0.17
##  5 Male gender                            1.11     -1.56    3.79
##  6 Special Assertive Early Intervention -1.19     -5.86    3.48
##  7 Duration of illness                    -0.00    -0.20    0.20
##  8 CYP2D6-dependent drug use              -0.32    -3.73    3.10
##  9 CYP2C19-dependent drug use             2.29     -0.74    5.33
## 10 Hallucinations at baseline, numeric    0.01     -1.04    1.07
## 11 Delusions at baseline, numeric         0.08     -0.94    1.09
##
## > Adjusted R^2 using Stern's equation: -0.09476252
##
## > Model comparison:
## Analysis of Variance Table
##
## Model 1: uku_all ~ 1
## Model 2: uku_all ~ group + age + gender + saei + illness_duration + cyp2d6_dep_drug +
##     cyp2c19_dep_drug + bl_hallucinations_num + bl_delusions_num
##   Res.Df   RSS Df Sum of Sq     F Pr(>F)
## 1    166 11827
## 2    156 11362 10    464.71 0.638 0.7795
##
## > Durbin-Watson Test for independence:
```

© 2020 Jürgens G et al. *JAMA Network Open.*

```
##  lag Autocorrelation D-W Statistic p-value
##    1      0.03600505      1.926184   0.538
##  Alternative hypothesis: rho != 0
##
## > Asserting the assumption of no multicollinearity:
##                            GVIF Df GVIF^(1/(2*Df))
## group                  1.076757  2        1.018660
## age                    1.822353  1        1.349945
## gender                 1.048123  1        1.023779
## saei                   1.351045  1        1.162344
## illness_duration       1.607405  1        1.267835
## cyp2d6_dep_drug        1.011381  1        1.005674
## cyp2c19_dep_drug       1.033564  1        1.016644
## bl_hallucinations_num 1.448422  1        1.203504
## bl_delusions_num       1.472683  1        1.213541

## `geom_smooth()` using formula 'y ~ x'
```

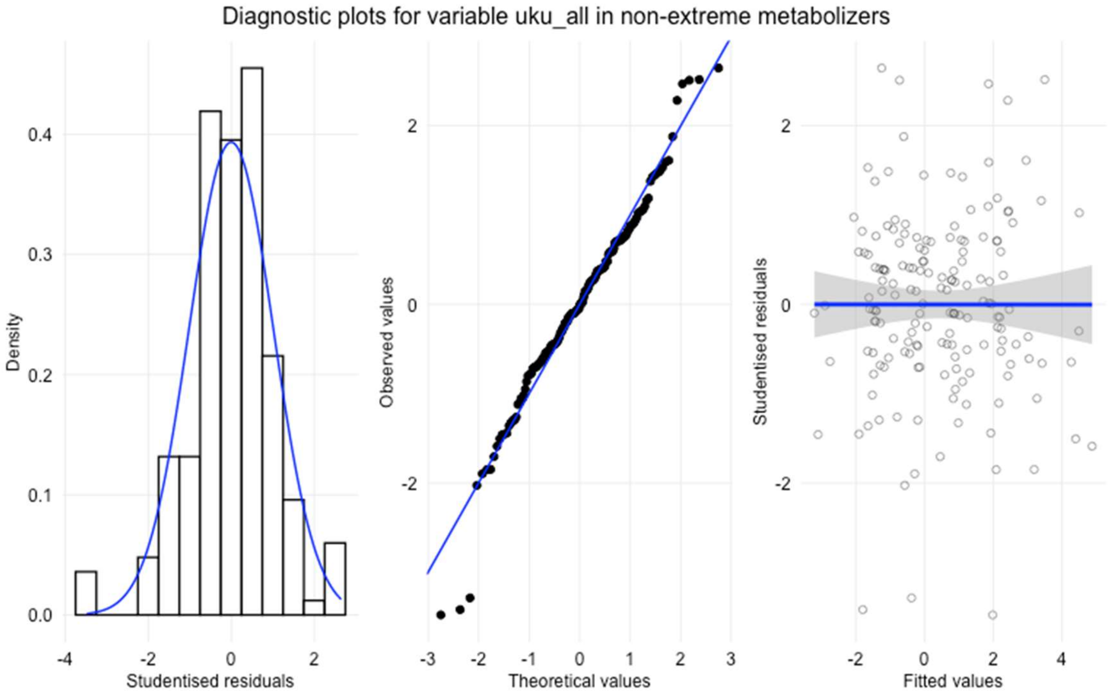

Extreme metabolizers

```
supp_4$all_extr <- run_lin_model("all", filter(df, extr_metabolizer == "yes"),
                                 "extreme metabolizers", FALSE)

## > Coefficients with 95% confidence intervals:
## # A tibble: 11 x 4
##    term                                estimate conf.low conf.high
##    <chr>                               <chr>    <chr>    <chr>
##  1 (Intercept)                         7.27     -7.70    22.25
##  2 Group: CTG (vs. control)            2.38     -3.93    8.69
##  3 Group: SCM (vs. control)            -0.06    -6.77    6.65
##  4 Age                                 -0.19    -0.46    0.08
##  5 Male gender                         -4.42    -9.73    0.89
##  6 Special Assertive Early Intervention 1.94     -5.74    9.62
##  7 Duration of illness                 0.21     -0.17    0.59
```

© 2020 Jürgens G et al. *JAMA Network Open.*

```
##  8 CYP2D6-dependent drug use            0.16     -6.81    7.13
##  9 CYP2C19-dependent drug use           -2.16    -9.38    5.06
## 10 Hallucinations at baseline, numeric  -0.06    -2.40    2.27
## 11 Delusions at baseline, numeric       -0.12    -2.29    2.05
##
## > Adjusted R^2 using Stern's equation: -0.3982815
##
## > Model comparison:
## Analysis of Variance Table
##
## Model 1: uku_all ~ 1
## Model 2: uku_all ~ group + age + gender + saei + illness_duration + cyp2d6_dep_drug +
##     cyp2c19_dep_drug + bl_hallucinations_num + bl_delusions_num
##   Res.Df    RSS Df Sum of Sq      F Pr(>F)
## 1     41 2385.6
## 2     31 1847.7 10    537.99 0.9026 0.5423
##
## > Durbin-Watson Test for independence:
##  lag Autocorrelation D-W Statistic p-value
##    1      -0.2276709      2.405106   0.132
##  Alternative hypothesis: rho != 0
##
## > Asserting the assumption of no multicollinearity:
##                           GVIF Df GVIF^(1/(2*Df))
## group                 1.449698  2        1.097285
## age                   2.602831  1        1.613329
## gender                1.169655  1        1.081506
## saei                  1.683502  1        1.297498
## illness_duration      2.210197  1        1.486673
## cyp2d6_dep_drug       1.143843  1        1.069506
## cyp2c19_dep_drug      1.226047  1        1.107270
## bl_hallucinations_num 2.096487  1        1.447925
## bl_delusions_num      1.868208  1        1.366824

## `geom_smooth()` using formula 'y ~ x'
```

© 2020 Jürgens G et al. *JAMA Network Open.*

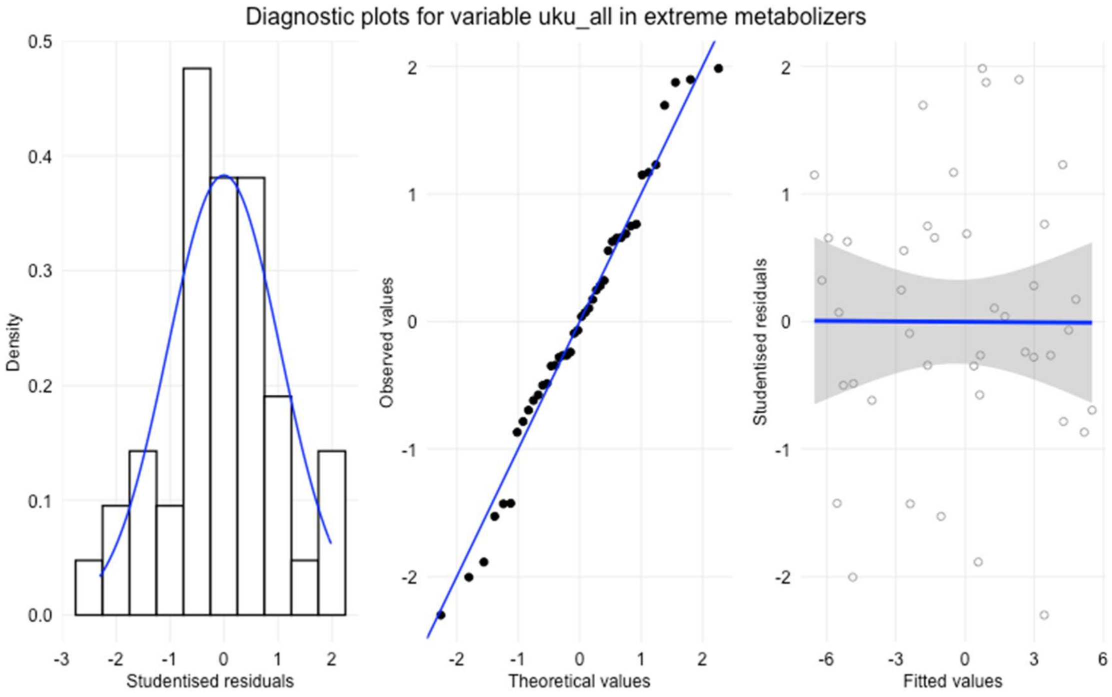

Non-ultra rapid metabolizers

This was incorporated to address a question raised during review. We fit the UKU linear model to all non-ultra-rapid metabolizers to gauge how this affects the resultant parameters.

```
supp_4$all_non_um <- run_lin_model("all", filter(df, cyp2d6_pheno != "UM"),
                                   "non-ultra-rapid metabolizers")

## > Coefficients with 95% confidence intervals:
## # A tibble: 13 x 4
##    term                                estimate conf.low conf.high
##    <chr>                               <chr>    <chr>    <chr>
##  1 (Intercept)                         0.69     -5.96    7.34
##  2 Group: CTG (vs. control)            0.02     -2.85    2.90
##  3 Group: SCM (vs. control)            1.76     -1.18    4.70
##  4 Age                                 -0.05    -0.18    0.08
##  5 Male gender                         0.93     -1.68    3.55
##  6 Special Assertive Early Intervention 0.22    -3.91    4.34
##  7 Duration of illness                 0.06     -0.12    0.24
##  8 CYP2D6-dependent drug use           -0.01    -3.05    3.02
##  9 CYP2C19-dependent drug use          1.35     -1.41    4.12
## 10 Hallucinations at baseline, numeric 0.06     -0.89    1.00
## 11 Delusions at baseline, numeric      -0.11    -1.02    0.81
## 12 Poor metabolizer                    3.14     -1.56    7.84
## 13 Poor metabolizer + male             -4.93    -11.22   1.35
##
## > Adjusted R^2 using Stern's equation: -0.1130154
##
## > Model comparison:
## Analysis of Variance Table
##
## Model 1: uku_all ~ 1
## Model 2: uku_all ~ group + age + gender + saei + illness_duration + cyp2d6_dep_drug +
```

© 2020 Jürgens G et al. *JAMA Network Open.*

```
##     cyp2c19_dep_drug + bl_hallucinations_num + bl_delusions_num +
##     poor_metabolizer + gender * poor_metabolizer
##   Res.Df   RSS Df Sum of Sq      F Pr(>F)
## 1    201 13796
## 2    189 13362 12    433.64 0.5111  0.906
##
## > Durbin-Watson Test for independence:
##  lag Autocorrelation D-W Statistic p-value
##    1      0.04536468      1.908522   0.482
##  Alternative hypothesis: rho != 0
##
## > Asserting the assumption of no multicollinearity:
##                             GVIF Df GVIF^(1/(2*Df))
## group                   1.067594  2        1.016486
## age                     1.903791  1        1.379779
## gender                  1.251628  1        1.118762
## saei                    1.401036  1        1.183654
## illness_duration        1.628866  1        1.276270
## cyp2d6_dep_drug         1.013377  1        1.006666
## cyp2c19_dep_drug        1.030309  1        1.015041
## bl_hallucinations_num   1.441532  1        1.200638
## bl_delusions_num        1.490214  1        1.220743
## poor_metabolizer        2.326130  1        1.525165
## gender:poor_metabolizer 2.587393  1        1.608538

## `geom_smooth()` using formula 'y ~ x'
```

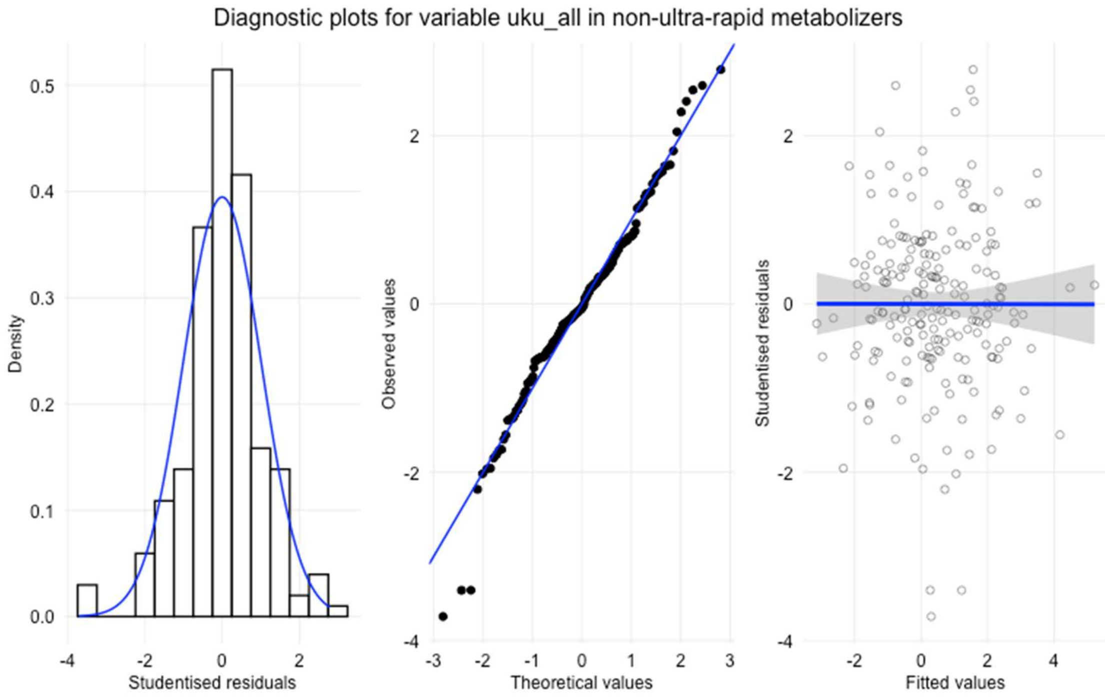

Table 4

Table 4 is a subset of the table in supplemental 4 (separate file).

```
supplemental_4 <- mutate(bind_rows(supp_4),
                         coef = sprintf("%s (%s; %s)", estimate, conf.low, conf.high)) %>%
```

© 2020 Jürgens G et al. *JAMA Network Open.*

```r
  select(outcome, subset, term, coef) %>%
  spread(outcome, coef) %>%
  setNames(sapply(names(.), first_up)) # cosmetics
save(supplemental_4, file = "supplemental_4.RData")

filter(bind_rows(supp_4), subset == "All patients") %>%
  mutate(ci = sprintf("%s to %s", conf.low, conf.high)) %>%
  pivot_wider(id_cols = c(term), names_from = outcome, values_from = c(estimate, ci)) %>%
  write.table("intermediate_for_excel.tsv", sep = "\t")
```

Compare results from full data set with those of non-ultra-rapid metabolizers

```r
filter(supplemental_4, Subset %in% c("All patients", "Non-ultra-rapid metabolizers")) %>%
  select(Subset, Term, All) %>%
  spread(Subset, All)

## # A tibble: 15 x 3
##    Term                                `All patients`       `Non-ultra-rapid metabolizers`
##    <chr>                               <chr>                <chr>
##  1 (Intercept)                         0.41 (-6.18; 7.00)   0.69 (-5.96; 7.34)
##  2 Age                                 -0.05 (-0.18; 0.08)  -0.05 (-0.18; 0.08)
##  3 CYP2C19-dependent drug use          1.45 (-1.28; 4.17)   1.35 (-1.41; 4.12)
##  4 CYP2D6-dependent drug use           0.00 (-3.02; 3.03)   -0.01 (-3.05; 3.02)
##  5 Delusions at baseline, numeric      -0.11 (-1.01; 0.80)  -0.11 (-1.02; 0.81)
##  6 Duration of illness                 0.07 (-0.10; 0.24)   0.06 (-0.12; 0.24)
##  7 Fast metabolizer                    -3.17 (-13.24; 6.89) <NA>
##  8 Fast metabolizer + male             -4.22 (-17.60; 9.16) <NA>
##  9 Group: CTG (vs. control)            0.35 (-2.48; 3.17)   0.02 (-2.85; 2.90)
## 10 Group: SCM (vs. control)            1.61 (-1.29; 4.50)   1.76 (-1.18; 4.70)
## 11 Hallucinations at baseline, numeric 0.07 (-0.86; 1.00)   0.06 (-0.89; 1.00)
## 12 Male gender                         0.93 (-1.68; 3.53)   0.93 (-1.68; 3.55)
## 13 Poor metabolizer                    3.14 (-1.54; 7.82)   3.14 (-1.56; 7.84)
## 14 Poor metabolizer + male             -4.97 (-11.23; 1.29) -4.93 (-11.22; 1.35)
## 15 Special Assertive Early Intervention 0.34 (-3.66; 4.34)  0.22 (-3.91; 4.34)
```

Sensitivity to misclassification of metabolizer status

We re-classify sampled fractions of patients with predicted normal metabolizer phenotype to extreme metabolizer phenotype; we test the impact of increasing misclassification through a grid-search approach. Then, we plot the distributions of coefficients, colour-coded by level of reclassification. The vertical, dotted line represents the original coefficient from the model using recorded metabolizer status (= the one used in the all analyses).

```r
o <- list()
non_extr <- df$extr_metabolizer == "no"
for (p in seq(5, 40, by = 5) / 100) { # p is proportion to reclassify
  l <- list()
  for (i in 1:200) { # number of iterations
    new_extr_var <- df$extr_metabolizer
    set.seed(42 + i) # for reproducibility; add i to give each iteration its own seed
    new_extr_var[non_extr] <- sample(c("no", "yes"), sum(non_extr), replace = TRUE, prob = c(1 - p,
p))
    mod <- update(cox_model_extr_base, data = df, subset = new_extr_var == "yes")
    l[[i]] <- exp(mod$coefficients) # exponentiate to yield hazard ratios
  }
  o[[paste(p)]] <- data.frame(do.call(rbind, l), p_key = paste0(p * 100, "%"))
}

facet_names <- c(groupctg = "Open CYP test vs. control",
                 groupscm = "Structured clinical monitoring vs. control")
```

```
extr_coefs <- tibble::enframe(cox_model_extr_base$coefficients) %>%
  transmute(variable = facet_names[name],
            coef = exp(value))

do.call(rbind, o) %>%
  gather(variable, sampled_coef, -p_key) %>%
  mutate(variable = facet_names[as.character(variable)]) %>%
  ggplot(aes(x = sampled_coef, colour = p_key)) +
    geom_vline(aes(xintercept = coef), extr_coefs, linetype = 2, size = 0.4) +
    stat_density(geom = "line", alpha = 0.6, position = "identity") +
    labs(x = "Estimated HR (log scale)", y = "Frequency of sampled HR estimate",
         colour = "Re-classified", title = "Densities of estimated HR by classification proportion")
+
    facet_wrap(~ variable, scales = "free") +
    scale_x_continuous(breaks = c(0.5, 0.67, 0.8, 1, 1.25, 1.5, 2), trans = "log") +
    coord_cartesian(xlim = c(0.5, 2)) +
    scale_colour_discrete(h = c(0, 360), h.start = 200) +
    theme(legend.position = "bottom", title = element_text(size = text_size),
          text = element_text(size = text_size), axis.text.y = element_blank(),
          axis.ticks.y = element_blank(), axis.title.y = element_blank(),
          axis.text.x = element_text(size = text_size), strip.text = element_text(size = text_size))
+
    guides(colour = guide_legend(nrow = 1, byrow = TRUE))
```

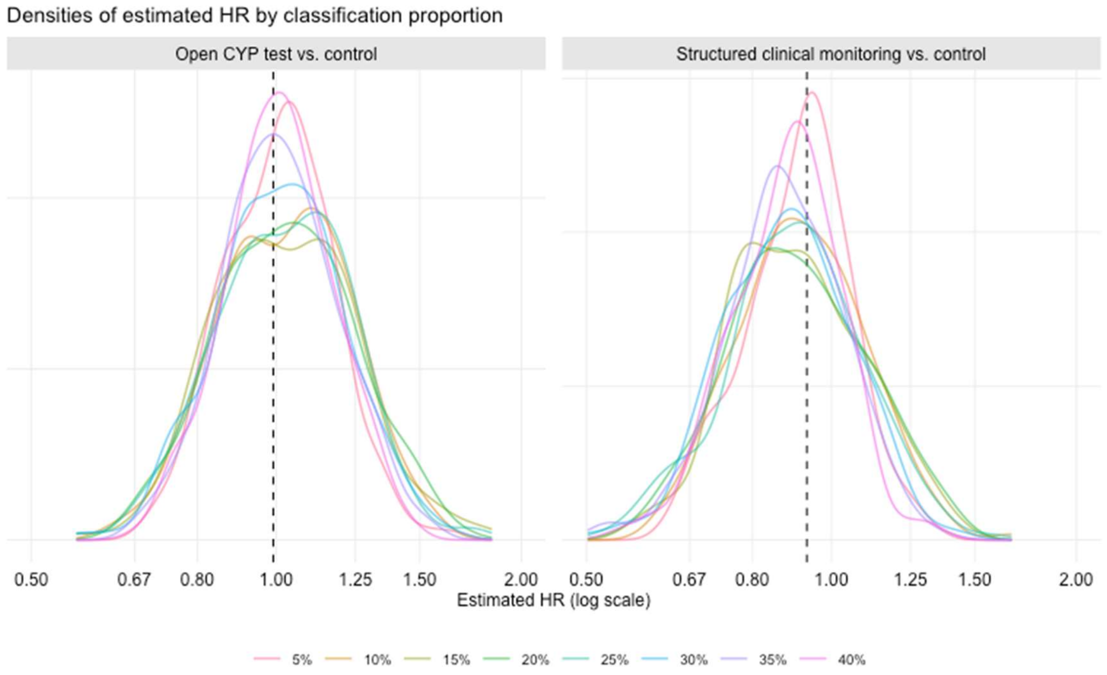

R session

Versions of R and packages (including dependencies) used in the analysis.

```
options(width = 80) # To prevent crazy output from session_info()
devtools::session_info(pkgs = packages, include_base = FALSE)
```

© 2020 Jürgens G et al. *JAMA Network Open.*

```
## ─ Session info ───────────────────────────────────────────────────────────────
##  setting  value
##  version  R version 3.6.3 (2020-02-29)
##  os       macOS Catalina 10.15
##  system   x86_64, darwin15.6.0
##  ui       X11
##  language (EN)
##  collate  en_US.UTF-8
##  ctype    en_US.UTF-8
##  tz       Europe/Copenhagen
##  date     2020-09-10
##
## ─ Packages ───────────────────────────────────────────────────────────────────
##  package        * version    date       lib source
##  abind            1.4-5      2016-07-21 [1] CRAN (R 3.6.0)
##  assertthat       0.2.1      2019-03-21 [1] CRAN (R 3.6.0)
##  backports        1.1.7      2020-05-13 [1] CRAN (R 3.6.2)
##  BH               1.72.0-3   2020-01-08 [1] CRAN (R 3.6.0)
##  boot             1.3-24     2019-12-20 [1] CRAN (R 3.6.3)
##  broom          * 0.7.0      2020-07-09 [1] CRAN (R 3.6.2)
##  callr            3.4.3      2020-03-28 [1] CRAN (R 3.6.2)
##  car            * 3.0-9      2020-08-11 [1] CRAN (R 3.6.2)
##  carData        * 3.0-3      2019-11-16 [1] CRAN (R 3.6.0)
##  cellranger       1.1.0      2016-07-27 [1] CRAN (R 3.6.0)
##  class            7.3-15     2019-01-01 [1] CRAN (R 3.6.3)
##  cli              2.0.2      2020-02-28 [1] CRAN (R 3.6.0)
##  clipr            0.7.0      2019-07-23 [1] CRAN (R 3.6.0)
##  colorspace     * 1.4-1      2019-03-18 [1] CRAN (R 3.6.0)
##  corrplot         0.84       2017-10-16 [1] CRAN (R 3.6.0)
##  cowplot          1.0.0      2019-07-11 [1] CRAN (R 3.6.0)
##  cpp11            0.2.1      2020-08-11 [1] CRAN (R 3.6.2)
##  crayon           1.3.4      2017-09-16 [1] CRAN (R 3.6.0)
##  curl             4.3        2019-12-02 [1] CRAN (R 3.6.0)
##  data.table       1.12.8     2019-12-09 [1] CRAN (R 3.6.0)
##  DBI              1.1.0      2019-12-15 [1] CRAN (R 3.6.0)
##  DEoptimR         1.0-8      2016-11-19 [1] CRAN (R 3.6.0)
##  desc             1.2.0      2018-05-01 [1] CRAN (R 3.6.0)
##  descr          * 1.1.4      2018-01-19 [1] CRAN (R 3.6.0)
##  digest           0.6.25     2020-02-23 [1] CRAN (R 3.6.0)
##  dplyr          * 1.0.1      2020-07-31 [1] CRAN (R 3.6.2)
##  e1071            1.7-3      2019-11-26 [1] CRAN (R 3.6.0)
##  ellipsis         0.3.1      2020-05-15 [1] CRAN (R 3.6.2)
##  evaluate         0.14       2019-05-28 [1] CRAN (R 3.6.0)
##  exactRankTests   0.8-31     2019-12-11 [1] CRAN (R 3.6.0)
##  fansi            0.4.1      2020-01-08 [1] CRAN (R 3.6.0)
##  farver           2.0.3      2020-01-16 [1] CRAN (R 3.6.0)
##  forcats        * 0.5.0      2020-03-01 [1] CRAN (R 3.6.0)
##  foreign          0.8-75     2020-01-20 [1] CRAN (R 3.6.3)
##  gdata            2.18.0     2017-06-06 [1] CRAN (R 3.6.0)
##  generics         0.0.2      2018-11-29 [1] CRAN (R 3.6.0)
##  ggplot2        * 3.3.1      2020-05-28 [1] CRAN (R 3.6.2)
##  ggpubr         * 0.4.0      2020-06-27 [1] CRAN (R 3.6.2)
##  ggrepel          0.8.2      2020-03-08 [1] CRAN (R 3.6.0)
##  ggsci            2.9        2018-05-14 [1] CRAN (R 3.6.0)
##  ggsignif         0.6.0      2019-08-08 [1] CRAN (R 3.6.0)
##  glue             1.4.1      2020-05-13 [1] CRAN (R 3.6.2)
##  gmodels          2.18.1     2018-06-25 [1] CRAN (R 3.6.0)
##  gridExtra      * 2.3        2017-09-09 [1] CRAN (R 3.6.0)
##  gtable           0.3.0      2019-03-25 [1] CRAN (R 3.6.0)
```

© 2020 Jürgens G et al. *JAMA Network Open.*

```
## gtools          3.8.2      2020-03-31 [1] CRAN (R 3.6.2)
## haven           2.3.1      2020-06-01 [1] CRAN (R 3.6.2)
## highr           0.8        2019-03-20 [1] CRAN (R 3.6.0)
## hms             0.5.3      2020-01-08 [1] CRAN (R 3.6.0)
## isoband         0.2.1      2020-04-12 [1] CRAN (R 3.6.2)
## km.ci           0.5-2      2009-08-30 [1] CRAN (R 3.6.0)
## KMsurv          0.1-5      2012-12-03 [1] CRAN (R 3.6.0)
## knitr         * 1.29       2020-06-23 [1] CRAN (R 3.6.2)
## labeling        0.3        2014-08-23 [1] CRAN (R 3.6.0)
## labelled        2.5.0      2020-06-17 [1] CRAN (R 3.6.2)
## laeken          0.5.1      2020-02-05 [1] CRAN (R 3.6.0)
## lattice         0.20-38    2018-11-04 [1] CRAN (R 3.6.3)
## lifecycle       0.2.0      2020-03-06 [1] CRAN (R 3.6.0)
## lme4            1.1-23     2020-04-07 [1] CRAN (R 3.6.2)
## lmtest          0.9-37     2019-04-30 [1] CRAN (R 3.6.0)
## magrittr        1.5        2014-11-22 [1] CRAN (R 3.6.0)
## maptools        1.0-1      2020-05-14 [1] CRAN (R 3.6.2)
## markdown        1.1        2019-08-07 [1] CRAN (R 3.6.0)
## MASS          * 7.3-52     2020-08-18 [1] CRAN (R 3.6.3)
## Matrix          1.2-18     2019-11-27 [1] CRAN (R 3.6.3)
## MatrixModels    0.4-1      2015-08-22 [1] CRAN (R 3.6.0)
## maxstat         0.7-25     2017-03-02 [1] CRAN (R 3.6.0)
## mgcv            1.8-31     2019-11-09 [1] CRAN (R 3.6.0)
## mice          * 3.11.0     2020-08-05 [1] CRAN (R 3.6.2)
## mime            0.9        2020-02-04 [1] CRAN (R 3.6.0)
## minqa           1.2.4      2014-10-09 [1] CRAN (R 3.6.0)
## mitools         2.4        2019-04-26 [1] CRAN (R 3.6.0)
## munsell         0.5.0      2018-06-12 [1] CRAN (R 3.6.0)
## mvtnorm         1.1-0      2020-02-24 [1] CRAN (R 3.6.0)
## nlme            3.1-144    2020-02-06 [1] CRAN (R 3.6.3)
## nloptr          1.2.2.1    2020-03-11 [1] CRAN (R 3.6.0)
## nnet            7.3-12     2016-02-02 [1] CRAN (R 3.6.3)
## numDeriv        2016.8-1.1 2019-06-06 [1] CRAN (R 3.6.0)
## openxlsx        4.1.5      2020-05-06 [1] CRAN (R 3.6.2)
## pbkrtest        0.4-8.6    2020-02-20 [1] CRAN (R 3.6.0)
## pillar          1.4.4      2020-05-05 [1] CRAN (R 3.6.2)
## pkgbuild        1.0.8      2020-05-07 [1] CRAN (R 3.6.2)
## pkgconfig       2.0.3      2019-09-22 [1] CRAN (R 3.6.0)
## pkgload         1.1.0      2020-05-29 [1] CRAN (R 3.6.2)
## polynom         1.4-0      2019-03-22 [1] CRAN (R 3.6.0)
## praise          1.0.0      2015-08-11 [1] CRAN (R 3.6.0)
## prettyunits     1.1.1      2020-01-24 [1] CRAN (R 3.6.0)
## processx        3.4.2      2020-02-09 [1] CRAN (R 3.6.0)
## progress        1.2.2      2019-05-16 [1] CRAN (R 3.6.0)
## ps              1.3.3      2020-05-08 [1] CRAN (R 3.6.2)
## purrr           0.3.4      2020-04-17 [1] CRAN (R 3.6.2)
## quantreg        5.55       2020-04-01 [1] CRAN (R 3.6.2)
## R6              2.4.1      2019-11-12 [1] CRAN (R 3.6.0)
## ranger          0.12.1     2020-01-10 [1] CRAN (R 3.6.0)
## RColorBrewer    1.1-2      2014-12-07 [1] CRAN (R 3.6.0)
## Rcpp            1.0.4.8    2020-04-21 [1] local
## RcppEigen       0.3.3.7.0  2019-11-16 [1] CRAN (R 3.6.0)
## readr           1.3.1      2018-12-21 [1] CRAN (R 3.6.0)
## readxl          1.3.1      2019-03-13 [1] CRAN (R 3.6.0)
## rematch         1.0.1      2016-04-21 [1] CRAN (R 3.6.0)
## rio             0.5.16     2018-11-26 [1] CRAN (R 3.6.0)
## rlang           0.4.7      2020-07-09 [1] CRAN (R 3.6.2)
## robustbase      0.93-6     2020-03-23 [1] CRAN (R 3.6.0)
## rprojroot       1.3-2      2018-01-03 [1] CRAN (R 3.6.0)
```

© 2020 Jürgens G et al. *JAMA Network Open.*

```
##  rstatix          0.6.0      2020-06-18 [1] CRAN (R 3.6.2)
##  rstudioapi       0.11       2020-02-07 [1] CRAN (R 3.6.0)
##  scales           1.1.1      2020-05-11 [1] CRAN (R 3.6.2)
##  sp               1.4-1      2020-02-28 [1] CRAN (R 3.6.0)
##  SparseM          1.78       2019-12-13 [1] CRAN (R 3.6.0)
##  statmod          1.4.34     2020-02-17 [1] CRAN (R 3.6.0)
##  stringi          1.4.6      2020-02-17 [1] CRAN (R 3.6.0)
##  stringr          1.4.0      2019-02-10 [1] CRAN (R 3.6.0)
##  survey           4.0        2020-04-03 [1] CRAN (R 3.6.2)
##  survival       * 3.2-3      2020-06-13 [1] CRAN (R 3.6.2)
##  survminer      * 0.4.8      2020-07-25 [1] CRAN (R 3.6.2)
##  survMisc         0.5.5      2018-07-05 [1] CRAN (R 3.6.0)
##  tableone       * 0.12.0     2020-07-26 [1] CRAN (R 3.6.3)
##  testthat         2.3.2      2020-03-02 [1] CRAN (R 3.6.0)
##  tibble         * 3.0.1      2020-04-20 [1] CRAN (R 3.6.2)
##  tidyr          * 1.1.1      2020-07-31 [1] CRAN (R 3.6.2)
##  tidyselect       1.1.0      2020-05-11 [1] CRAN (R 3.6.2)
##  utf8             1.1.4      2018-05-24 [1] CRAN (R 3.6.0)
##  vcd              1.4-7      2020-04-02 [1] CRAN (R 3.6.2)
##  vctrs            0.3.2      2020-07-15 [1] CRAN (R 3.6.2)
##  VIM            * 6.0.0      2020-05-08 [1] CRAN (R 3.6.3)
##  viridisLite      0.3.0      2018-02-01 [1] CRAN (R 3.6.0)
##  withr            2.2.0      2020-04-20 [1] CRAN (R 3.6.3)
##  xfun             0.16       2020-07-24 [1] CRAN (R 3.6.2)
##  xtable           1.8-4      2019-04-21 [1] CRAN (R 3.6.0)
##  yaml             2.2.1      2020-02-01 [1] CRAN (R 3.6.0)
##  zip              2.0.4      2019-09-01 [1] CRAN (R 3.6.0)
##  zoo              1.8-7      2020-01-10 [1] CRAN (R 3.6.0)
##
## [1] /Library/Frameworks/R.framework/Versions/3.6/Resources/library
```
