# Supplementary material for: Effect of Routine Cytochrome P450 2D6 and 2C19 Genotyping on Antipsychotic Drug Persistence in Patients With Schizophrenia: A Randomized Clinical Trial
Source: JAMA Netw Open. 2020 Dec 7;3(12):e2027909. doi: 10.1001/jamanetworkopen.2020.27909 (PMC12520709; doi:10.1001/jamanetworkopen.2020.27909)
Supplement: Supplement 3. — Data Sharing Statement [file jamanetwopen-e2027909-s003.pdf]

Data Sharing Statement

Jürgens. Effect of Routine Cytochrome P450 2D6 and 2C19 Genotyping on Antipsychotic Drug Persistence in Patients With Schizophrenia. *JAMA Netw Open*. Published December 07, 2020. doi:10.1001/jamanetworkopen.2020.27909

Data
Data available: Yes
Data types: Deidentified participant data
How to access data: gju@regionsjaelland.dk
When available: With publication

Supporting Documents
Document types: Statistical/analytic code
How to access documents: Supplement 1: Analysis Notebook
When available: With publication

Additional Information
Who can access the data: Researchers whose proposed use of the data has been approved
Types of analyses: Meta-analysis of individual participant data
Mechanisms of data availability: With investigator support, after approval of a proposal, and with a signed data access agreement
Any additional restrictions: There can be restrictions and conditions for data sharing according to GDPR.
